# Optimal integration of microalgae production with photovoltaic panels: environmental impacts and energy balance

**DOI:** 10.1186/s13068-019-1579-4

**Published:** 2019-10-08

**Authors:** Marjorie Morales, Arnaud Hélias, Olivier Bernard

**Affiliations:** 1 INRIA BIOCORE, BP 93, 06902 Sophia Antipolis Cedex, France; 2 Laboratoire de Biotechnologie de l’Environnement, Montpellier SupAgro, INRA, Univ Montpellier, 2 Place Pierre Viala, 34060 Montpellier Cedex 1, France; 3 Elsa, Research Group for Environmental Life Cycle Sustainability Assessment, Montpellier, France; 4 Department of Energy and Process Engineering, Faculty of Engineering, Norwegian University of Science and Technology (NTNU), 7491 Trondheim, Norway

**Keywords:** Biodiesel, *Chlorococcum* sp., *Desmodesmus* sp., Life cycle assessment, Raceway, Renewable energy

## Abstract

**Background:**

Microalgae are 10 to 20 times more productive than the current agricultural biodiesel producing oleaginous crops. However, they require larger energy supplies, so that their environmental impacts remain uncertain, as illustrated by the contradictory results in the literature. Besides, solar radiation is often too high relative to the photosynthetic capacity of microalgae. This leads to photosaturation, photoinhibition, overheating and eventually induces mortality. Shadowing microalgae with solar panels would, therefore, be a promising solution for both increasing productivity during hotter periods and producing local electricity for the process. The main objective of this study is to measure, via LCA framework, the energy performance and environmental impact of microalgae biodiesel produced in a solar greenhouse, alternating optimal microalgae species and photovoltaic panel (PV) coverage. A mathematical model is simulated to investigate the microalgae productivity in raceways under meteorological conditions in Sophia Antipolis (south of France) at variable coverture percentages (0% to 90%) of CIGS solar panels on greenhouses constructed with low-emissivity (low-E) glass.

**Results:**

A trade-off must be met between electricity and biomass production, as a larger photovoltaic coverture would limit microalgae production. From an energetic point of view, the optimal configuration lies between 10 and 20% of PV coverage. Nevertheless, from an environmental point of view, the best option is 50% PV coverage. However, the difference between impact assessments obtained for 20% and 50% PV is negligible, while the NER is 48% higher for 20% PV than for 50% PV coverage. Hence, a 20% coverture of photovoltaic panels is the best scenario from an energetic and environmental point of view.

**Conclusions:**

In comparison with the cultivation of microalgae without PV, the use of photovoltaic panels triggers a synergetic effect, sourcing local electricity and reducing climate change impacts. Considering an economic approach, low photovoltaic panel coverage would probably be more attractive. However, even with a 10% area of photovoltaic panels, the environmental footprint would already significantly decrease. It is expected that significant improvements in microalgae productivity or more advanced production processes should rapidly enhance these performances.

## Background

Renewable liquid fuels are expected to play an essential role for replacing petroleum-derived transportation fuels with a viable alternative, while reducing GHG emissions. Although biodiesel from oleaginous crops and bioethanol from sugarcane are being produced in increasing amounts, their production cannot sustainably address the demand [1]. Hence, alternative sources of biomass are required to supply this increasing demand. Microalgae-based oil is currently being considered as a promising alternative raw material for biodiesel [2].

Microalgae are photosynthetic microorganisms that transform sunlight, water and carbon dioxide into chemical energy, stored as chemical bound energy, especially into lipids, carbohydrates and proteins. Oil extracted from microalgae species can then be converted into biodiesel [3]. The oil fraction in conventional agricultural oil crops is very low (around 5% of the total biomass) compared with certain species of microalgae whose oil content can exceed 60% of dry weight [1].

Microalgae have several advantages over land-based crops in terms of oil production: high biomass productivity, no competition with feed crops, possibilities to uptake industrial sources of CO_2_, possible use of brackish or seawater and reduced competition for land [2] without using herbicides or pesticides [4]. Their simple unicellular structure and high photosynthetic efficiency lead to higher oil yield per area than the best oilseed crops [5].

Despite these advantages, microalgae-based fuels are not produced at industrial scale, mainly due to their current production cost [5]. Seeking for productive microalgae strains and optimized culture conditions and allowing a high growth rate and lipid content are current research challenges [6]. The high cost and energy demand for harvesting diluted algae cells also remain a major bottleneck.

The use of microalgae for generating energy requires large-scale, low-cost production. This implies cheap, scalable reactor designs with high algal productivity. The different algal cultivation systems can be divided into two main categories, open and closed. Closed systems consist of containers, tubes or transparent plastic bags of various sizes close to the atmosphere [7], while open systems consist of natural or agitated artificial ponds and containers open to the atmosphere.

To date, most commercial plants consist of open ponds, due to their low cost and ease of construction and operation [7]. The most common technical design is the raceway pond: an oblong, looped pond mixed with a paddlewheel. However, some disadvantages of open systems have been detected, such as high evaporation rates, diffusion of CO_2_ to the atmosphere, contamination with competing species and low control of solar radiation and temperature [7]. Ponds enclosed in glass houses or plastic-covered greenhouses provide a better control of the growth environment [8]. Climate control in greenhouses contributes to maintaining a growth temperature closer to the optimal range and, therefore, enhances the productivity. In addition, it reduces water losses through evaporation as well as the risk of contamination by other algal species or grazers [9].

Light and temperature influence algal biomass productivity and lipid cell content [10–12]. High irradiance and high temperature generate an increase in triglyceride synthesis, with a more saturated fatty acid composition compared to conditions at low irradiance and/or temperature [13]. Since light and temperature vary seasonally, these factors continuously affect the lipid composition and accumulation in outdoor cultivation systems. As in the conventional agriculture, microalgae species should be alternated along the year to fit the climate and, thus, improve yearly production. Hence, the seasonal variation of lipid productivity results from several processes, which need to be accounted for in order to accurately estimate the algal oil yield.

Moreover, solar radiation is often too high relative to the photosynthetic capacity of microalgae, thus leading to photosaturation, and photoinhibition and also to overwarming eventually significantly increasing mortality [9]. Shadowing the microalgae with solar panels, therefore, turns out to be a promising solution for both increasing productivity during hotter periods and producing local electricity for the process [14]. Jez et al. [15] demonstrated an increase in economic competitiveness for microalgae biofuels when photovoltaic (PV) panels were used as a source of electricity in the facility. It is also a noteworthy option for producing algal biofuels in remote areas (typically deserts) that are far from the electric grid or difficult to access.

However, building PV panels produces greenhouse gas emissions due to energy consumption during the manufacturing processes. Investment costs on PV technology are still relatively high [16] but they are constantly decreasing due to both technology improvements and increases in production scales [17]. The most common PV technology is crystalline silicon (single-crystalline sc-Si and multi-crystalline mc-Si), followed by Cadmium-Telluride (CdTe) and Copper Indium Gallium (di) Selenide (CIGS) [17]. Therefore, the viability of PV panels combined with biomass production strongly depends on the geographical location, on local sunlight radiation and on electricity costs.

Coupling biomass production with photovoltaic electricity represents an ideal opportunity to significantly reduce environmental impacts and electrical demands for biodiesel production systems. Although this solution is technologically appealing, its sustainability can be questionable as there is a clear trade-off between electricity and biomass production, as a larger photovoltaic panels coverture would limit microalgae production. The large seasonal variations in biomass production alter the value chain as well as its environmental impacts. Quantification of the environmental impacts of algal oil production is, therefore, necessary. Life cycle assessment (LCA) is a standardized tool that provides a quantitative and scientific analysis of the environmental impacts of products and their industrial systems [18]. The main objective of this study is to measure, via LCA framework, the energy performance and environmental impacts of microalgae-based biodiesel produced in a solar greenhouse, alternating optimal microalgae species and photovoltaic panel coverture percentages, to determine the optimal energetic environmental configuration. The functional unit (FU) is 1 MJ of algal methyl ester (biodiesel), used in a conventional internal combustion automobile engine. This prospective assessment is carried out with an eco-design approach to tackle the main features of the system. In addition, four reference cases complying with similar system boundaries and allocation approaches have been provided, only as benchmarking systems and not for purposes of comparative assertions. A mathematical model is simulated to investigate the microalgae productivity in raceways under meteorological conditions in Sophia Antipolis (south of France) at variable coverture percentages (0% to 90%) of CIGS solar panels on greenhouses. Biomass productivity and electricity production results are used as input in a process sequence of a virtual facility for biodiesel production over 145 ha and, thereafter, as input to a life cycle inventory implemented into SimaPro 8 software [19]. Three aspects of microalgae production were analyzed: potential environmental impacts, energy and carbon balance.

## Methods

### System description

**Fig. 1:**
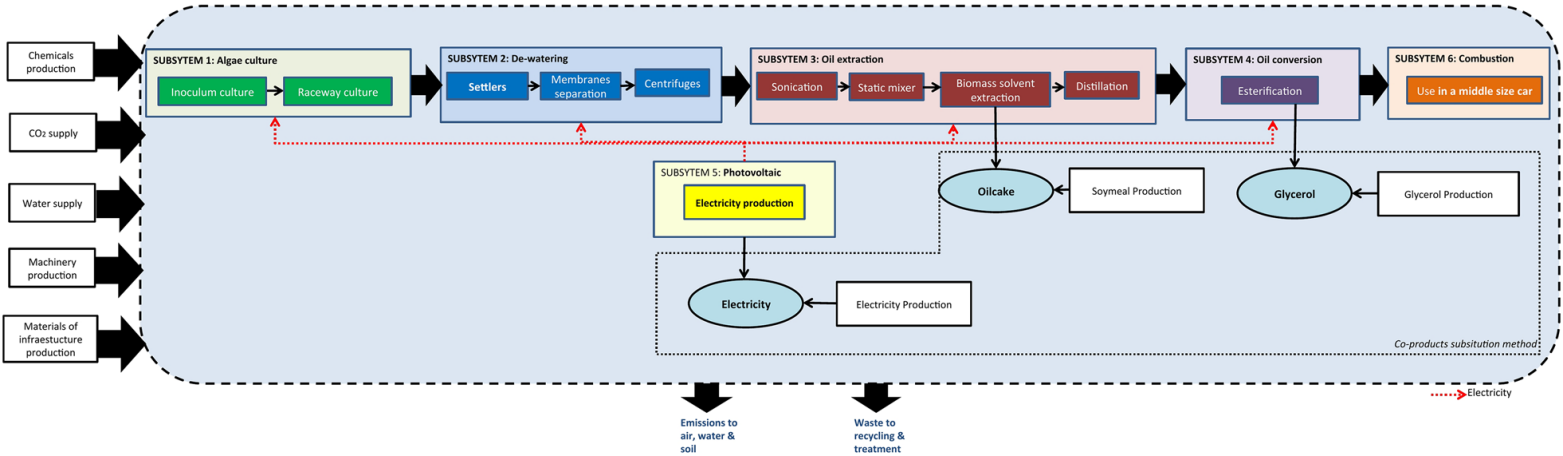
System boundaries for LCA of biodiesel production

From a ‘pond to wheel’ point of view, the scope of the system encompasses the production of biomass, process conversion and its combustion in a middle-sized car. The construction, dismantling and final disposal of the infrastructure and machinery were also included, as well as the production of chemicals and their transport. The process is divided into six main areas, also called subsystems. Figure 1 illustrates the general schematic of the system boundaries and subsystems.

Subsystem 1 considers raceway systems for microalgae biomass production coupled with upstream inoculum production operations. Subsystem 2 includes harvesting and dewatering steps, to increase the biomass solid content necessary for the subsequent conversion operations: oil extraction (Subsystem 3) and oil conversion (Subsystem 4). The design also includes the combustion of microalgae biodiesel (Subsystem 6) and photovoltaic electricity production (Subsystem 5). The infrastructure construction and machinery production and dismantling are also considered.

The size of the facility is assessed for a total production area of 145 ha (including inoculum ponds and downstream processes) composed by 5 ha “modules” representing standard greenhouses (the overall layout is described in Additional file 1: File S1, Sections 1.1).

The layout of the greenhouses within the overall facility footprint along with the pipelines and roads required for on-site circulation and transport of materials is detailed in the Additional files 1: File S1 and File S2. The production facility is located in Southern Europe (Sophia Antipolis—France, 43°36′56″N, 7°03′18″E), close enough to the Mediterranean coast to allow access to seawater. The geographic location of the facility has the highest impact on biomass productivity. The climatic conditions of the chosen location should allow for high biomass productivity throughout the year. The main factors affecting biomass productivity are the average annual irradiance level and temperature. Ideally, the temperature should be around 25 °C with minimum diurnal and seasonal variations [8]. Other considerations also have to be taken into account, such as humidity and rainfall, the possibility of storms and flood events and the presence of dust and other atmospheric pollutants [8]. Meteorological data were collected at INRA PACA, Sophia Antipolis in 2015. These data were used to simulate the dynamics of temperature and light in the cultivation medium, for the various tested designs.

Access to carbon dioxide and water of suitable quality is important. The algae culture and its transformation should both take place at the same site. The facility is assumed to be established on a previously shrub land and is modeled as an industrial area with vegetation.

### Co-product consideration in the assessment

If more than one product is delivered from the system processes, all system flows must be divided proportionally to the energy content of the products, to the mass or to the market value. This division is called allocation. Another approach consists in substitution, which takes into account all products that can be replaced by the co-products; the system, therefore, receives credits for having cut down on the use of the initial product. This co-product management choice is fundamental in LCA and it can lead to completely different results [20]. Several co-products can be generated in the system during three steps: (i) oil extraction, (ii) transesterification and (iii) photovoltaic shading. The oil extraction process produces high value lipids (algal oil) and residual dry biomass (oilcake). Transesterification yields glycerine as a co-product while photovoltaic panels obviously produce electricity.

The impacts of co-products are based on an allocation approach according to their energy content [21], which is measured by their lower heating values (LHV). The co-products include surplus electricity, extraction residue (oilcake) and glycerine. Oilcake and glycerine have an energetic content (Table 1) and can be valorised as a source of energy, animal feed for oilcake and as heat source for glycerine [9]. Crude oil and oil cake differ in their carbon and energetic content, similarly to glycerine and biodiesel.

**Table 1:** Lower heating value (LHV) for co-products

Compound	Heating value (MJ/kg)	Refs.
Biodiesel	37.2	[9]
Algal oil	38.3	[3]
Oil cake	0.77^a^	[9]
Glycerine	18.1	[9]

^a^Composed by 95% water, 5% biomass (content around 70% carbohydrates and 30% protein), LHV based on composition

A three-stage allocation scheme is carried out: first, the impacts on electricity production from a photovoltaic system (Subsystem-5) to electricity injected into the facility and exported electricity (surplus electricity); second, the impacts incurred due to the production of oilcake and algae oil in the oil extraction subsystem (Subsystem-3) and third the apportioned impacts of glycerine production in the oil conversion subsystem (Subsystem-4). Table 2 presents the average annual allocations for different photovoltaic coverture ratios and consumption/production of electricity (see seasonal variations in the Additional file 1: File S4).

**Table 2:** Allocation factors used for biodiesel and co-products

	Percentage of coverture of photovoltaic panels									
	0%	10%	20%	30%	40%	50%	60%	70%	80%	90%
Allocation S5										
Electricity from PV panels into facility	0	84	55	36	26	20	17	14	11	9
Electricity exported (surplus)	0	16	45	64	74	80	83	86	89	91
Allocation S3										
Algal oil	65	65	64	64	64	63	63	63	63	63
Oilcake	35	35	36	36	36	37	37	37	37	37
Allocation S4										
Biodiesel	91	91	91	91	91	91	91	91	91	91
Glycerine	9	9	9	9	9	9	9	9	9	9

To study the sensitivity to the allocation method, a substitution computation is also carried out. Produced oilcake can be employed as animal feed in the same manner as soymeal can be used as a co-product from biodiesel. The protein content of soymeal is 48% [22], while it is around 30% in oilcake. Thus, 1 kg oilcake from algae replaces 0.6 kg of soybean for animal feed. The credits for not having to produce 0.6 kg soymeal for every kg algae oilcake produced are subtracted from the total upstream processes and emissions associated with the algal biodiesel production. Algal oilcake co-product replaces the soymeal production from a soybean crude oil production plant located in USA. Glycerine and surplus electricity co-products are, respectively, assumed to replace petroleum glycerine from an epichlorohydrin European plant and electricity production from a European mix, respectively.

### Microalgae specification

The analysis considers *Chlorococcum* sp. and *Desmodesmus* sp, since both species can achieve efficient trade-off between growth rate, lipid accumulation and ease of cultivation [23, 24]. Data are not consistent enough in the literature to accurately describe the variations in lipid profiles due to seasonal light and temperature variations. As a consequence, a constant TAG rate for each species is assumed according to nitrogen starvation conditions [25]. Additional file 1: File S5 provides general information on the biomass as well as compositional details. The analysis considers a 47% and 53.8% lipid content (of dry basis content biomass), for *Chlorococcum* sp. and *Desmodesmus* sp., respectively.

### Cultivation

Microalgae cultivation in a module consists of 5 raceways of 8348 m^2^ (2504.5 m^3^ total volume) mixed with a paddlewheel (more information in Additional file 1: File S1, Sections 1.2 and 1.3). The 5 raceways are grouped into 1 greenhouse; each greenhouse contains feed and harvest pipes between individual raceways and common headers, with the harvest lines drawn off raceways controlled by slide gates and valves and delivered to primary de-watering (in-ground gravity settlers). Paddlewheel mixing is considered in each raceway, which may be viewed as a standard basis for commercial scale facilities [26]. The inoculum generally represents around 10% of the operating volume of the raceway. The inoculum grows in the same medium as the production raceway (see more information in Additional file 1: File S1, Section 1.4). It is produced after an exponential phase prior to inoculation, within a small-sized raceway [27].

The process begins with algal biomass growth and harvesting. Biomass is harvested at a seasonally variable culture density first through a primary settler. The plumbing is often neglected in LCA studies. But it is a critical factor as it covers a large land footprint. Each pipeline is equipped with a valve for opening or closing the circulation of water, nutrients and/or inoculum in each raceway and inoculum pond. The piping and pumping systems involve five independent pipelines, detailed in the Additional file 1: File S2, Section 2.1.

The residence time is 10 days and harvesting is performed once a day for each raceway, representing 10% of the total volume (volume extracted by raceway is 218.4 m^3^ day^−1^) [1]. The raceway is fed with fresh medium at a specified flow rate. The feed point is typically located just before the paddlewheel. During feeding, the algal culture is either withdrawn or harvested from the raceway at a rate equal to the feed flow rate. Feeding and harvesting only occur during daylight and stop at night; otherwise the biomass could flush out the raceway overnight.

CO_2_ is supplied from a nearby fossil fuel power plant by direct injection of flue gas. Distribution is ensured thanks to a blower system, under moderate pressure using sufficiently thick HDPE pipes. Carbon requirements depend on biomass growth rate and concentration. The efficiency of the microalgae inorganic carbon uptake was assumed to be 75% [28], while the percentage of C in the biomass can vary according to the microalgae species (see Additional file 1: File S6, Section 6.2).

In addition to carbon dioxide, algal growth requires nitrogen (N) and phosphorous (P) as principal nutrients [29]. Nutrient requirements for the inoculum ponds and raceways are assumed to be met using diammonium phosphate (DAP, 18% N, 20.2% P) for phosphorous requirements, and ammonium nitrate (NH_4_NO_3_, 35% N) for nitrogen requirements at 20% w/w each. Percentages of N and P in biomass vary depending on the species of microalgae. In the case of N, a fraction of the element is also provided by DAP.

The fertilizer requirements in the inoculum ponds and raceways were calculated according to the species. For *Chlorococcum* sp., the nitrogen and phosphorous fertilizers are 0.0093 kg NH_4_NO_3_/kg algae biomass DW (0.026 kg N/kg algae biomass dry weight) and 0.0030 kg DAP/kg algae biomass DW (0.0053 kg P/kg algae biomass dry weight). For *Desmodesmus* sp. 0.0066 kg NH_4_NO_3_/kg algae biomass DW (0.018 kg N/kg algae biomass dry weight) was assumed and 0.0022 kg DAP/kg algae biomass DW (0.0038 kg P/kg algae biomass dry weight). These nitrogen requirements for *Chlorococcum* sp. and *Desmodesmus* sp., respectively, are similar to those reported by Collet et al. [9] for biodiesel production using *Nannochloropsis oculata* at nitrogen starvation (0.04 kg N/kg algae biomass dry weight). The areal fertilizer requirements in the raceways fluctuate according to the biomass productivity and, thus, to the season (detailed in Additional file 1: File S6, Section 6.1).

Whatever the location, the freshwater supply is insufficient to support any substantial scale production of algal fuels. The supply in brackish water is also relatively limited. Therefore, the use of seawater and marine algae would be a convenient option for producing algal fuels. Unfortunately, the use of seawater for algae culture does not totally eliminate the need for freshwater. Freshwater is still necessary for compensating evaporative losses and the consequent increase in culture salinity. Evaporative loss depends on the local climatic conditions, particularly on the irradiance levels, air temperature, wind velocity and absolute humidity [8]. Water is transported to the facility by pipeline from a nearby local marine water resource, while freshwater comes from outside of the facility boundaries. The transport of water used in the facility has been ignored in the study. Seawater is used in the cultivation and inoculum ponds, while freshwater is used for fertilizer dilution and for compensating water losses (mainly via pond evaporation). The blowdown volume was assumed to be equal to the water requirement. For inoculum ponds, there is no blowdown; however, dilution water in the fertilizer varies according to biomass productivity, while the evaporation volume is seasonally variable (see Additional file 1: File S6, Section 6.1).

### Pond emissions

The volatile compounds emitted by raceways and inoculum ponds are CO_2_, N_2_O and NH_3_. These emissions highly depend on operating conditions, such as dissolved oxygen concentration, pH, mixing rate, gas transfer coefficient, and nitrate concentrations, etc. [9]. Further experimental data are required to provide reliable emission factors. Nevertheless, due to lack of information, an average loss emission for each compound was inferred. These are correlated with other LCA studies [9].

Raceways have low CO_2_ injection efficiency, resulting in re-emission of a large fraction of flue gas. A 25% emission of injected CO_2_ was considered (250 g CO_2_ kg^−1^ CO_2_ injected). Nitrogen emissions (N_2_O and NH_3_) to the environment have been scarcely taken into account in the literature, even though these emissions present harmful effects (causing, among others, acidification, eutrophication and global warming). Indeed, N_2_O is a greenhouse gas with a much higher GWP (Global Warming Potential) than CO_2_ (298 kg CO_2eq_·kg^−1^ at a temporal horizon of 100 years). Especially during nighttime anoxic conditions, microalgae cultures have proved to generate both direct and indirect N_2_O emissions. Direct N_2_O emissions are related to the denitrification process, which reduces nitrate (NO_3_^−^) to nitrogen gas through a multistep process, with N_2_O as an intermediate product [30]. Complete denitrification involves the production and consumption of N_2_O which can be partially released into the atmosphere. N_2_O emissions represent 0.003% of the nitrogen fertilizer applied to a fully oxic culture (raceway case) and 0.4% for a microalgae culture that is anoxic during dark periods (photobioreactor case) [30]. In the present study, a 0.003% emission (0.0298 g N_2_O kg^−1^ N) was considered.

Indirect N_2_O emissions are the long-term fate of nitrogen fertilizers [31]. Indeed, by providing a substrate for microbial nitrification and denitrification after application in the soil, fertilizers indirectly generate N_2_O which then volatilizes [31]. In the present study, an emission of 1.6 g N_2_O kg^−1^ N [31] and 120 g NH_3_ kg^−1^ N was considered [9].

### Algae harvesting

Harvesting refers to the removal of algal biomass from the pond, as well as, occasionally, to the primary concentration step. Dewatering is a secondary concentration step [26]. As algal biomass dewatering technologies are still under investigation and development, the best strategy is still difficult to assess. The present model is based on the technology analyzed by NREL [26], offering an advantageous trade-off between dewatering performance (power demand, retention efficiency, etc.) and cost (capital and operating costs). Furthermore, this process avoids the addition of chemicals (i.e., flocculants or metal ions), thus maintaining biomass purity for downstream flexibility.

Biomass is harvested from the ponds and concentrated through three dewatering steps comprising gravity settlers, membranes and centrifugation to reach a final concentration of 200 g L^−1^ (more information in Additional file 1: File S7, Section 7.1). Table 3 summarizes the parameters of the selected technologies.

**Table 3:** Various parameters considered for study

Unit process	Assumptions	Refs.
Algae cultivation Algae growth	Algae strains: *Chlorococcum* sp. and *Desmodesmus F2* sp: 47% and 53.8% lipid content for *Chlorococcum* sp. and *Desmodesmus* sp. Velocity culture: 0.3 m s^−1^ for raceways and 0.25 m s^−1^ for inoculum ponds HRT: 10 days. Raceways: 110 units of 310 m long × 30 m weight x 0.3 m height (2184.3 m^3^ volume medium). Inoculum ponds: 40 units of raceways of 160 m long × 15 m weight × 0.35 m height (656 m^3^ volume medium) Facility: 145 ha area. Operating time facility: 330 days year^−1^ (90%) Paddlewheels: 0.11 W/m^2^, time functioning: 12 h day^−1^. One unit per raceways and inoculum pond Blower system: 22.2 Wh kg^−1^ CO_2_, time functioning: 12 h day^−1^. One unit per raceways and inoculum pond. 14% v/v CO_2_ concentration in flue gas Water loss (evaporation): daily variable (ranging between 0.01 and 0.34 cm day^−1^) Inoculum input pumping system: power: 10 kW, 22 units, time functioning: 0.8 h h day^−1^. Electricity consumption: around 0.07 kWh m^−3^ Nutrients/water loss pumping system: 24 units (22 for raceways and 2 for inoculum ponds), time functioning: 12 h day^−1^. Electricity consumption: negligible	[23, 24, 32–34]
Algae harvesting (dewatering)	Settlers ponds: 22 units, energy demand: negligible, efficiency: 90%, outlet concentration: 10 g/L. Capacity: 364.1 m^3^. Residence time: 4 h Membranes: 22 units, power: 2 kW, energy demand (variable): 0.03 to 0.2 kWh m^−3^, efficiency: 99.5%, outlet concentration: 130 g/L. Capacity: 2.3 m^3^ h^−1^, time functioning: 12 h day^−1^ Centrifuges: 22 units, power: 6 kW, energy demand (variable): 0.9 to 5.05 kWh m^−3^, efficiency: 97%, outlet concentration: 200 g/L. time functioning: 12 h day^−1^ Overall harvesting process: 20% wt outlet concentration. Efficiency: 86.9%. Percentage of water volume reduced: 99.9% Harvesting pumping system: 22 units, power: 7.7 kW, energy demand: 0.08 kWh m^−3^, time functioning: 12 h/day Recirculation pumping system: 22 units, power: 7.7 kW, energy demand: 0.08 kWh m^−3^, time functioning: 12 h/day	[26, 34]
Oil extraction	Sonication: 2 units, power: 16 kW, energy demand: 0.013 kWh kg^−1^ algae-DW, capacity: 12 m^3^ h^−1^, time functioning (variable): 1.5 to 8.8 h/day Static mixer: 1 unit, power: 6 kW, energy demand: negligible, efficiency lipid extraction: 90%, capacity: 12 m^3^ h^−1^, time functioning: 1.5 to 8.8 h/day. Hexane input: 10:1 mass ratio, 0.05% hexane losses Biomass solvent separator: 1 unit, power: 6 kW, energy demand: 0.005 kWh kg^−1^ algae-DW, Efficiency: 99.9%. Capacity: 5.7 m^3^ h^−1^ time functioning (variable): 3 to 19 h/day Distillation column: 2 units, energy demand (variable): 0.09 to 0.55 kWh kg^−1^ oil, capacity: 15.2 m^3^ h^−1^ time functioning (variable): 2.7 to 16 h day^−1^	[28]
Oil conversion	Transesterification reactor: 1 unit, power: 15 kW, energy demand: 0.03 kWh kg^−1^ biodiesel, time functioning (variable): 2.7 to 16 h/day. Chemical consumption: methanol 1.1 kg kg^−1^ biodiesel, Sodium methoxide 0.11 kg kg^−1^ biodiesel, HCl 0.014 kg kg^−1^ biodiesel, NaOH 0.008 kg kg^−1^ biodiesel, natural gas 0.063 L kg^−1^ biodiesel	[35]

### Algae transformation

The extraction step involves the addition of hexane as solvent, followed by a recovery phase where hexane is recycled. The current model is based on the oil extraction processes documented by Rogers and Rosemberg [28] for a biodiesel plant production at commercial scale. Yield extraction, hexane volume and associated heat and electricity consumptions have been adapted to match the data of this analysis (more information in Additional file 1: File S7, Section 7.2).

### Combustion emissions

The emissions associated with combustion are assumed to be equivalent to rapeseed-based biodiesel emissions. The emission factors refer to a EURO-3 middle-sized vehicle. They are extracted from the Ecoinvent database [36], assuming a fuel consumption of 0.42 km per MJ of biodiesel. Conventional diesel engines are considered to have the same consumption (see combustion emissions factors in Additional file 1: File S8).

### Photovoltaic system

The core of a photovoltaic system is the solar cells converting light energy into electricity. Electricity then generates an electromotive force when the radiation reaches a semiconductor plate presenting a potential gap [37]. Copper indium gallium diselenide (Cu(In, Ga)Se_2_, CIGS) is a mixed alloy of copper indium diselenide (CuInSe_2_, CIS) and copper gallium diselenide (CuGaSe_2_, CGS) semiconductors [38]. In comparison to traditional silicon-based technologies, CIGS is appealing because of its competitive cell efficiency and performance in diverse environments [39]. Furthermore, although current efficiency for CIGS cells averages 14%, technological advancements presently contribute to the improvement of cell efficiency with records up to 23% [39], potentially rendering CIGS increasingly competitive compared with current silicone-based cells. This study considers a conservative efficiency of 15% and a 30-year lifespan for a 1 m^2^ area module. The CIGS technology data from Wurth Solar (Germany) were used [40], considering mass and energy flows over the whole production process starting from material extraction to the final panel assemblage, use and end of life. Different layers of CIGS thin film cells are necessary. The required sequence layers are deposited in a number of subsequent production steps. The active layer consists of a specific copper–indium–selenium configuration deposited by a vaporization process directly over a large area of window glass (substrate material). It is usually airtight sealed with a second glass plate. The modules have a size of 1.2 m by 0.6 m and a weight of 12.6 kg [40]. In Additional file 1: File S9, the monthly electricity production is plotted as a function of the percentage coverture of photovoltaic. These data have been obtained from the Sophia Antipolis meteorological database (France).

### Energy assessment

A cradle-to-gate life cycle energy analysis was performed, including the production of raw materials and the production process of biodiesel. The fossil energy ratio (FER) and net energy ratio (NER) were estimated according to the input and output energy for 1 MJ of biodiesel. There are no allocations in energy balance. FER is defined as

$${\text{FER}} = \frac{{{\text{Renewable}}\;{\text{energy}}\;{\text{output}}}}{{{\text{Fossil}}\;{\text{energy}}\;{\text{input}}}} = \frac{{{\text{LHV}}}}{{{\text{CED}}}}$$

The FER only included fossil (non-renewable) energy in the denominator. NER includes total energy input in the denominator, including renewable sources of energy, such as wind and solar. NER, rather than FER, is used as an indicator of energy efficiency [41].

LHV (low heating value) is the life cycle energy output (MJ), determined using the following equation:

$${\text{LHV}} = {\text{EP}}_{\text{biodiesel}} + {\text{EP}}_{\text{oilcake}} + {\text{EP}}_{\text{glycerin}} + {\text{EP}}_{\text{surplus electricity}}$$

EP represents the energy for each co-product (MJ), each being defined as

$${\text{EP}}_{\text{biodiesel}} = 1 \left( {\text{Functional unit}} \right)$$

$${\text{EP}}_{{{\text{glycerine}}}} = {\text{Mass}}\;{\text{glycerine}}\;\left( {\frac{{{\text{kg}}}}{{{\text{MJ}}\;{\text{biodiesel}}}}} \right) \cdot {\text{LHV}}_{{{\text{glycerine}}}} \left( {\frac{{{\text{MJ}}}}{{{\text{kg}}}}} \right)$$

$${\text{EP}}_{\text{oilcake}} = \mathop \sum \limits_{i} P_{{{\text{oilcake}}, n}} \cdot {\text{LHV}}_{n}$$

$${\text{EP}}_{\text{surplus electricity}} = {\text{Surplus elecrticity}} \left( {\text{exported}} \right) {\text{from photovoltaic panels}}\; ( {\text{MJ)}}$$

where $P_{{{\text{oilcake}}, n}}$ is the percentage of component *n* in the oilcake (%, e.g., carbohydrates, lipids, proteins, etc.) and ${\text{LHV}}_{n}$ is the lower heating value of component n (MJ/kg).

Cumulative energy demand (CED) represents the life cycle total energy consumption (in MJ):

$${\text{CED}} = \mathop \sum \limits_{i} \mathop \sum \limits_{j} {\text{EE}}_{i,j } \cdot {\text{PE}}_{j} + \mathop \sum \limits_{i} \mathop \sum \limits_{n} M_{i,n } \cdot {\text{PE}}_{n}$$

where ${\text{EE}}_{i,j }$ is the *j*th process energy consumption during stage *i* (MJ), ${\text{PE}}_{j}$ is the total energy use for process *j* production (MJ/MJ) (renewable and non-renewable for NER and non-renewable for FER) $M_{i,n }$ is the *n*th material consumption during stage *i* (kg). ${\text{PE}}_{n}$ is the life cycle total (renewable and non-renewable for NER and non-renewable for FER) energy use for material *n* production (kg/MJ).Values of CED for material and energy used in the various processes are obtained from the CED method v1.09 (see Additional file 1: File S10).

### Environmental assessment

The standard framework of Life Cycle Assessment (LCA) described by ISO 14040:2006 was selected to assess the ecological burdens and energy balance. An attributional LCA is used in the analysis, which considers only physical relationships between each process, different from a consequential LCA where economic relations are also assessed [9]. LCA software SimaPro v8.3 [18] was used for modeling the data, using the characterization factors from the midpoint (H) ReCiPe 2008 method v1.3 [44]. Full LCI data source are available as supplemental information (Additional file 1: File S10) [42]. The following impact categories were considered: climate change (CC), ozone depletion (OD), human toxicity (HT), photochemical oxidation formation (POF), particulate matter formation (PMF), terrestrial acidification (TA), freshwater eutrophication (FE), marine eutrophication (ME), terrestrial ecotoxicity (TET), freshwater ecotoxicity (FET), marine ecotoxicity (MET), ionising radiation (IR), natural land transformation (NLT), urban land occupation (Urban LO), agricultural land occupation (Agri LO), water depletion (WD), metal depletion (MD) and fossil depletion (FD). The endpoint (H) ReCiPe 2008 method is also used to assess the system at a more aggregated level through the three areas of protection (AoP): Human Health, Ecosystems and Resources.

### Mathematical model predicting monthly productivities

The model predicting temperature in the raceway ponds was based on the heat balance presented by Béchet et al. [43], which was initially developed for an open raceway pond and validated to large scales [49]. In the Béchet model, a total of eight heat fluxes were considered:

- Solar radiation;
- Long-wave air radiation;
- Long-wave pond radiation; Convection with the air flowing at the pond top surface;
- Evaporation from the pond surface;
- Conduction with the soil beneath the pond;
- Heat flux associated with the water inflow; and
- Heat flux associated with rain.

The model developed by Béchet et al. [43] still needs to be significantly modified as the presence of the greenhouse significantly impacts the expression of most of these heat fluxes:

- Solar and air radiation is partly shaded by the greenhouse;
- Pond radiation is partly reflected back toward the pond by the greenhouse.
- Convection and evaporation are “natural” in a greenhouse as there is no wind to force these transfer mechanisms;
- Rain heat flux is obviously inexistent in a closed greenhouse;
- Conduction and inflow heat fluxes were, however, expressed similarly to the case of an open pond.

The greenhouse is assumed to be of rectangular shape and condensation on the greenhouse walls was neglected. All opaque surfaces were considered as diffuse gray, except for the greenhouse walls that were considered as partly transparent. For the reflected radiative heat fluxes, only single reflection was accounted for. Finally, the temperature and relative humidity in the greenhouse are considered homogenous.

The air temperatures inside and outside the greenhouse are different. As the air temperature above the pond impacts both evaporation and convection at the pond surface, the air temperature inside the greenhouse needs to be assessed in parallel to the pond temperature. A heat balance on the air in the greenhouse was, therefore, computed to determine the air temperature at each time step of the simulation. The greenhouse walls emit inward long-wave radiation, a fraction of each being absorbed by the pond. The temperature of the greenhouse walls was, therefore, evaluated at each simulation time step through a heat balance on the greenhouse walls.

This heat balance is relatively complex due to the high number of radiative interactions between the greenhouse and its surrounding environment. Indeed, the pond, the ground inside the greenhouse and the ground outside the greenhouse emit long-wave radiations that are partly absorbed by the greenhouse. The long-wave radiation emitted by a gray body depends on its temperature and, as a result, the temperatures of the inside and outside ground surfaces were determined simultaneously through two additional heat balances. It is not straightforward to determine the ground surface temperature as it depends on the conductive properties of the soil. Indeed, ground surface temperature decreases when the ability of the soil to conduct heat in deeper ground layers increases. This conductive heat flux is a function of the soil thermal properties but also of the temperature gradient within the soil. Therefore, to determine the internal and external ground surface temperatures, the temperature profiles in the soil first need to be assessed. In summary, to determine the pond temperature in the greenhouse, a total of five different heat balances were solved simultaneously during the simulations.

## Results and discussion

Dynamic seasonal growth modeling is an important step that critically impacts results. Monthly variations in the life cycle inventory depend on the monthly biomass productivity, which in turn affects lipid and biodiesel productivity (see Additional file 1: File S11). Large differences in assumptions on the productivity potential have directly contributed to the large variance in LCA results from various studies [44]. The high lipid yields reported in the literature are typically the result of speculation for future productivity potentials, based on the linear scaling of laboratory data [44]. This highlights the importance in developing realistic dynamic productivity models based on experimentally validated biological models integrated with local and seasonal meteorological data [45]. Table 4 shows the evolution of the microalgae biomass productivity, respectively, for each species, obtained from the mathematical model based on Mediterranean conditions (Sophia Antipolis, France). According to simulation results, *Chlorococcum* sp. was chosen for the colder months and *Desmodesmus* sp. for the warmer months, depending on the coverture fraction of photovoltaic panels. When the coverture is greater than 60%, only *Chlorococcum* sp. was chosen because *Desmodesmus* sp. had a very low productivity at low light (< 1 g·m^−2^ day^−1^).

**Table 4:** Monthly biomass productivity (g m^−2^ day^−1^)

% PV panel	January	February	March	April	May	June	July	August	September	October	November	December
0	9.79	16.52	26.74	20.59	*19.69*	*22.34*	*19.40*	*20.98*	*15.19*	18.49	12.45	9.12
10	8.88	15.42	24.79	26.20	*18.29*	*21.14*	*18.40*	*19.50*	*14.18*	17.18	11.65	8.26
20	7.93	14.08	22.65	26.33	15.94	*19.73*	*17.23*	*17.87*	18.23	15.67	10.81	7.38
30	6.83	12.40	19.99	25.11	26.35	*17.94*	*15.76*	*16.26*	18.01	13.96	9.58	6.36
40	5.84	10.80	17.46	23.08	26.14	*16.16*	*14.29*	18.58	17.66	12.37	8.40	5.44
50	4.81	9.12	14.86	20.42	24.21	18.35	*12.62*	17.25	18.88	10.69	7.16	4.51
60	3.74	7.38	15.78	17.31	21.10	20.76	15.61	21.19	16.21	9.41	5.86	3.52
70	2.59	5.52	12.53	14.73	17.21	19.02	15.77	17.81	13.25	7.94	4.50	2.54
80	1.32	1.85	8.51	10.78	12.20	14.29	12.21	12.10	9.29	5.17	2.80	1.24
90	1.00	1.00	3.04	4.85	7.48	8.12	6.99	5.26	4.97	2.41	1.02	1.05

*Chlorococcum* and *Desmodesmus* sp. (italics text)

Ten scenarios were considered: absence of photovoltaic panel (0% coverture), and greenhouse roof coverage from 10 to 90%. 100% coverture was not considered since it would hinder any biological productivity.

### Energy flows

**Fig. 2:**
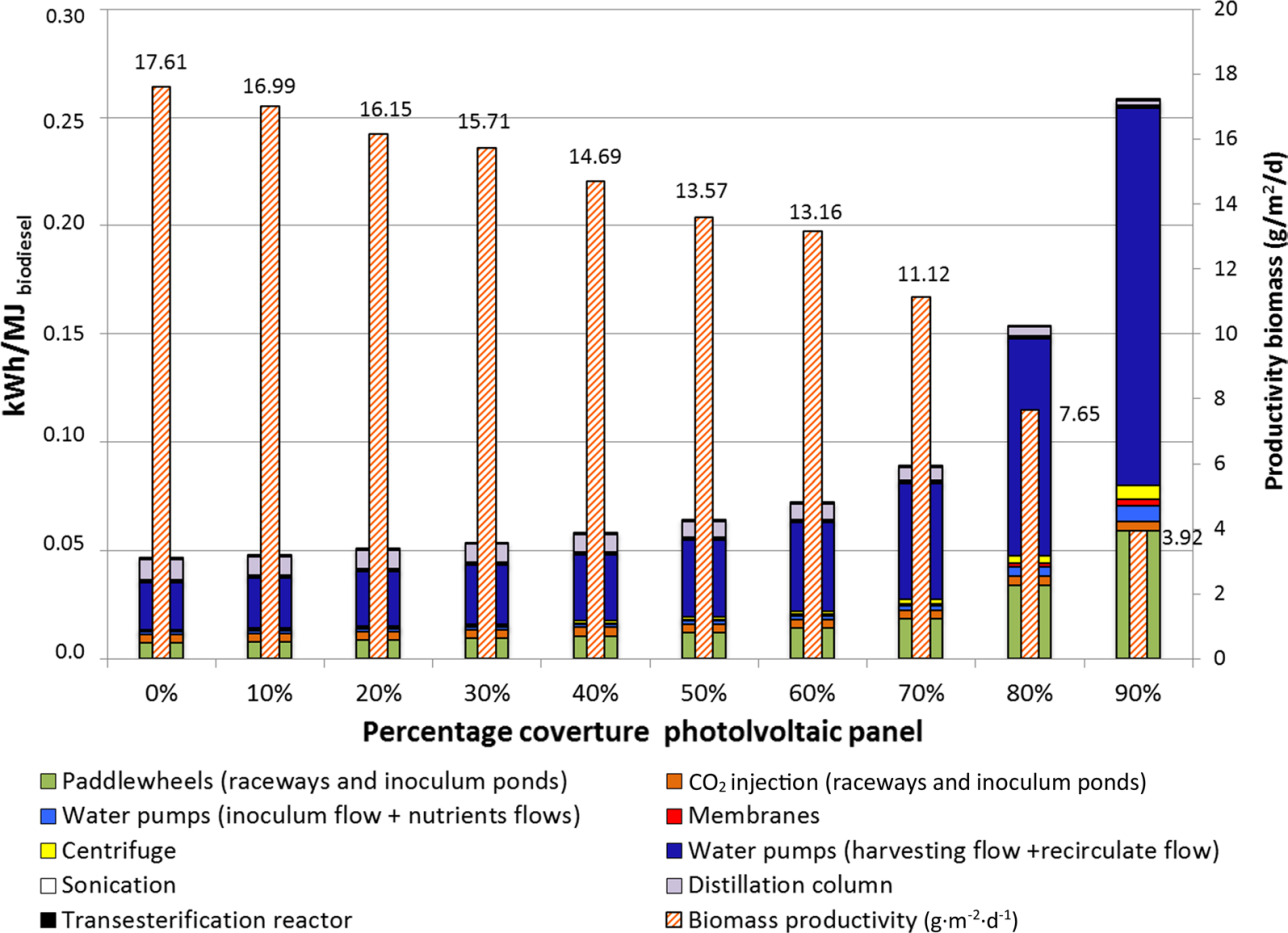
Annual average net electricity input and biomass productivity depending on PV coverage. Monthly biomass productivity average values are indicated above bars

The use of energy for each step of the process was derived from algal productivity, dewatering, oil extraction and transesterification (see Table 3). Figure 2 illustrates the energy requirements in the different case studies. The main energy requirement results from water pumps used for harvesting and recirculating flows from dewatering processes, followed by paddlewheel engines (more details in Additional file 1: File S1, Section 1.3; Additional file 1: File S2, Section 2.2 and Additional file 1: File S3, Sections 3.2 and 3.3). The biomass productivity decreases when the coverture fraction of photovoltaic panels increases at a variation rate below 5% and between 0 and 30% photovoltaic coverture; however, at 70% photovoltaic coverture this variation rate increases to more than 15% (reaching almost 50% less biomass productivity at 90% with an 80% photovoltaic coverture).

The NER and FER results are depicted in Additional file 1: File S12. Allocation issues do not affect this evaluation, i.e., all production processes are considered as a whole. The total set of products represents an amount of energy (in terms of LHV) ranging from 1.70 MJ_LHV_ without PV up to 9.82 MJ_LHV_ with 90% photovoltaic coverture. The total energy investment, CED (renewable + non-renewable energy), ranges from 0.90 (without PV) up to 9.93 for 90% PV. This implies a favorable NER over the whole year, i.e., even in the absence of photovoltaic panels: 1.99 and FER: 2.92. Without PV panels, the electricity should be supplied by the European electricity matrix. In comparison with other similar LCA studies on algal biodiesel, the NER for biodiesel from microalgae using fossil fuel electricity sources is usually slightly greater than 1 [3, 46, 47], although some cases can be lower than 1, as reported by Lardon, Hélias [3] and Yang, Xiang [48].

With photovoltaic panels, the highest NER (larger than 5.0) is obtained during the hottest months (April to September) (see Additional file 1: File S13). Indeed, during the summer period, the electricity production is higher (large electricity production in comparison to the facility requirements). However, despite optimal energetic performance resulting from the use of photovoltaic panels, the relevance of renewable biofuels rather becomes a matter of producing storable and renewable energy. The production of biodiesel from microalgae is an efficient way to store a fraction of renewable energy. The optimal percentage of photovoltaic panels depends on the month: i.e., during the colder months (October to March), the optimal coverture is 10%, while for warmer months (April to September) the optimum is 20% coverture.

**Fig. 3:**
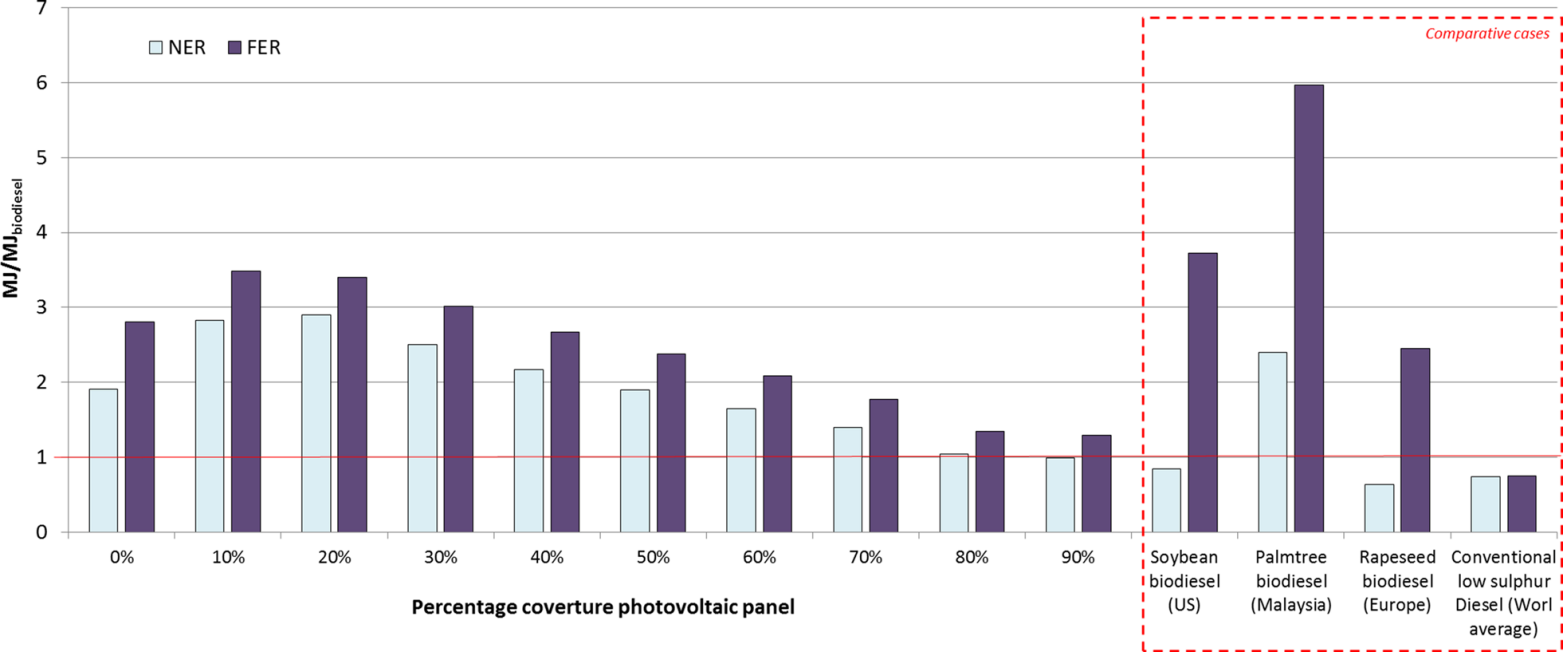
NER and FER comparison *pond*-*to*-*wheels* life cycle microalgae-based biodiesel with first-generation biodiesel and conventional diesel

Figure 3 compares NER and FER along the different scenarios, with first-generation biodiesel and conventional diesel. The reference cases are obtained from the Ecoinvent database for biodiesel [36] and conventional fossil diesel [49], complying with similar limits for the system and for the allocation of this study. The biodiesel reference scenarios are soybean diesel (US), palm tree diesel (Malaysia) and rapeseed diesel (European average) (more details about comparative cases can be found in Additional file 1: File S14). A 10% and 20% coverture fraction of photovoltaic panels are the most optimal configurations that obtain highest FER and NER, respectively. The presence of 10% and 20% photovoltaic panel yields a higher NER than first generation and fossil diesel. However, FER presents better results in the cases of soybean and palm tree biodiesel, despite the use of photovoltaic panels.

### Environmental impacts

First-generation biodiesels and fossil diesel are compared in Additional file 1: File S15, with endpoint characterization results for the combustion of 1 MJ of biodiesel in a medium-sized car for various fractions of photovoltaic panel coverture. The lowest impact is obtained from a 50% coverture, with equivalent performances from 30% to 60%. The main subsystem contributors are the culture, followed by the photovoltaic subsystem, in the case of human health and resources, or combustion in the case of the ecosystem category. Biodiesel from microalgae has the following characteristics:

- Algal biofuel leads to significant reductions in the Human Health and Ecosystem categories compared to other biodiesels, but is still higher than conventional diesel.
- Significant reductions in the Resources impact category are obtained relative to conventional diesel; however, the impact is higher than for soybean diesel and palm tree diesel.

Additional file 1: File S16 presents the contribution of each process to climate change, accounting for production of electricity using PV panels. Results for midpoint categories are detailed in the Additional file 1: File S17. The data in Table 5 make it possible to compare the impact results of algae biodiesel to those obtained by fossil diesel and first-generation biodiesels. These overall results on comparisons with other scenarios are coherent with the study by Collet, Lardon [9]. It is important to note that some categories increase for a large coverage of photovoltaic panels (> 80% coverture), such as POF, PMF, TA, ME, or FET. However, without photovoltaic panels some impacts are different, such as IR, mainly due to the electricity requirement or MD due to the production of photovoltaic panels, respectively.

**Table 5:** Comparison of LCA results between algae biodiesel and conventional or first-generation biodiesels

Impact category	Algae biodiesel in comparison to:			
	Conventional fossil Diesel	Palmtree Biodiesel	Rapeseed Biodiesel	Soybean Biodiesel
Ozone depletion	**−**	+	−	∓
Human toxicity	+	+	∓	+
Photochemical oxidation formation	−	∓	−	∓
Particulate matter formation	∓	∓	∓	+
Terrestrial Acidification	∓	∓	−	+
Freshwater eutrophication	+	+	∓	∓
Marine eutrophication	∓	−	−	−
Ionizing radiation	∓	+	∓	∓
Water depletion	+	+	+	+
Metal resources depletion	+	+	∓	+
Fossil resources depletion	−	+	∓	+
Natural land transformation	**−**	−	−	−
Agricultural land occupation	+	−	−	−
Urban land occupation	∓	∓	−	∓
Terrestrial ecotoxicity	+	−	−	−
Freshwater ecotoxicity	+	∓	−	+
Marine ecotoxicity	+	+	∓	+

**−** Impact reduction for algae biodiesel; + impact increase for algae biodiesel

∓ Impact reduction or increase for algae biodiesel, depending on the percentage of photovoltaic panel coverture

The overall results highlight the contribution of the culture, infrastructure production and use. This is coherent with results from contribution analyses in other studies [3, 9]. Culture (Subsystem-1) is the main contribution for most of the assessed impacts (CC, PMF, TET, TA, OD, FD, HT, Nat LO, Agri LO and Urban LO). For the remaining categories, culture is classified as a second contributor, preceded by the photovoltaic system (Subsystem-5) in the case of FET, MET, IR, FE and MD, or combustion (Subsystem-6) in POF and ME.

The infrastructure in the culture (Subsystem-1) has a significant effect in terms of CC, PMF, OD, FD, HT, Nat LO, Agri LO and Urban LO, due to the production of materials (mainly steel, PVC, HDPE, aluminum and concrete) used in the greenhouse, and to machinery and pipe productions. In addition, pond emissions from culture mainly contribute to TA and TET through volatilized ammonium and N_2_O. Although nitrogen fertilizer requirements are reduced (the culture system works under nitrogen-limiting conditions to improve the lipid contents in microalgae), nitrogen-based fertilizer production remains the main contributor in these categories.

The different metals and energy used to build the CIGS system highly contribute to the impacts of the photovoltaic system (Subsystem-5). Silver used for screen manufacturing contributes to MD, CC, TA, PMF and HT. This is mainly due to the impacts generated by the extraction and processing of silver, including also its high requirement in fossil energy (which strongly contributes to IR). In addition, extraction and manufacturing of stainless silver (substrate) essentially impact OD, while water used for washing the substrate affects WD and eutrophication categories. Other metals, such as copper, indium, gallium and selenium used in the CIGS layer and cabling, contribute to eco-toxicity and eutrophication categories.

Combustion emissions mainly affect POF and ME; and in a lower extend to CC, PMF, TET and TA. The carbon burned during the biodiesel combustion is biogenic as it originates from photosynthetic fixation, i.e., zero greenhouse emissions in the form of CO_2_ are assumed. Hence, the environmental impacts are due to other compounds and/or fossil carbons that are related to the production of chemicals, such as methanol for esterification.

The electricity required for the transformation subsystems (dewatering, oil extraction and oil transformation) at low percentage of photovoltaic panel coverture has an important impact for most of the categories. Nevertheless, the presence of photovoltaic panels at a larger percentage of covertures turns out less important from an environmental impact aspect. It also becomes a secondary source of impact for some categories, such as OD, FD and Nat LO, mainly due to chemical production (used in the esterification) and transports. The considered processing system does not exist at industrial scales. Hence, this part of the analysis has the most uncertainties and can be subjected to errors in the calculation of energy consumption or waste production. Nevertheless, alternative choices have already been tested individually in different studies [26, 28, 35]. This represents a reasonable projection of the processes and avoids over-optimistic or unrealistic assumptions.

**Fig. 4:**
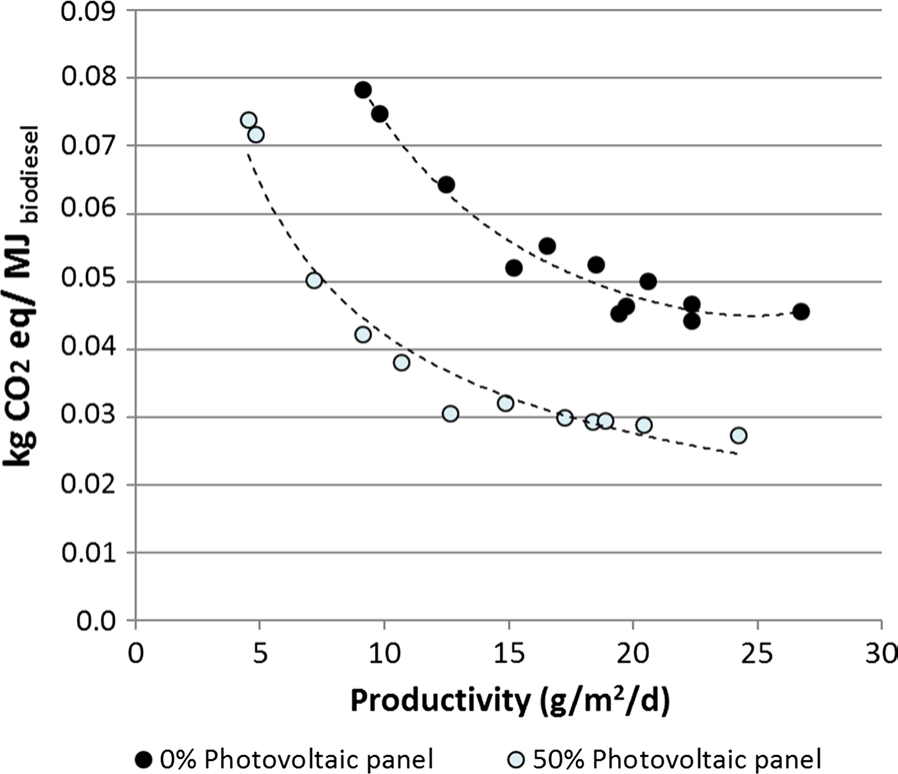
Climate change according to areal productivity and PV coverture

One of the main objectives of this study is to scale the expected gains on microalgae biodiesel production with respect to the reduction of GHG emissions, when a renewable energy source is considered. In comparison with the cultivation of microalgae without PV, the use of photovoltaic panels triggers a synergetic effect, acting both as a source of electricity and to reduce climate change impacts (Additional file 1: File S16). Similarly to endpoint category results, the scenario with a 50% PV coverture points to lower impacts on climate change. From a 0% to 80% coverture, climate change emissions are lower for algae diesel in comparison to biodiesel (except for soybean biodiesel) and diesel. A 90% PV coverture leads to highest values in climate change due to the numerous photovoltaic modules and to the strong decrease in biomass productivity. Additional file 1: File S18 comprises monthly GHG emissions for a 50% PV coverture. From April to September, values remain below 0.03 kg CO_2eq_·MJ _biodiesel_^−1^, while during the rest of the year, GHG emissions are higher, with values greater than 0.07 kg CO_2eq_ MJ _biodiesel_^−1^ in winter (December, January). The percentage of decrease depends on the quantity of electricity produced. The higher electricity production during the summer months contributes to the strongest decrease in GHG emissions (In the case of a 50% coverture, emissions reach about 40% less than for the case without PV panels). Nonetheless, the reduction in GHG emissions is lower in winter (November to February), varying between 4% and 24% (for a 50% PV coverture) compared to the nominal case excluding PV. Figure 4 illustrates the effect of biomass productivity on GHG emissions. The decrease in GHG emissions is directly connected to increasing microalgae productivity. Without photovoltaic panels, when the biomass productivities are higher than 20 g_biomass_ m^−2^ day^−1^, GHG emissions remain within the range of 0.05 to 0.045 kg CO_2eq_ MJ _biodiesel_^−1^. With a 50% PV coverture, the contribution to climate change emissions varies around 0.03 kg CO_2eq_ MJ _biodiesel_^−1^ when the productivity is higher than 12 g_biomass_ m^−2^ day^−1^.

### Reaching an optimal trade-off

In addition to trying to identify processes with limited energy requirements, the combination of biomass production with PV electricity represents an ideal opportunity to significantly reduce environmental impacts by almost 50% of GHG emissions. However, there is a clear trade-off between electricity and biomass production, as a larger PV coverture would limit microalgae production. This trade-off is associated with a series of optimal process designs and operating strategies that are correlated.

Higher biomass productivity related to higher biodiesel productivity could be achieved in the absence of PV panels. Adding photovoltaic panels can enhance productivity for the hottest months, but reduces biomass productivity on a yearly basis (each 10% PV coverage leads to a decrease of about 5% in the biomass productivity, but the decrease rate is higher for a PV coverage greater than 70%). However, at low PV coverage, consumption of electricity from the grid affects the energetic ratio (NER). A 10% coverage of PV increases NER by 48% (1.91 MJ/MJ for 0% PV and 2.83 MJ/MJ for 10 PV), with a peak value at 20% PV coverage (at PV coverage greater than 20%, NER decreases due to lower biomass productivity and higher energetic demands in the infrastructure construction). Thus, from an energetic point of view, the optimal configuration lies between 10% and 20% of PV coverage. Nevertheless, from a human health, ecosystem, resources and climate change point of view, the best option is 50% PV coverage. However, the difference between impact values obtained for 20% and 50% PV is negligible (difference of 7%; 0.044 kg CO_2eq_ MJ _biodiesel_^−1^ and 0.040 kg CO_2eq_ MJ _biodiesel_^−1^ for 20% and 50% PV coverage, respectively), while the NER is 48% higher for 20% PV than for 50% PV coverage. Hence, 20% coverage of photovoltaic panels can be considered as a sound and optimal energetic environmental configuration.

In addition, two high potential species have been studied with a monthly optimized strategy. As ventilation controls the greenhouse climate, medium temperatures are maintained close to the optimal growth temperature. The thermal properties depend on the PV coverage and, thus, the succession in cultivated species can vary. The trade-off that needs to be reached is constrained by the local climate and should, therefore, strongly depend on the location of the plant. Even though a 20% PV coverage has been defined as the best option from an energetic and environmental point of view, the complex and dynamical optimization problems still need to be revisited for any new climate conditions, while the solutions would depend on the targeted species, which must be chosen according to these light/temperature conditions. In a special case study, Barbera et al. [50] have shown that 30% of PV coverage was still beneficial from an economic point of view. Note that 20% PV coverage has been identified as an efficient economical trade-off for traditional agriculture under greenhouses [51, 52], supporting the idea that it is also relevant for more energy demanding cultures of microalgae.

The economic trade-off could also be estimated via a life cycle cost in addition to LCA. Then, economic allocation could be considered where impacts are allocated as a function of revenues, but this requires knowing the value of the products, which is obviously very uncertain in prospective scenarios, especially for microalgae whose market is still very immature. Indeed, beyond the economic value associated with their energy content, microalgae have a higher economic value associated with valuable co-products. PV panels reduce biomass productivity at a yearly scale and, thus, a trade-off at a lower PV coverage can be expected for the valorisation of the co-products when focus is put on economic aspects. The photovoltaic greenhouse has another advantage compared to classical raceways, since it lengthens the production season by modulating the greenhouse climate, hence favouring a better return on investment.

### Allocation method selection

The allocation methods, which are, in this case, based on energy, cover the co-products, the emissions as well as their impact on the functional unit. Allocation factors of co-products strongly reduce the impacts of biodiesel (see allocations factors in Table 2). Their values reflect each upstream chain phase benefit from all downstream co-products in the allocation process [53]. In this case, oil extraction (subsystem 3), oil conversion (subsystem 4) and photovoltaic covertures (subsystem 5) benefit from seed meals, glycerin and electricity, respectively. However, the energetic allocation does not highlight the actual use of co-products derived from the biodiesel production chain. The substitution method highlights the importance of co-product valorization, in which co-products are considered as amendments. The saved emissions, resulting from the substitution of conventional products by co-products, are reported with a negative value since they tend to reduce the impact.

Even though an energetic substitution method is accepted for biofuel sustainability certification, the results also need to be evaluated by a substitution method, while “estimates would change if co-products were accounted for using the substitution approach” [54]. To highlight the importance of considering co-products on the impact of a functional unit, the environmental performance of the substitution method was evaluated and compared with the results produced by the energetic allocation method (Additional file 1: File S20). It is noteworthy that when co-products are taken into account, the environmental balance is reversed and results are dramatically affected. 90% PV coverage is associated with lower environmental impacts on human health, ecosystems, resources and climate change categories. This is essentially related to the higher surplus electricity production, which reduces the electricity demand from the European electricity grid. Surplus electricity arises from the large percentage of photovoltaic panels, while electricity consumption is reduced within the facility (due to extremely low biomass productivity). Regrettably, the lower environmental impacts assessed with the substitution method, under conditions of negligible biomass productivity and high photovoltaic electricity, are not compatible with the production of microalgae biodiesel. The representation of a co-product by substitution also implies a modification of the addressed question. The allocation approach (using the energetic content as a criterion for partitioning) focuses the study toward the relevance of microalgae biodiesel as an alternative fuel. However, substitution answers a much broader issue. Co-product management practice ends up with a choice between fuel and electricity productions. Results point out that although electricity production is the main issue, it is misleading for the eco-design of an efficient alternative fuel production system.

It is crucial to manage co-products appropriately if the energy balance and environmental performance of the overall system are to be enhanced. Substantial energy is also stored as organic matter in the oilcake (obtained from oil extraction), and the energetic allocation assumes an energetic potential for the oilcake. This illustrates how complicated it can be to assess the energy balance and environmental impact in algal systems. Certain processes developed to extract this energy include anaerobic digestion and co-digestion, whose digestate can provide the necessary nutrients, thus reducing the incorporation of external fertilizers. Anaerobic digestion also contributes recovering a fraction of the energy content in oilcake [9] in the form of biogas. However, most of the studies dedicated to anaerobic digestion in microalgae point out that external energy is necessary to run the digester [55–57].

The sustainability-turn between both allocation methods, this highlights first the importance of considering the actual uses of co-products, and secondly how the consequences of substituting conventional products can strongly modify the sustainability assessment of biofuel. The oil yield and biomass productivity are, therefore, not the only parameters that must be taken into account for selecting a sustainable biodiesel production, since co-products also have a significant role. More details about substitution method results and comparison with rapeseed, palm trees, soybean and conventional diesel are described in the Additional file 1: File S14, Additional file 1: File S19 and Additional file 1: File S20.

### Improvement pathways

High production costs are the major limitation for the commercialization of algae-based biofuel. It is expected that the price of algal biofuels drops when the biomass and lipid productivity are improved [58]. More recent strategies to enhance biomass and lipid productivity in microalgae include genetic and metabolic engineering [59, 60], addition of phytohormones [61], and co-cultivation of microalgae with fungi [62], yeasts [63, 64] and bacteria [65]. By enhancing the performance of microalgae, which, nowadays, are still wild species, productivity should also increase. Bonnefond et al. [66] have proposed a promising strategy for improving algae efficiency with a lower sensitivity to temperature fluctuations. Their approach resulted in extending the thermal niche with an enhancement of the maximal growth rate and lipid content. In addition, the use of additional species all along the year could probably further improve the process.

This study focuses on classical raceway systems, even though more productive systems could be used, such as biofilm-based processes [67], which are likely to considerably reduce energy and harvesting and dewatering costs. Another strategy to optimize algal biomass and lipid production would be to combine open ponds and photobioreactors (hybrid system) [68, 69]. This hybrid system would first maximize biomass production in photobioreactors under nutrient-sufficient conditions. The biomass would then undergo nutrient-depleted conditions in open ponds to enhance lipid accumulation.

Significant PV shadowing could be very beneficial during the hottest periods, although it penalizes growth during the cold season. The combination of effective light collection for electricity production with light distribution strategies for microalgae would be an important design criterion. The adjustment of the PV panels using solar flux tracking mechanisms is options that could dynamically adapt the shadows to the needs of the microalgae. In addition, the LCA was based on the conservative assumption of a 15% PV yield. Improvement of the PV efficiency should mechanically contribute to reduce the PV coverage for a same electricity production and, thus, increase microalgae productivity.

These improvements should lead to an additional reduction in the resources and climate change impacts. Based on these same criteria, it remains challenging to reach a better performance than soybean and palm tree biodiesel. Despite this issue, it should be emphasized that a fair comparison between the two approaches ought to be carried out under the same climate. The reference scenario is assessed for hotter climates, under which significantly higher photovoltaic and biomass productions are expected. A comparison with European rapeseed biodiesel is probably more relevant for an appropriate assessment of photovoltaic greenhouses that produce algal biofuel.

## Conclusions

The combination of microalgae production with photovoltaic panels offers several advantages, and the main one is to utilize the excess energy from sunlight to feed the large energy demand for biodiesel microalgae. This could, therefore, counteract the strong external energy requirement of microalgae. Coupling biomass production with photovoltaic electricity represents an ideal opportunity to significantly reduce environmental impacts by a factor close to 50% of GHG emissions. However, there is a clear trade-off between electricity and biomass production, as a larger photovoltaic panels coverture would limit microalgae production. Thus, from an energetic point of view, the optimal configuration lies between 10% and 20% of photovoltaic panel coverage. Nevertheless, from an environmental point of view, the best option is 50% photovoltaic panel coverage. However, the difference between impact values obtained for 20% and 50% PV is negligible, while the net energy ratio is 48% higher for 20% PV than for 50% PV coverage. Hence, 20% coverage of photovoltaic panels is a sound and optimal energetic environmental configuration. Taking economics into account, lower photovoltaic panel coverage would probably be more attractive. However, even with a 10% area of photovoltaic panels, the environmental footprint would already significantly decrease. This study was carried out with state-of-the-art technologies, but significant improvements in microalgae productivity or more advanced production processes should rapidly enhance the performances. The challenge is now to maintain a profitable production from an economic point of view, despite the increased technicality of the processes.

## Supplementary information

**Additional file 1: S1.** Facility infrastructure. **S2.** Pipelines and pumping system. **S3.** Machinery. **S4.** Seasonally allocation variation. **S5.** Algae composition. **S6.** Fertilizers and water. **S7.** Downstream process. **S8.** Combustion emissions. **S9.** Annual average electricity production for the whole facility from CIGS photovoltaic panels. **S10.** Data source. **S11.** Biomass and biodiesel productivity for different coverture of photovoltaic panels. **S12.** CED (renewable+non-renewable) and energy production associated with production of 1 MJ biodiesel. **S13.** Monthly variation of NER and FER. **S14.** LCA for biodiesel from rapeseed, palm tree, soybean and conventional diesel. **S15.** Endpoint impact assessment associated with production of 1 MJ biodiesel. **S16.** Global warming impact of 1 MJ biodiesel by case study (anual average). **S17.** Midpoint categories results using energetic allocation method. **S18.** Monthly GHG emissions for different coverture of PV panels. **S19.** Endopoint and midpoint categories results using substitution as allocation method. **S20.** Comparison LCA results between energetic allocation and substitution allocation.

## Data Availability

Not applicable.

## References

[1] Chisti Y (2008). Biodiesel from microalgae beats bioethanol. Trends Biotechnol.

[2] Wijffels RH, Barbosa MJ (2010). An outlook on microalgal biofuels. Science.

[3] Lardon L, Hélias A, Sialve B, Steyer J, Bernard O (2009). Life-cycle assessment of biodiesel production from microalgae. Environ Sci Technol.

[4] An J-Y, Sim S-J, Lee J, Kim B (2003). Hydrocarbon production from secondarily treated piggery wastewater by the green alga *Botryococcus braunii*. J Appl Phycol.

[5] Chisti Y (2007). Biodiesel from microalgae. Biotechnol Adv.

[6] Sheehan J, Dunahay T, Benemann J, Roessler P. A Look Back at th U.S. Department of Energy’s Aquatic Species Program: biodiesel from Algae Close-Out report. Golden, CO: Department of Energy, National Renewable Lab, 1998 July. Report No.: NREL/TP-580-24190.

[7] Pulz O (2001). Photobioreactors: production systems for photoautotrophic microorganisms. Appl Microbiol Biotechnol.

[8] Chisti Y (2013). Raceways-based production of algal crude oil. Green..

[9] Collet P, Lardon L, Hélias A, Bricout S, Lombaert-Valot I, Perrier B (2014). Biodiesel from microalgae—life cycle assessment and recommendations for potential improvements. Renew Energy..

[10] Minhas A, Hodgson P, Barrow C, Adholeya A (2016). A review on the assessment of stress conditions for simultaneous production of microalgal lipids and carotenoids. Front Microbiol..

[11] Sibi G, Shetty V, Mokashi K (2016). Enhanced lipid productivity approaches in microalgae as an alternate for fossil fuels—a review. J Energy Inst.

[12] Singh P, Kumari S, Guldhe A, Mirsra R, Rawat I, Bux F (2016). Trends and novel strategies for enhancing lipid accumulation and quality in microalgae. Renew Sustain Energy Rev..

[13] Olofsson M, Lamela T, Nilsson E, Bergé J, del Pino V, Uronen P (2012). Seasonal variation of lipids and fatty acids of the microalgae *Nannochloropsis* oculata grown in outdoor large-scale photobioreactors. Energies.

[14] Parlevliet D, Moheimani NR (2014). Efficient conversion of solar energy to biomass and electricity. Aquat Biosyst..

[15] Jez S, Fierro A, Dibenedetto A, Aresta M, Busi E, Basosi R (2017). Comparative life cycle assessment study on environmental impact of oil production from micro-algae and terrestrial oilseed crops. Bioresour Technol.

[16] Luque A, Hegedus S (2011). Handbook of photovoltaic science and engineering.

[17] Calderon A. Energy Life Cycle Assessment (LCA) of silicon-based photovoltaic technologies and the influence of where it is manufactured and installed (Master thesis). Barcelona: Universitat de Barcelona; 2014.

[18] ISO. Environmental management-Life cycle assessment- Principles and frameworks. Switzerland: International Organization for Standardization, 2006 July. Report No.: ISO 14040:2006.

[19] Prè-Consultants. SimaPro 8.3. 2017. p. LCA software.

[20] Csyrnek-Deletre M, Smyth B, Murphy J (2017). Beyond carbon and energy: the challenge in setting guidelines for life cycle assessment of biofuels systems. Renew Energy..

[21] Passel H, Dhaliwal H, Reno M, Wu B, Amotz A, Ivry E (2013). Algae biodiesel life cycle assessment using current commercial data. J Environ Manag.

[22] Huo H, Wang M, Lloyd C, Putsche V (2008). Life cycle assessment of energy and greenhouse gas effects of soybean-derived biodiesel and renewable fuels. Environ Sci Technol.

[23] Ho S, Chang J, Lai Y, Chen C (2014). Achieving high lipid productivity of a thermotolerant microalga Desmodesmus sp. F2 by optimizing environmental factors and nutrient conditions. Bioresour Technol..

[24] Adams C, Godfrey V, Wahlen B, Seefeldt L, Bugbee B (2013). Understanding precision nitrogen stress to optimize the growth and lipid content tradeoff in oleaginous green microalgae. Bioresour Technol.

[25] ANL, NREL, PNNL. Renewable diesel from algal lipids: an integrated baseline for cost, emissions, and resource potential from a harmonized model. U.S.: Argonne National Laboratory, National Renewable Energy Laboratory, Pacific Northwest National Laboratory, 2012 June. Report No.: ANL/ESD/12-4; NREL/TP-5100-55431; PNNL-21437.

[26] NREL. Process design and economics for the production of algal biomass: algal biomass production in open pond systems and processing through dewatering for downstream conversion. Golden, CO U.S. Department of Energy Office of Energy Efficiency & Renewable Energy, 2016 February. Report No.: NREL/TP-5100-64772.

[27] Chisti Y, Bux F, Chisti Y (2016). Large-Scale Production of Algal Biomass: Raceway Ponds. Algae biotechnology green energy and technology.

[28] Rogers J, Rosemberg J, Guzman B, Oh V, Mimbela L, Ghassemi A (2014). A critical analysis of paddlewheel-driven raceway ponds for algal biofuel production at commercial scales. Algal Res.

[29] Chisti Y (2013). Constraints to commercialization of algal fuels. J Biotechnol.

[30] Fagerston K. Measurement of direct nitrous oxide emissions from microalgae cultivation under oxic and anoxic conditions (Master thesis). Fort Collins, CO Colorado State University; 2011.

[31] IPCC. Chapter 4. Indirect N2O Emissions from Agriculture. Good Practice Guidance and Uncertainty Management in National Greenhouse Gas Inventories. Hayama, Japan: Institute of Global Environmental Strategies (IGES), IPCC, 2002.

[32] Li Y, Zhang Q, Zang Z, Wu X, Cong W (2014). Evaluation of power consumption of paddle wheel in an open raceway pond. Bioprocess Biosyst Eng.

[33] Beal C, Gerber L, Sills D, Huntley M, Machesky S, Walsh M (2015). Algal biofuel production for fuels and feed in a 100-ha facility: a comprehensive techno-economic analysis and life cycle assessment. Algal Res.

[34] Milnes M. The mathematics of pumping water. AECOM Design build & The Royal Academy of Engineering; 2017. http://www.raeng.org.uk/publications/other/17-pumping-water. Accessed 11 Jan 2018.

[35] Haas M, McAloon A, Yee W, Foglia T (2006). A process model to estimate biodiesel production costs. Bioresour Technol.

[36] Jungbluth N, Chudacoff M, Dauriat A, Dinkel F, Doka G, Faist-Enmenegger M, et al. Life cycle inventories of bioenergy. Final report ecoinvent data v2.0 Dübendorf: Swiss Centre for Life Cycle Inventories, 2007 Report No.: 17.

[37] Stoppato A (2008). Life cycle assessment of photovoltaic electricity generation. Energy..

[38] Bekkelund K. Life Cycle assessment of thin film solar panels (Master Thesis). Norwegian University of Science and Technology; 2013.

[39] Amarakoon S, Vallet C, Curran MA, Haldar P, Metacarpa D, Fobare D (2008). Life cycle assessment of photovoltaic manufacturing consortium (PVMC) copper indium gallium (di)selenide (CIGS) modules. Int J Life Cycle Assess.

[40] Jungbluth N, Stucki M, Flury K, Frischknecht R, Busser S. Life cycle inventories of photovoltaics. Uster: ESU-services, Swiss Federal Office of Energy, 2012 September. Report No.

[41] Vries S, Van der Ven G, Van Ittersum M, Giller K (2010). Resource use efficiency and environmental performance of nine major biofuel crops, processed by first-generation conversion techniques. Biomass Bioenergy.

[42] Goedkoop M, Heijungs R, Huijbregts M, De Schryver A, Struijs J, Zelm R. ReCiPe 2008, A life cycle impact assessment method which comprises harmonised category indicators at the midpoint and the endpoint level. Holland: PRé Consultants; CML, University of Leiden; Radboud University and RIVM, 2009 January. Report No.: I: Characterization.

[43] Béchet Q, Shilton A, Park J, Craggs R, Guieysse B (2011). Universal temperature model for shalow algal ponds provides improved accurancy. Environ Sci Technol.

[44] Quinn J, Davis R (2015). The potentials and challenges of algae based biofuels: a review of the techno-economic, life cycle, and resource assessment modeling. Bioresour Technol.

[45] Béchet Q, Coulombier N, Vasseur C, Lasserre T, Le Dean L, Bernard O (2018). Full-scale validation of an algal productivity model including nitrogen limitation. Algal Res.

[46] Jian H, Jing Y (2015). Peidong Z.

[47] Batan L, Quinn J, Willson B, Bradley T (2010). Net energy and greenhouse gas emission evaluation of biodiesel derived from microalgae. Environ Sci Technol.

[48] Yang F, Xiang W, Sun X, Wu H, Li T, Long L (2014). A novel lipid extraction method from wet microalgae *Picochlorum* sp. at room temperature. Mar Drugs..

[49] Dones R, Bauer C, Bolliger R, Burger B, Heck T, Röder A, et al. Life cycle inventories of energy systems: results for current systems in Switzerland and others UCTE countries. Data v2.0. Ecoinvent Report Dübendorf: Swiss Centre for Life Cycle Inventories, 2007 December. Report No.: 5.

[50] Barbera E, Sforza E, Vecchiato L, Bertucco A (2017). Energy and economic analysis of microalgae cultivation in a photovoltaic-assisted greenhouse: *Scenedesmus obliquus* as a case study. Energy..

[51] Trypanagnostopoulos G, Kavga A, Souliotis M, Tripanagnostopoulos Y (2017). Greenhouse performance results for roof installed photovoltaic. Renew Energy..

[52] Li C, Wang H, Miao H, Ye B (2017). The economic and social performance of integrated photovoltaic and agricultural greenhouses systems: case study in China. Appl Energy..

[53] D’Avino L, Dainelli R, Lazzeri L, Spugnoli P (2015). The role of co-products in biorefinery sustainability: energy allocation versus substitution method in rapeseed and carinata biodiesel chains. J Clean Prod..

[54] Directive 2009/28/EC of the European Parliament and of the council on the promotion of the use of energy from renewable sources and amending and subsequently repealing Directives 2001/77/EC and 2003/30/EC., art. 23 par. 4 (2009).

[55] Sialve B, Bernet N, Bernard O (2009). Anaerobic digestion of microalgae as a necessary step to make microalgal biodiesel sustainable. Biotechnol Adv.

[56] Collett P, Hélias A, Lardon L, Ras M, Goy R, Steyer J-P (2011). Life-cycle assessment of microalgae culture coupled to biogas production. Bioresour Technol.

[57] Quinn J, Smith T, Dwones C, Quinn C (2014). Microalgae to biofuels lifecycle assessment- Multiple pathway evaluation. Algal Res.

[58] Chu W (2017). Strategies to enhance production of microalgal biomass and lipids for biofuel feedstock. Eur J Phycol.

[59] Xue J, Balamurugan S, Li DW, Liu YH, Zeng H, Wang L (2017). Glucose-6-phosphate dehydrogenase as a target for highly efficient fatty acid biosynthesis in microalgae by enhancing NADPH supply. Metab Eng.

[60] Trentacoste E, Shrestha R, Smith S, Glé C, Hartmann A, Hildebrand M (2013). Metabolic engineering of lipid catabolism increases microalgal lipid accumulation without compromising growth. Proc Natl Acad Sci.

[61] Li D, Zhao Y, Ding W, Zhao P, Xu JW, Li T (2017). A strategy for promoting lipid production in green microalgae *Monoraphidium* sp. QLY-1 by combined melatonin and photoinduction. Bioresour Technol..

[62] Dash A, Banerjee R (2017). Enhanced biodiesel production through phyco-myco co-cultivation of Chlorella minutissima and *Aspergillus awamori*: an integrated approach. Bioresour Technol.

[63] Zhang Z, Ji H, Gong G, Zhang X, Tan T (2014). Synergistic effects of oleaginous yeast *Rhodotorula glutinis* and microalga *Chlorella vulgaris* for enhancement of biomass and lipid yields. Bioresour Technol.

[64] Yen HW, Chen PW, Chen LJ (2015). The synergistic effects for the co-cultivation of oleaginous yeast-*Rhodotorula glutinis* and microalgae-*Scenedesmus obliquus* on the biomass and total lipids accumulation. Bioresour Technol..

[65] Do Nascimento M, Dublan ML, Ortiz-Marquez JC, Curatti L (2013). High lipid productivity of an Ankistrodesmus-Rhizobium artificial consortium. Bioresour Technol.

[66] Bonnefond H, Grimaud G, Rumin J, Bougaran G, Talec A, Gachelin M (2017). Continuous selection pressure to improve temperature acclimation of Tisochrysis lutea. PLoS ONE.

[67] Gross M, Henry W, Michael C, Wen Z (2013). Development of a rotating algal biofilm growth system for attached microalgae growth with in situ biomass harvest. Bioresour Technol.

[68] Huntley ME, Redalje DG (2006). CO_2_ mitigation and renewable oil from photosynthetic microbes: a new appraisal. Mitig Adapt Strateg Glob Change..

[69] Narala RR, Garg S, Sharma KK, Thomas-Hall SR, Deme M, Li Y (2016). Comparison of microalgae cultivation in photobioreactor, open raceway pond, and a two-stage hybrid system. Front Energy Res..

