# Supplementary material for: Optimal integration of microalgae production with photovoltaic panels: environmental impacts and energy balance
Source: Biotechnol Biofuels. 2019 Oct 8;12:239. doi: 10.1186/s13068-019-1579-4 (PMC6781331; doi:10.1186/s13068-019-1579-4)
Supplement: Supplementary file 1 — Additional file 1: S1. Facility infrastructure. S2. Pipelines and pumping system. S3. Machinery. S4. Seasonally allocation variation. S5. Algae composition. S6. Fertilizers and water. S7. Downstream process. S8. Combustion emissions. S9. Annual average electricity production for the whole facility from CIGS photovoltaic panels. S10. Data source. S11. Biomass and biodiesel productivity for different coverture of photovoltaic panels. S12. CED (renewable+non-renewable) and energy production associated with production of 1 MJ biodiesel. S13. Monthly variation of NER and FER. S14. LCA for biodiesel from rapeseed, palm tree, soybean and conventional diesel. S15. Endpoint impact assessment associated with production of 1 MJ biodiesel. S16. Global warming impact of 1 MJ biodiesel by case study (anual average). S17. Midpoint categories results using energetic allocation method. S18. Monthly GHG emissions for different coverture of PV panels. S19. Endopoint and midpoint categories results using substitution as allocation method. S20. Comparison LCA results between energetic allocation and substitution allocation. [file 13068_2019_1579_MOESM1_ESM.docx]

**Additional file 1**

Optimal integration of microalgae production with photovoltaic panels: environmental impacts and energy balance

Marjorie Morales, Arnaud Hélias and Olivier Bernard.

1. S1: Facility infrastructure 3

1.1. Facility layout 3

1.2. Raceways infrastructure 4

1.3. Paddlewheel 4

1.4. Inoculum ponds infrastructure 5

2. S2: Pipelines and pumping system 7

2.1. Piping system 7

2.2. Pumping system 7

3. S3: Machinery 9

3.1. Machinery production 9

3.2. Distillation column 9

3.3. Blower system 9

4. S4: Seasonally allocation variation 10

5. S5: Algae composition 11

6. S6: fertilizers and water 12

6.1. Fertilizers and water requirements 12

6.2. CO_2_ delivery 14

7. S7: Downstream process 15

7.1. Algae harvesting 15

7.2. Algae transformation 15

8. S8: Combustion emissions 17

9. Additional file 9: Annual average electricity production for the whole facility from CIGS photovoltaic panels. 18

10. S10: Data source 19

11. S11: biomass and biodiesel productivity for different coverture of photovoltaic panels 20

12. S12: CED (renewable + non-renewable) and energy production associated with production of 1 MJ biodiesel. 21

13. S13: Monthly variation of NER and FER 22

14. S14: LCA for biodiesel from Rapeseed, Palm tree, Soybean and conventional diesel. 23

14.1. Vegetable biodiesel 23

14.2. Conventional diesel 24

14.3. Substitution method for vegetable biodiesel and conventional diesel 25

15. S15: Endpoint impact assessment associated with production of 1 MJ biodiesel. 26

16. S16: Global warming impact of 1 MJ biodiesel by case study (annual average). 27

17. S17: Midpoint categories results using energetic allocation method 28

18. S18: Monthly GHG emissions for different coverture of PV panels 34

19. S19: Endpoint and Midpoint categories results using substitution as allocation method 35

20. S20: Comparison LCA results between Energetic allocation and Substitution allocation method 42

References 49

1. S1: Facility infrastructure
   1. Facility layout

The overall site layout assumes that ponds are grouped into unit “modules” of about 5 ha (50 868 m^2^) each. Each module represents a standard greenhouse, constructed with low-emissivity (low E) glass (KGlass^TM^ from Pilkington: thickness=4 mm, transmittance=82%, density= 10 kg·m^-2^, lifespan= 30 years) [[1](#_ENREF_1)] for walls and roof, supported by a steel frame. Low E is an essential contributor to energy conservation, since it reflects energy back into the greenhouse, achieving much lower heat loss than ordinary glass [[1](#_ENREF_1)], and eventually extending the production period. The greenhouse structure also includes a climate control system through ventilation so that medium temperatures are maintained close to the optimal growth temperature of the microalgae. The ventilation system consists of favouring airflow by opening and closing the windows (flow rates fixed to 50 m^3^·s^-1^·greenhouse^-1^ and 500 m^3^·s^-1^·greenhouse^-1^, windows are closed and open, respectively).

**Figure 1.1.** Schematic diagram for overall facility layout, depicting location of modules and connections to overall circulation networks.

The full facility contains 122 ha of biomass production raceways grouped into 24 individual greenhouses (including 2 for inoculum ponds) connected via a network of pipelines and roadways. The greenhouses form a uniform grid of four columns by six rows. The rows comprise the raceway pond modules as well as the inoculum ponds. The facility also includes a dewatering section, a nutrient and freshwater storage section, and algal biomass conversion sections. Roads with access to all modules are 2 m wide between columns and 2 m wide between rows. The module dimensions include spacing for piping, electricity and roads on the border to access to the ponds. The nutrient and freshwater storage section provides bulk storage for water and nutrient inputs, while biodiesel is stored in the esterification section.

- 1. Raceways infrastructure

Open raceway ponds are widely adopted in microalgae cultivation [[2](#_ENREF_2)]. Cultivation system is designed with a total production capacity of 145 ha, considering a total of 110 raceways. A schematic diagram of the open raceways ponds in the greenhouse is shown in **Figure 1.2.** The 8348 m^2^ raceways ponds were 310 m long, 30 m wide and 0.30 m depth, divided by a central baffle (0.30 m wide) to form a raceway. The culture depth was 0.25 m with a volume of 2184 m^3^ per raceway. Raceways are operated in a continuous way during 330 days per year. Ponds are directly excavated and made of polypropylene liner. The whole pond structures, including the walls and bottom were built in polypropylene.

**Figure 1.2.** Schematic layouts for the raceways configurations and greenhouse

- 1. Paddlewheel

The configuration of blades directly determines power consumption for paddlewheels. The results reported by [Li, Zhang [2](#_ENREF_2)] demonstrated that more interaction areas between blades and fluid led to lower paddlewheel efficiency. Therefore, the flat blades are the most efficient configuration. A eight-blade paddlewheel was equipped to drive the fluid by a motor 1 kW model 90 SH/4 HM [[3](#_ENREF_3)] for raceways and inoculum ponds each, and functioning 12 h per day. The dimensions of paddlewheel are detail in **Figure 1.3**.

**Figure 1.3.** Schematic layouts for the paddlewheel configuration in the raceway

The energy required (P in Watt) by shaft in the paddlewheel [[2](#_ENREF_2)]:

$P=\frac{9.8\cdot Q\cdot w\cdot h}{e}$ Eq. 1

In which, Q is the volumetric flow rate (m^3^·s^-1^), *w* is the unit mass of water (1000 kg·m^-3^), *e* is the paddlewheel and drive system efficiency (40% assumed), h is the total head loss and 9.8 is the conversion factor in W·s·kg^-1^·m^-1^.

The h was calculated using the equation 2 [[4](#_ENREF_4)]:

$h=h_{b}+h_{c}$ Eq.2

Where, *h_b_* is the head loss in bends and *h_c_* is the friction loss across the length of the raceways. *h_b_* is calculated by equation 3, in which *K* is the kinetics loss coefficient for 180^o^ bends (Theoretically=2) [[4](#_ENREF_4)], *v* is the velocity of the raceway (0.3 m/s for raceways and inoculum ponds) and *ɡ* is the acceleration due to the gravity (9.8 m·s^2^), resulting in *h_b_* = 0.018

$h_{b}=\frac{K\cdot v^{2}}{2\cdot g}$ Eq. 3

*h_c_* is calculated by the Manning’s equation (equation 4). In which n is the roughness factor (0.015 for polyethylene) [[4](#_ENREF_4)], R is the channel hydraulic radius (see equation 5), and L is the channel length (310 and 160 m for raceways and inoculum ponds), resulting in *h_c_*= 0.017 for raceways and *h_c_*= 0.0049 for inoculum ponds.

$h_{c}=v^{2}\cdot n^{2}\cdot\left( \frac{L}{R^{3/4}} \right)$ Eq. 4

The hydraulic diameter R is defined as follows in equation 5 [[5](#_ENREF_5)]:

$R=\frac{4\cdot a\cdot b}{a+2\cdot b}$ Eq. 5

In Eq. 5, *a* is the width of the channel (14.85 m and 7.5 m, for raceways and inoculum ponds, respectively) and *b* is the average depth of the broth in it (0.25 m and 0.3m for raceways and inoculum ponds, respectively).

The paddlewheel are made of high-density polyethylene (paddles) and steel (motor and torque). The lifespan of the agitation system is estimated to be 20 years for paddles and motor [[6](#_ENREF_6)]. The energy required (P) is 950 W and 243 W for raceways and inoculum ponds (0.11 W·m^-2^ for raceways and inoculum ponds)

- 1. Inoculum ponds infrastructure

The infrastructure of the inoculum ponds is the same of the raceways. 40 units of inoculum ponds were installed in the facility, one to inoculate 3 raceways (with 10% volume). The area is 2186.8 m^2^ (160 m long, 15 m wide, 0.35 m depth) with a volume of 656 m^3^ (culture depth of 0.3 m).

**Figure 1.4.** Schematic layouts of inoculum ponds in greenhouse.

1. S2: Pipelines and pumping system

- 1. Piping system

The piping is a critical factor as it covers a large land footprint. Each pipeline considers a valve to open or close the pass of water, nutrients and/or inoculum in each raceway and inoculum pond. The piping system includes five independent pipelines:

- **Harvesting pipeline:** it considers the water circulation from each cultivation raceway to dewatering system, covering a length of 2,503 m with a PVC pipeline of 24-inch diameter.
- **Dewatering recirculation pipeline:** it considers the recirculation from clarified water from dewatering system to each raceway, covering a length of 2,422 m with a PVC pipeline of 24-inch diameter.
- **Nutrients & freshwater pipeline:** it considers the flux transport of the nutrients mix with freshwater (to compensate the evaporation rates) from the nutrients & freshwater section to each cultivation raceways and inoculum ponds. It covers a length of 2,503 m with a PVC pipeline of 24-inch diameter for raceways and 162 m length with 16-inch diameter for inoculum ponds.
- **Inoculum pipelines:** it considers the inoculum media transport from each inoculum ponds to the raceways. It covers a length of 820 m with a PVC pipeline of 24-inch diameter.
- **CO_2_ pipelines:** it considers HDPE pipelines with sufficient thickness to support pressure up to hundred psig. It consists in three pipelines with different diameters and lengths, following the model proposed by [NREL [7](#_ENREF_7)]: a pipeline central (truck line), a pipeline running through columns and other in the individual ponds. The central truck line consists of a 20-inch truck line from the power plant to the end of the facility. The truck lines supplies 12-inch branch lines running down the aisles between raceways. Within each raceway, a network of 3 inch piping delivers (spargers) the CO_2_ to the sumps in individual ponds. Gas injection is realized by spargers at the bottom of 1 m deep sump.
  1. Pumping system

The Equation 6 calculates the energy requirement per pump,

$P=\frac{Q\cdot H\cdot\rho\cdot g}{e}$ Eq. 6

Where, P is the pump power (W), Q is the flow rate through the pipe (m^3^/s), ρ is the water density (1000 kg/m^3^), *e* is the pumping efficiency (50% [[4](#_ENREF_4)]) and H is the total system head,

$H=H_{S}+H_{D}$ Eq. 7

Where, *H_S_* is the static head and *H_D_* is the dynamic head loss. *H_S_* represents the physical change in elevation between the surface of the reservoir and the point of discharge into the receiving tank (or pond). The dynamic head is generate as a result of friction within the system and is calculated using the Darcy Weisbach equation (Equation 8),

$H_{D}=\frac{K\cdot v^{2}}{2\cdot g}$ Eq. 8

Where, *v* is the velocity in the pipe (m/s), ɡ is the acceleration due to the gravity (9.8 m/s^2^) and K is the loss coefficient. Due to there be no difference of elevation in the facility, *H_s_* was not considered, and the total system head is equal to the dynamic head.

The velocity in the pipe (*v*) is calculated using the equation 9.

$v=\frac{Q}{A}$ Eq. 9

Where, Q is the flow rate through the pipe (m^3^/s) and A is the cross sectional area (m^2^), which is calculated using the equation 10,

$A=\frac{\pi\cdot D}{4}$ Eq. 10

Where, D is the diameter of the pipe (m), While, the loss coefficient (K) is calculated using the equation 11, where *K_fittings_* is associated with the fitting used in the pipeline of the system to pump the water from reservoir to the receiving tank. *K_pipe_* is associated with the straight lengths of pipe, defined in the equation 12, where f is the friction coefficient, L is the pipe length (m) and D is the pipe diameter (m).

$K=K_{\mathrm{fittings}}+K_{\mathrm{pipe}}$ Eq. 11

$K_{\mathrm{pipe}}=\frac{f\cdot L}{D}$ Eq. 12

The friction coefficient (f) can be found using a modified version of the Colebrook White equation (Equation 13).

$f=\frac{0.25}{\left[ \log\left\{ \frac{k}{3.7\cdot D}+\frac{5.74}{\mathrm{Re}^{0.9}} \right\} \right]^{2}}$ Eq. 13

Where, *k* is roughness factor (m) and *Re* is the Reynolds number (determined with the Equation 14). The pipe roughness factor is a standard value based upon the material of the pipe, including any internal coating, the value considered was 0.3 mm.

$Re=\frac{v\cdot D}{\vartheta}$ Eq. 14

In the Equation 14, υ represent the kinematic viscosity of water (1.31·10^-6^ m^2^·s^-1^). Values for *K_fittings_* can be obtained from standars tables and a total *K_fittings_* can be calculated by adding all the *K_fittings_* values (**Table 2.1**):

**Table 2.1.** K fittings values in pipelines

|  | **90 ° bends**  **(K_fittings_ value= 0.75)** | | **Butterfly valves**  **(K_fittings_ value= 0.3)** | |
| --- | --- | --- | --- | --- |
| **Pipeline** | **No of items** | **Total** | **No of items** | **Total** |
| Harvesting | 2 | 1.5 | 22 | 6.6 |
| Dewatering recirculation | 2 | 1.5 | 22 | 6.6 |
| Nutrients & freshwater (to raceways) | 2 | 1.5 | 22 | 6.6 |
| Nutrients & freshwater (to inoculum ponds) | 2 | 1.5 | 2 | 0.6 |
| Inoculum pipelines | 0 | 0 | 22 | 6.6 |

The pumping power values were calculated for each raceways and inoculum pond considering each pipeline properties (see section 2.1 in Additonal file 2).

1. S3: Machinery
   1. Machinery production

The number of machinery units (n) in the facility depends of the capacity of the machinery (C) in m^3^/h, the inlet flux (F) in m^3^/d and time functioning by day (t) in h/d, as show Equation 15:

$n=\frac{110\cdot F}{C\cdot t}$ Eq. 15

The flux considered determining the number of unit, is the flux at maximal facility capacity. The machinery capacity, time functioning by day and the number of machinery units are show in **Table 3 (in article).**

- 1. Distillation column

The power require by the distillation column, was determined using the equation 16 [[4](#_ENREF_4)], in which *Cp* is the specific heat of hexane (2.26 KJ·kg^-1^ ^o^C^-1^), Tb is the boiling point temperature of hexane (69 ^o^C) and w is the input flux of hexane (kg hexane/h).

$P\left( \mathrm{kW} \right)=\frac{C_{p}\cdot w\cdot\left( T_{b}-20 \right)\cdot75}{3600}$ Eq. 16

- 1. Blower system

Carbon is provided to the facility as a 14% CO_2_ waste stream form a power plant facility located 2 km from the algae production facility. Due to complex fluid mechanics associated with compressible flow, intermittent demand, line packing, and temperature fluctuations, the energy required for transporting the CO_2_ stream was approximated by assuming compression to 2 atm using the following equation [[8](#_ENREF_8)],

$E_{\mathrm{comp}}\left( \frac{\mathrm{kJ}}{\mathrm{kg}} \right)=\frac{C_{p}\cdot T}{\eta}\cdot\left( \left( \frac{P_{f}}{P_{0}} \right)^{\frac{\gamma-1}{\gamma}}-1 \right)$ Eq. 17

Where, $C_{p}$ is the specific heat capacity (0.85 kJ/kg-K), 𝑇 is the temperature (298 K), 𝜂 is the compression efficiency (0.85), 𝑃𝑓 is the final pressure (2 atm), 𝑃𝑜 is the initial pressure (1 atm), and 𝛾 is the ratio of specific heats (1.4). The total energy required for carbon delivery is calculated by multiplying the result of Equation 17 by the mass of gas transported per day, wich is variable depending of biomass productivity.

1. S4: Seasonally allocation variation


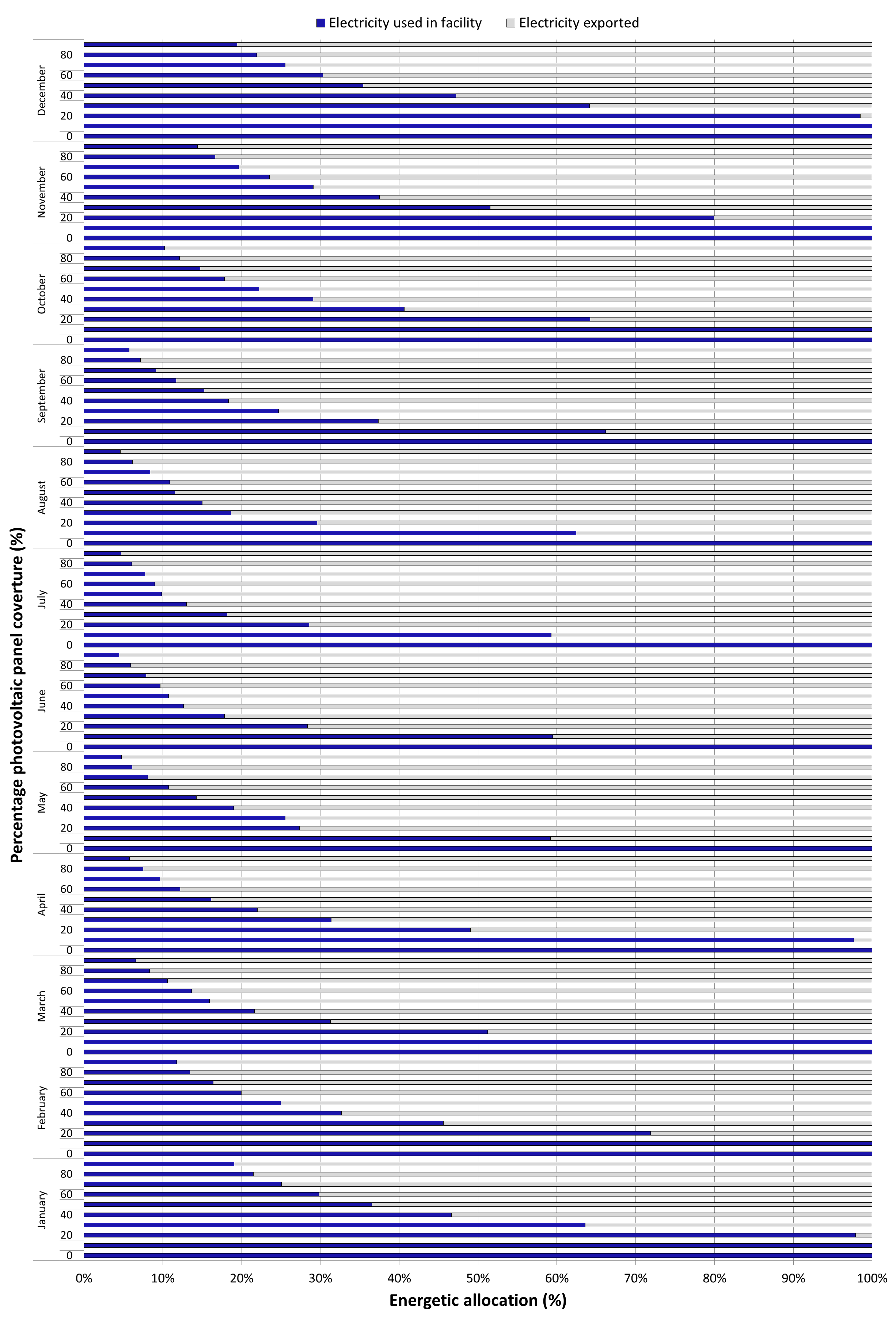
 **Figure 4.1.** Monthly average allocation at variable photovoltaic panel coverture.

1. S5: Algae composition

**Table 5.1** and **Table 5.2** provide the biomass general information and compositional details. The analysis considers a 47%% and 53.8% lipid content (of dry basis content biomass), for *Chlorococcum sp.* and *Desmodesmus sp*., respectively.

**Table 5.1.** General characteristics of algae species case studies

|  | ***Chlorococcum sp*** | **[Ref.]** | ***Desmodesmus sp*.** | | **[Ref.]** |
| --- | --- | --- | --- | --- | --- |
| Optimal Temperature (^o^C) | 25 | [[9](#_ENREF_9)] | 35 | [[10](#_ENREF_10), [11](#_ENREF_11)] | |
| Minimum temperature (^o^C) | 0 |  | 5 |  |  |
| Maximum temperature (^o^C) | 30 |  | 46 |  |  |
| Optimal growth rate (d^-1^) | 2.88 | [[12](#_ENREF_12)] | 2.50 | [[13](#_ENREF_13)] | |
| Optimal Irradiance (µmol photons m^-2^ s^-1^) | 220 | [[12](#_ENREF_12)] | 700 | [[13](#_ENREF_13)] | |

Note: * Assumed value, no data available.

The element composition of organic and dry basis solid content is based by using the chemicals formula for each biochemical compound (carbohydrate (C_6_H_12_O_6_), protein (C_4.43_H_7_O_1.44_N_1.17_), lipids (C_40_H_74_O_5_), DNA/RNA (C_9.5_H_13.75_O_6_N_3.75_), Chlorophyll (C_55_H_70_O_6_N_4_) and Fibre (C_6_H_12_O_6_) [[6](#_ENREF_6)]).

The phosphorous composition was determined by phosphorous-nitrogen (N/P) ratio. The N/P ratio of nutrient replete phytoplankton ranges from about 5 to 19, with most observations around Redfield ratio of 16. However, under severe nitrogen limitation, N/P is < 5 [[14](#_ENREF_14)]. This study considers a N/P ratio of 5 for both species.

**Table 5.2.** Chemical and elemental composition of the microalgae species under N-limitation.

|  | ***Chlorococcum sp.*** | **[Ref.]** | ***Desmodesmus sp.*** | **[Ref.]** |  |
| --- | --- | --- | --- | --- | --- |
| **Chemical composition of dry solid basis content (%)** |  | [[15](#_ENREF_15)] |  | [[10](#_ENREF_10)] |  |
| Carbohydrate | 37 |  | 35.2 |  |  |
| Protein | 16 |  | 11 |  |  |
| Lipids | 47 |  | 53.8 |  |  |
| **Element composition of dry**  **solid basis content (%)** | | | | | |
| Carbon | 56.2 |  | 57.5 |  |  |
| Hydrogen | 9.0 |  | 9.4 |  |  |
| Nitrogen | 2.68 |  | 1.9 |  |  |
| Oxygen | 32.1 |  | 32.2 |  |  |
| Phosphorous | 0.53 |  | 0.38 |  |  |
| Trace elements | 4.3* |  | 3.5* |  |  |

*Calculated by difference.

1. S6: fertilizers and water
   1. Fertilizers and water requirements

In addition to carbon dioxide, algal growth requires nitrogen (N) and phosphorous (P) as principal nutrients [[16](#_ENREF_16)]. Nutrients requirements for the inoculum ponds and raceways are assumed to be met using diammonium phosphate (DAP, 18% N, 20.2% P) for phosphorous requirements, and ammonium nitrate (NH_4_NO_3_, 35%N) for nitrogen requirements at 20% w/w each. The requirement of N-fertilizer and P-fertilizer by the microalgae was determined using the equation:

$\frac{kg (fertilizer)}{\mathrm{day}}=\frac{\mathrm{kg}\left( Net element required \right)}{\mathrm{day}}\cdot\frac{100}{\% element in fertilizer}$ Eq. 18

Where:

$\frac{\mathrm{kg}\left( Net element required \right)}{\mathrm{day}}= \frac{{kg biomass}_{\mathrm{DW}}}{\mathrm{day}}\cdot\frac{element in biomass(\%)}{100}\frac{100}{uptake efficiency (\%)}$ Eq. 19

The uptake efficiency for phosphorous and nitrogen considered were 100%. Percentages of N and P in biomass vary depending on the microalgae specie (see **Table 1.2**). In the case of N, a fraction of the element is provided by DAP:

$\frac{kg N (from DAP)}{\mathrm{day}}=\frac{kg DAP}{\mathrm{day}}\cdot\frac{\% N in DAP}{100}$ Eq. 20

And other fraction is provided by ammonium nitrate:

$\frac{kg N (from \mathrm{NH}_{4}\mathrm{NO}_{3})}{\mathrm{day}}=\frac{kg (Net element required)}{\mathrm{day}}- \frac{kg N (from DAP)}{\mathrm{day}}$ Eq. 21

Therefore,

$\frac{kg (\mathrm{NH}_{4}\mathrm{NO}_{3})}{\mathrm{day}}=\frac{kg N \left( \mathrm{from}\mathrm{NH}_{4}\mathrm{NO}_{3} \right)}{\mathrm{day}}\cdot\frac{100}{\% N in \mathrm{NH}_{4}\mathrm{NO}_{3}}$ Eq. 22

The fertilizers requirements in the inoculum ponds and raceways were calculated, depending of the specie. For *Chlorococcum sp.* the nitrogen and phosphorous fertilizers are 0.01 kg NH_4_NO_3_/kg algae biomass DW (0.03 kg N/kg algae biomass dry weight) and 0.003 kg DAP/kg algae biomass DW (0.005 kg P/ kg algae biomass dry weight). While, in the case of *Desmodesmus sp.* are 0.01 kg NH_4_NO_3_/kg algae biomass DW (0.02 kg N/kg algae biomass dry weight) and 0.002 kg DAP/kg algae biomass DW (0.004 kg P/ kg algae biomass dry weight). These values, 0.03 and 0.02 kg N/kg algae biomass dry weight, for *Chlorococcum sp*. and *Desmodesmus s.,* respectively are similar to the reported by [Collet, Lardon [6](#_ENREF_6)] for biodiesel production using *Nannochloropsis occulata* at nitrogen starvation (0.04 kg N/kg algae biomass dry weight). The areal fertilizers requirements in the raceways vary depending of the season variation of the biomass productivity as show **Figure 6.1**.


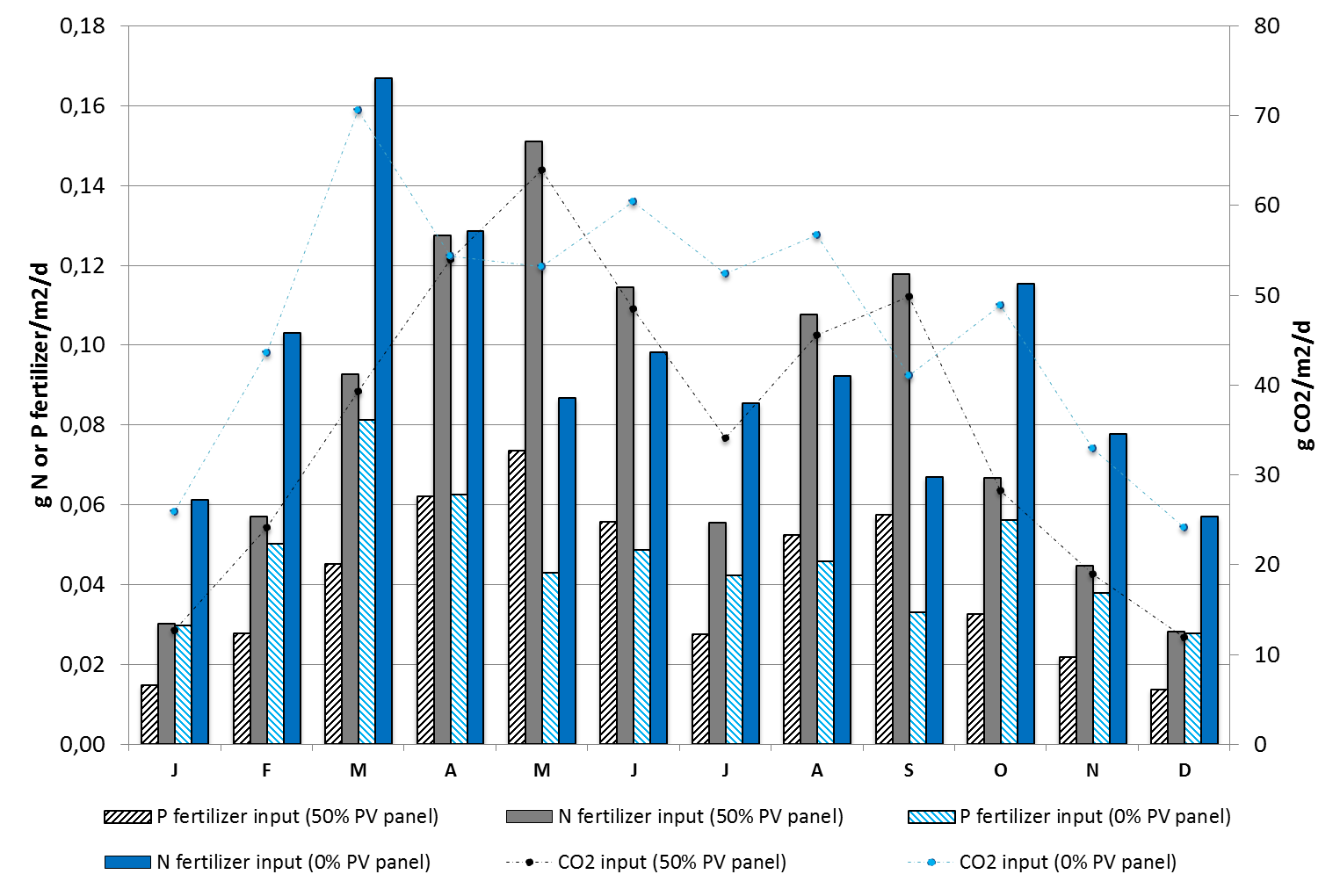


**Figure 6.1.** Monthly average fertilizers and CO_2_ consumption at 0% and 50% coverture of photovoltaic panels.

The supply of freshwater is insufficient to support any substantial scale production of algal fuels anywhere. Supply of brackish water is also relatively limited. Therefore, use of seawater and marine algae are the only realistic options for making algal fuels. Using seawater for algae culture, unfortunately, does not totally eliminate the need for freshwater. Freshwater is needed to compensate for evaporative losses and a consequent increase in culture salinity. Evaporative loss depends on the local climatic conditions, particularly on the irradiance level, the air temperature, the wind velocity and the absolute humidity [[17](#_ENREF_17)]. The evaporation was variable and depending of the season and the values were obtained from a mathematical model (**Figure 6.2**).


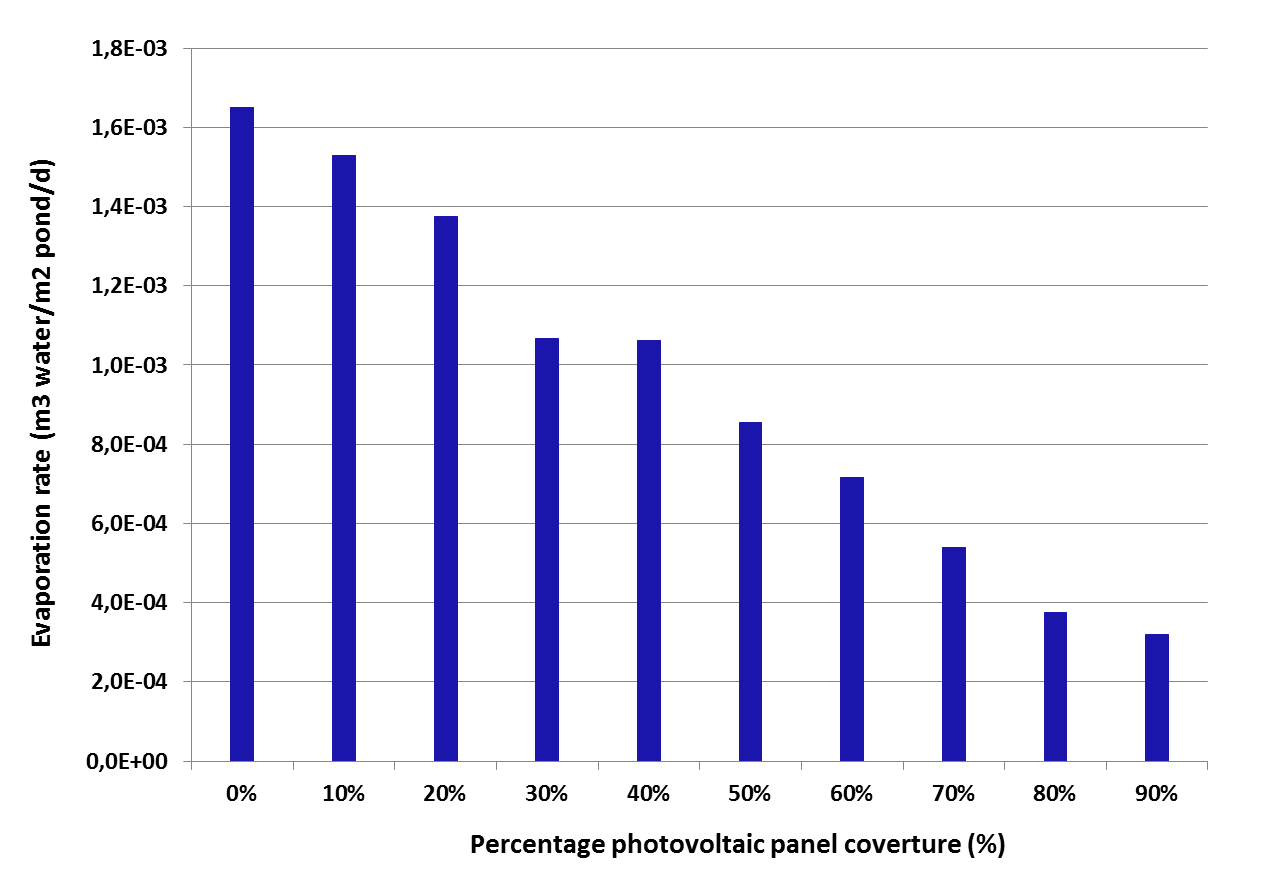


**Figure 6.2.** Monthly average evaporation rate in raceways

The water to the facility is provided by pipeline transport from a nearby local marine water resource and freshwater from outside facility boundaries. The transport of water used in the facility was excluded in the study.

Marine water is used in the cultivation and inoculum pond, while freshwater is required for fertilizers dilution and make up for water losses (primarily via pond evaporation) (Eq. 23) The blowdown volume from dewatering processes, was assumed to be equal to water requirement.

$Volume freshwater (\frac{m^{3}}{\mathrm{day}})=dilution water fertilizer \left( \frac{m^{3}}{\mathrm{day}} \right)+water evaporated \left( \frac{m^{3}}{\mathrm{day}} \right)$ Eq.23

- 1. CO_2_ delivery

In order to maintain a high photosynthetic rate, the influx of carbon and energy must be non-limiting. In photoautotrophic growth, energy is provided by light and carbon in the form of CO_2_. The carbon dioxide in the ambient atmosphere is insufficient to support productivities that are biologically feasible under otherwise non limiting conditions [[17](#_ENREF_17)]. The 0.03% CO_2_ content of ambient air is suboptimal for photosynthesis [[18](#_ENREF_18)], hence for optimal microalgae growth, additional CO_2_ must be provided. This is usually done by direct injection of a CO_2_ stream. Production of each ton of algal biomass requires at least 1.83 tons of carbon dioxide [[19](#_ENREF_19)].

In this study CO_2_ is supplied from a nearby fossil fuel power plant by direct injection of flue gas.

The distribution is carry out under moderate pressure using HDPE pipes with sufficient thickness and distributed by a blower system. Similar to fertilizer requirements, carbon requirements depend of biomass productivity and concentration, according to the equation 24.

$\frac{\mathrm{kg}\left( Net C required \right)}{\mathrm{day}}= \frac{{kg biomass}_{\mathrm{DW}}}{\mathrm{day}}\cdot\frac{C in biomass(\%)}{100}\frac{100}{uptake efficiency (\%)}$ Eq. 24

The carbon uptake efficiency for the microalgae was assumed in 75% [[4](#_ENREF_4)]. While, the percentages of C in biomass vary depending of the microalgae specie (see **Table 1.2**). Then, the CO_2_ requirements are calculated using the equation 25. **Figure 6.1** shows the CO_2_ requirements in a year.

$\frac{kg (\mathrm{CO}_{2})}{\mathrm{day}}=\frac{\mathrm{kg}\left( Net C required \right)}{\mathrm{day}}\cdot\frac{100}{27}$ Eq. 25

1. S7: Downstream process
   1. Algae harvesting

Biomass is harvested from the ponds and concentrated through three dewatering steps comprising gravity settlers, membranes and centrifugation to reach a final concentration of 200 g·L^-1^.Clarified water from each step is recycled towards the cultivation raceways, excluding a small fraction that is removed as blowdown to mitigate the build-up of salts and other inorganic compounds .

The dewatering process begins with the primary settling ponds, for which energy demand is low since only pumps are required. The settler has a trapezoidal profile with a volume of 364.1 m^3^ (50 m in length, 1.7 m deep, 8.5 m wide at the top and 0.34 m wide in the bottom). There are a total of 22 settler ponds with a 4 h residence time. The biomass is removed from these trenches by positive displacement pumps (assuming a negligible energy demand). The material harvested from gravity settling is transferred to membranes, while clarified effluent is redirected back towards the raceways through feed pipes, along with additional recycled water from membranes and centrifuges through 3-inch diameter DI pipelines. The settler ponds concentrate the algal biomass from 0.5 to 10 kg·m^-3^, with 90% efficiency (i.e. 10% of the biomass returns to the ponds in the clarified water stream) and reduce the volume of water by a factor of 20.

The second dewatering process uses hollow fibre membranes. This technology was selected for its favourable performance and costs at a commercial scale, such as high reliability, direct scalability and simple thermal, mechanical and chemical management [[7](#_ENREF_7)]. Maintenance and fouling are not problematic or costly, based on a daily cleaning protocol for the membrane modules. The hollow fibre membrane units received biomass at 10 kg·m^-3^ from the settling ponds and concentrate the biomass to 130 kg·m^-3^, with an efficiency of biomass retention close to 100% (assumed here at 99.5%).

Centrifugation takes place after the hollow fibre membranes, during the final dewatering step. It leads to a high biomass concentration [[7](#_ENREF_7)]. The centrifuge concentrates biomass between 130 kg/m^3^ and 200 kg/m^3^, with a dewatering efficiency of 97% (3% of biomass is removed with the clarified water). The 99.8% of the total water inlet in the subsystem is dewatered during all three steps.

- 1. Algae transformation

A 16 kW sonicator was used for cell disruption, processing up to 12 m^3^/h. The lipid extraction was then performed on the 20% wt slurry in a static mixer. The static mixer combines the solvent and algal biomass during lipid extraction. A solvent to algae-DW mass ratio of 10:1 was assumed, with an 80% extraction efficiency and without any electricity requirement. A daily solvent loss of 0.005% was assumed. In order to separate the oil cake (biomass + water) from the hexane-oil mix, the current model uses a biomass-solvent separator. This separator operates at 6 kW, processing 5.7 m^3^·h^-1^. In order to recover the solvent, a distillation column with a maximal capacity of 15.2 m^3^·h^-1^ was used. The recovered hexane is re-circulated towards the static mixer and is mixed with the new hexane flux to compensate for hexane emission losses, while the oil continues onwards to the next transesterification subsystem.

Algal oil with higher phospholipid contents are less suitable for biofuel, since phosphorous reduces the efficiency of the alkaline catalysts used in the transesterification process [[20](#_ENREF_20)]. Phospholipids are of primary concern within the polar lipid fraction for their propensity to form gums and deactivate catalysts. For this reason, a lipid clean up step was included to remove these impurities. The following two assumptions were made for the oil obtained from the distillation column: the phospholipid and free fatty acid contents are negligible in the algal oil [[20](#_ENREF_20)], and the oil contains traces of water and hexane [[21](#_ENREF_21)].

Transesterification is assumed for the conversion of algal oil into biodiesel. The current model is inspired from the process proposed by [Haas, McAloon [20](#_ENREF_20)], for a production of 37854.1 m^3^ biodiesel·y^-1^ (52158.8 ton·y^-1^). This design was based on the use of crude, degummed soybean oil with negligible phospholipid and free fatty acid content as feedstock. The process involves three processing sections: i) transesterification unit where the vegetable oil is subjected to chemical transesterification to produce fatty acid methyl esters (biodiesel) and co-product glycerol, ii) a biodiesel purification section where the methyl esters were refined to meet biodiesel specifications and iii) a glycerol recovery section. The final product obtained is biodiesel with a lower than 0.005% (v/v) water content.

1. S8: Combustion emissions

**Table 8.1.** Tailpipe emissions

| **Reference: [**[**22**](#_ENREF_22)**]** | **Value (kg/km)** | | |
| --- | --- | --- | --- |
| Pollutant name | Diesel, low sulphur | 5% Biodiesel, rape seed methyl ester | 100% Biodiesel, rape seed methyl ester (calculated) |
| Carbon monoxide, fossil | 6.10E-04 | 6.09E-04 | 5.90E-04 |
| Carbon monoxide, biogenic | 0 | 2.86E-05 | 5.72E-04 |
| Carbon dioxide, fossil | 1.73E-01 | 1.66E-01 | 3.30E-02 |
| Carbon dioxide, biogenic | 0.0 | 7.80E-03 | 1.56E-01 |
| Methane, fossil | 3.28E-06 | 3.37E-06 | 5.08E-06 |
| Methane, biogenic | 0.0 | 1.58E-07 | 3.16E-06 |
| Nitrogen oxides | 5.18E-04 | 5.05E-04 | 2.58E-04 |
| Particulates, >2.5 um, and <10um | 2.54E-06 | 2.32E-06 | 0.0 |
| Particulates, <2.5 um | 3.04E-05 | 2.81E-05 | 0.0 |
| Particulates, > 10 um | 1.30E-06 | 1.19E-06 | 0.0 |
| Dinitrogen monoxide | 5.58E-06 | 5.58E-06 | 5.58E-06 |
| Ammonia | 1.00E-06 | 1.00E-06 | 1.00E-06 |
| Sulfur dioxide | 5.51E-06 | 5.25E-06 | 3.10E-07 |
| NMVOC | 1.30E-04 | 1.29E-04 | 1.10E-04 |
| Benzene | 1.81E-06 | 1.80E-06 | 1.61E-06 |
| Toluene | 4.38E-07 | 4.15E-07 | 0.0 |
| Xylene | 1.09E-06 | 1.04E-06 | 9.00E-08 |
| Zinc | 4.13E-10 | 3.74E-08 | 7.40E-07 |
| Copper | 7.01E-08 | 6.36E-08 | 0.0 |
| Cadmium | 4.13E-10 | 3.74E-10 | 0.0 |
| Chromium | 2.06E-09 | 1.87E-09 | 0.0 |
| Chromium VI | 4.13E-12 | 3.74E-12 | 0.0 |
| Nickel | 2.89E-09 | 2.62E-09 | 0.0 |
| Lead | 4.54E-12 | 4.12E-12 | 0.0 |
| Heat, waste | 2.50 | 2.49 | 2.30 |
|  |  |  |  |
| Performance (km/MJ) | 0.42 | 0.42 | 0.42 |

1. Additional file 9: Annual average electricity production for the whole facility from CIGS photovoltaic panels.

1. S10: Data source

**Table 10.1.** Life Cycle Inventory: Data Source and Cumulative Energy Demands (CED).

| **Name in SimaPro** | **Compound** | **Unit** | | **CED 1.09 (MJ)** | **Transport included** |
| --- | --- | --- | --- | --- | --- |
| Ammonium nitrate, as N [[23](#_ENREF_23)]\| market for \| Alloc Def, U | Ammonium nitrate | 1 | kg | 21.1 | yes |
| Nitrogen fertiliser, as N [[23](#_ENREF_23)]\|market for \| Alloc Def, U | DAP | 1 | kg | 18.4 | yes |
| Hexane [[23](#_ENREF_23)]\| market for \| Alloc Def, U | Hexane | 1 | kg | 21.0 | yes |
| Methanol [[23](#_ENREF_23)]\| market for \| Alloc Def, U | Methanol | 1 | kg | 33.3 | yes |
| Sodium hydroxide, without water, in 50% solution state [[23](#_ENREF_23)]\| market for \| Alloc Def, U | NaOH | 1 | kg | 19.8 | yes |
| Sodium methoxide [[23](#_ENREF_23)]\| market for \| Alloc Def, U | CH3NaO | 1 | kg | 36.5 | yes |
| Hydrochloric acid, without water, in 30% solution state {RER}\| market for \| Alloc Def, U | HCl | 1 | kg | 11.7 | yes |
| Heat, district or industrial, natural gas {Europe without Switzerland}I heat production, natural gas, at industrial furnace >100 kWI Alloc Def, U | Natural gas | 1 | MJ | 1.3 | yes |
| Electricity, medium voltage {RER}\| market group for \| Alloc Def, U | Electricity mix Europe | 1 | kWh | 11.3 | yes |
| Steel, low-alloyed [[23](#_ENREF_23)]\| market for \| Alloc Def, U | Machinery | 1 | kg | 19.8 | yes |
| PVC pipe E | PVC pipes | 1 | kg | 67.0 | no |
| HDPE pipes E | HDPE pipes | 1 | kg | 85.5 | no |
| E-glass, at plant/kg/RNA | Low E | 1 | kg | 23.5 | no |
| Steel, low-alloyed [[23](#_ENREF_23)]\| market for \| Alloc Def, U | Steel | 1 | kg | 19.8 | yes |
| Aluminium alloy, AlMg3 [[23](#_ENREF_23)]\| market for \| Alloc Def, U | Aluminium | 1 | kg | 156.8 | yes |
| Concrete block [[23](#_ENREF_23)]\| market for \| Alloc Def, U | Concrete | 1 | kg | 0.88 | yes |
| Waste Polyvinylchloride {Europe without Switzerland}\| market for waste polyvinylchloride \| Alloc Rec, U | PVC to recycling | 1 | kg | 2.3 | yes |
| Waste Polyethylene {Europe without Switzerland}\| market for waste polyethylene \| Alloc Rec, U | HDPE to recycling | 1 | kg | 0.33 | yes |
| Waste reinforced steel {RoW}\| market for waste reinforced steel \| Alloc Rec, U | Steel to recycling | 1 | kg | 1.05 | yes |
| Waste aluminium [[23](#_ENREF_23)]\| market for\| Alloc Rec, U | Aluminium to recycling | 1 | kg | 0.71 | yes |
| Waste glass sheet {Europe without Switzerland}\| market for waste glass sheet \| Alloc Rec, U | Glass (low E) to recycling | 1 | kg | 0.23 | yes |
| Waste concrete {Europe without Switzerland}\| market for waste concrete \| Alloc Rec, U | Concrete to landfill | 1 | kg | 0.22 | yes |
| Photovoltaic panel, CIS [[23](#_ENREF_23)]\| market for \| Alloc Def, U | PV panel | 1 | m2 | 2107.5 | yes |
| Water, river, FR | Water natural source | 1 | m3 | 0 | -- |
| Ammonia | Ammonia, emissions | 1 | kg | 0 | -- |
| Dinitrogen monoxide | Dinitrogen monoxide, emissions | 1 | kg | 0 | -- |
| Carbon dioxide, fossil | Carbon dioxide, fossil emissions | 1 | kg | 0 | -- |
| Hexane | Hexane emissions | 1 | kg | 0 | -- |
| PVC film E | PVC (liner) | 1 | kg | 70.0 | no |
| *Transport, passenger car, medium size, Rape seed methyl ester, EURO 3 {RER}\| transport, passenger car, medium size, diesel, EURO 3 \| Alloc Def, U* | Biodiesel combustion (only use) | 1 | km | 0 | -- |
| CO_2_ fossil (from flue gas) | Carbon dioxide, fossil Inputs | 1 | kg | 0 | -- |

1. S11: biomass and biodiesel productivity for different coverture of photovoltaic panels


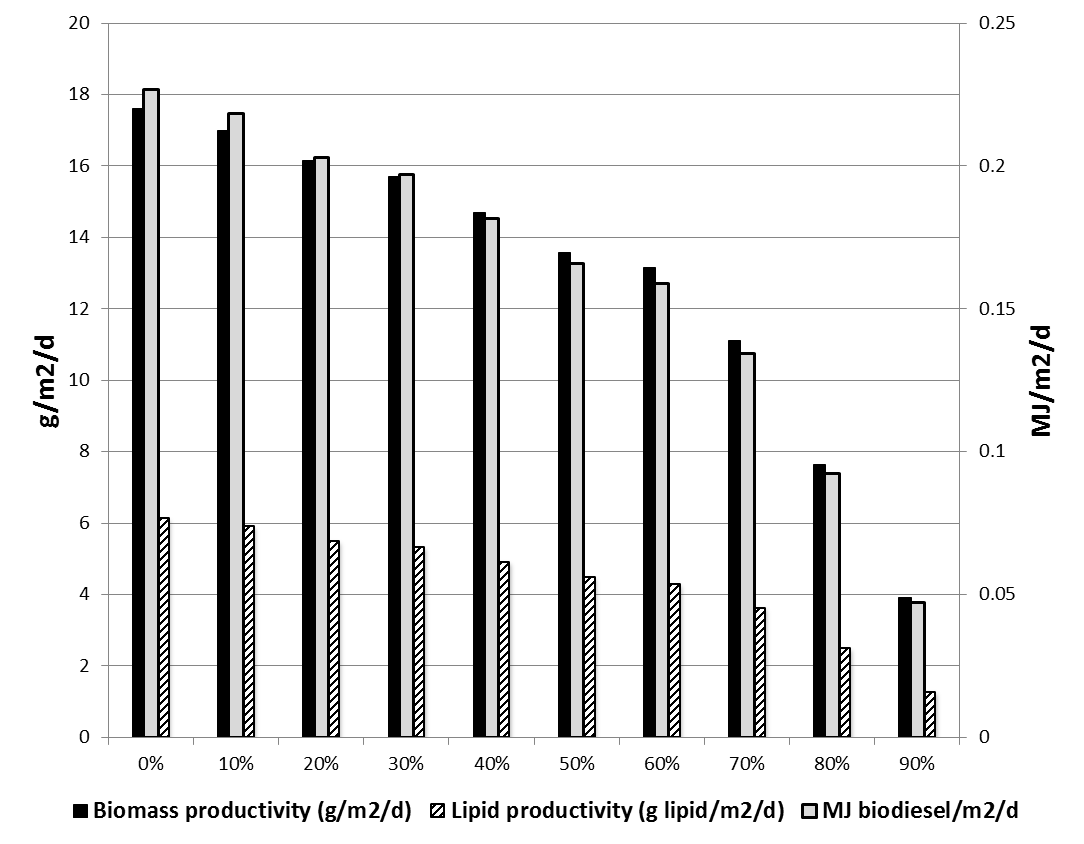
(a)


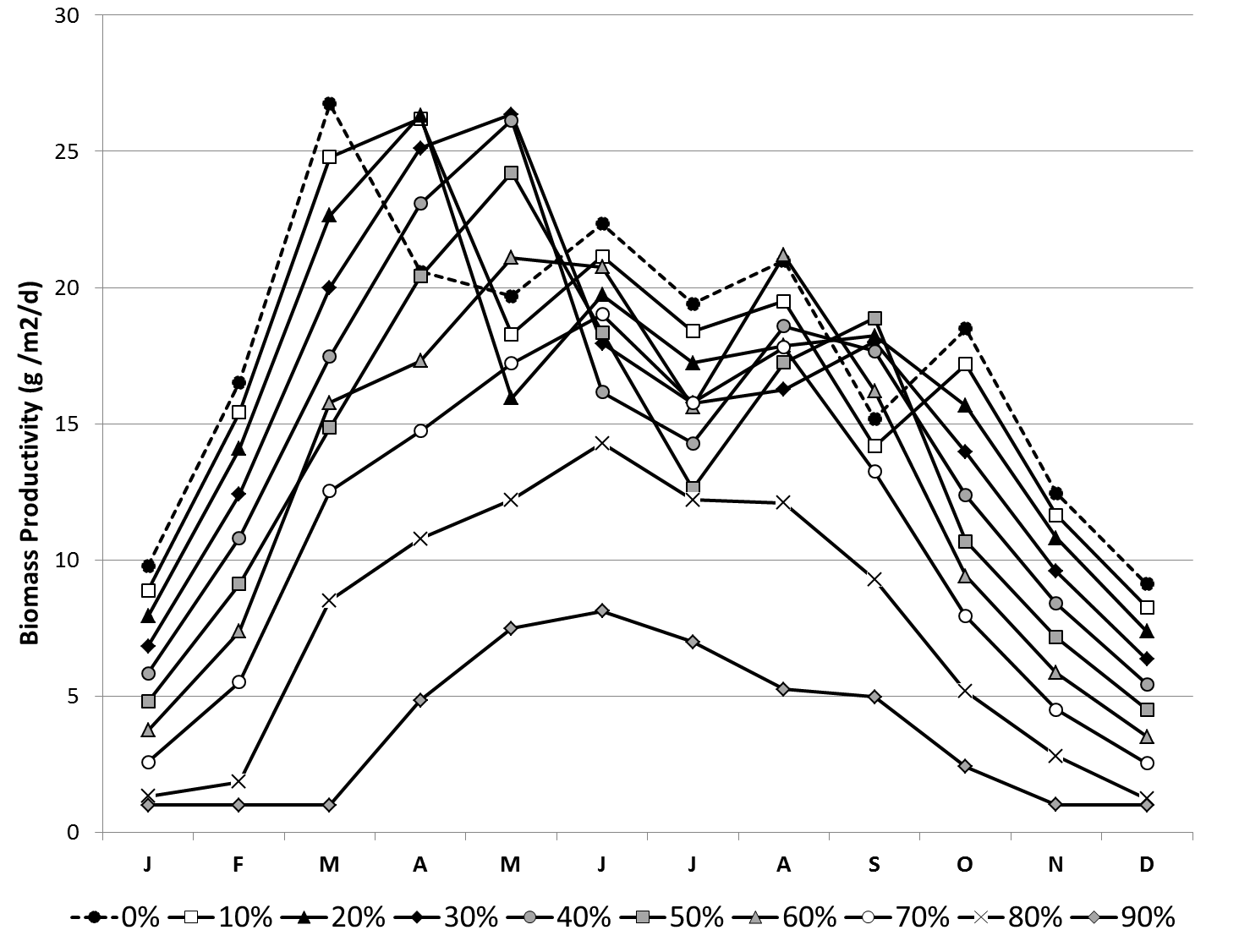
(b)


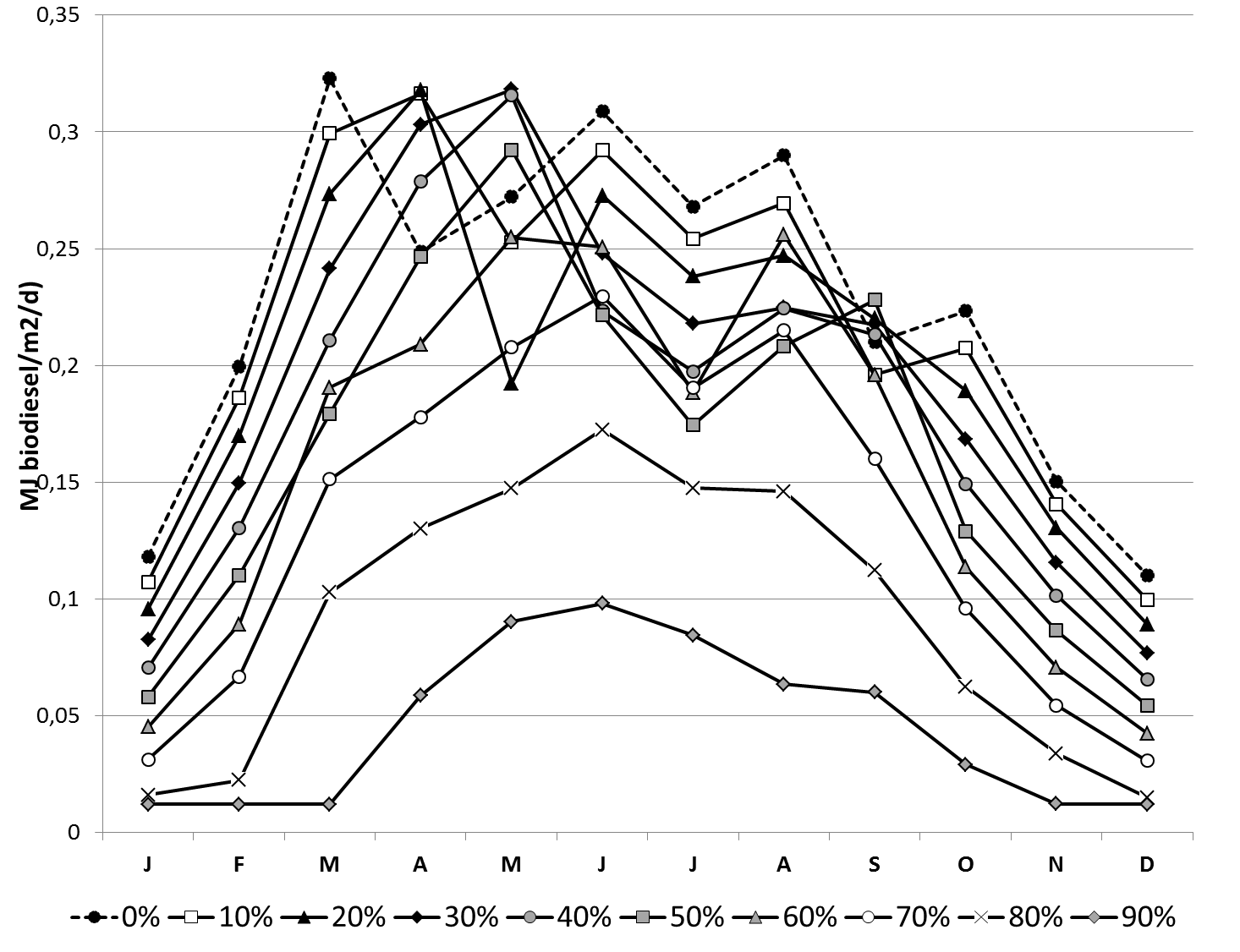
(c)

**Figure 11.1.** (a) Annual average biomass and biodiesel productivity, (b) Monthly average biomass productivity, (c) Monthly average biomass productivity, for different coverture of PV panels

1. S12: CED (renewable + non-renewable) and energy production associated with production of 1 MJ biodiesel.

1. S13: Monthly variation of NER and FER


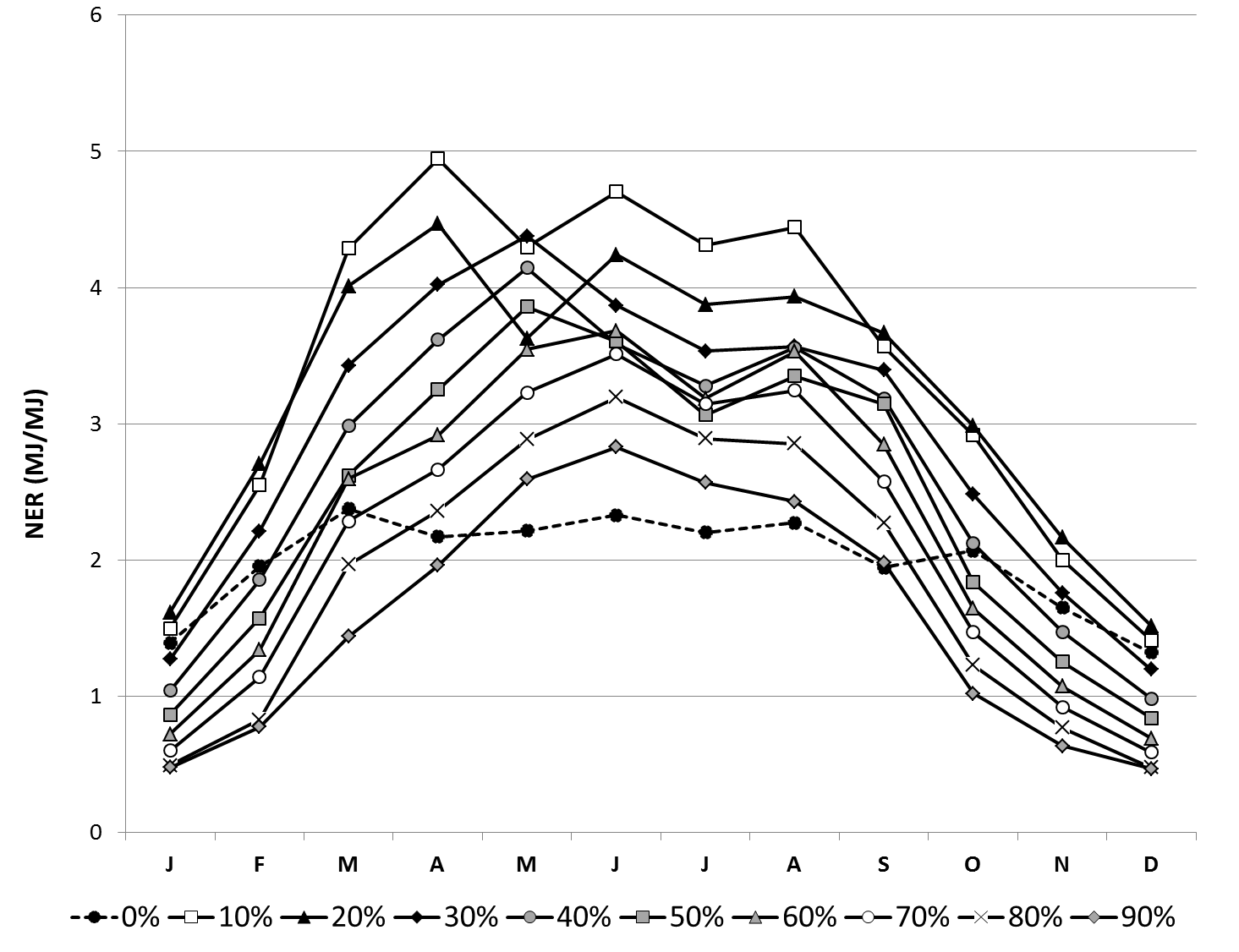
(a)


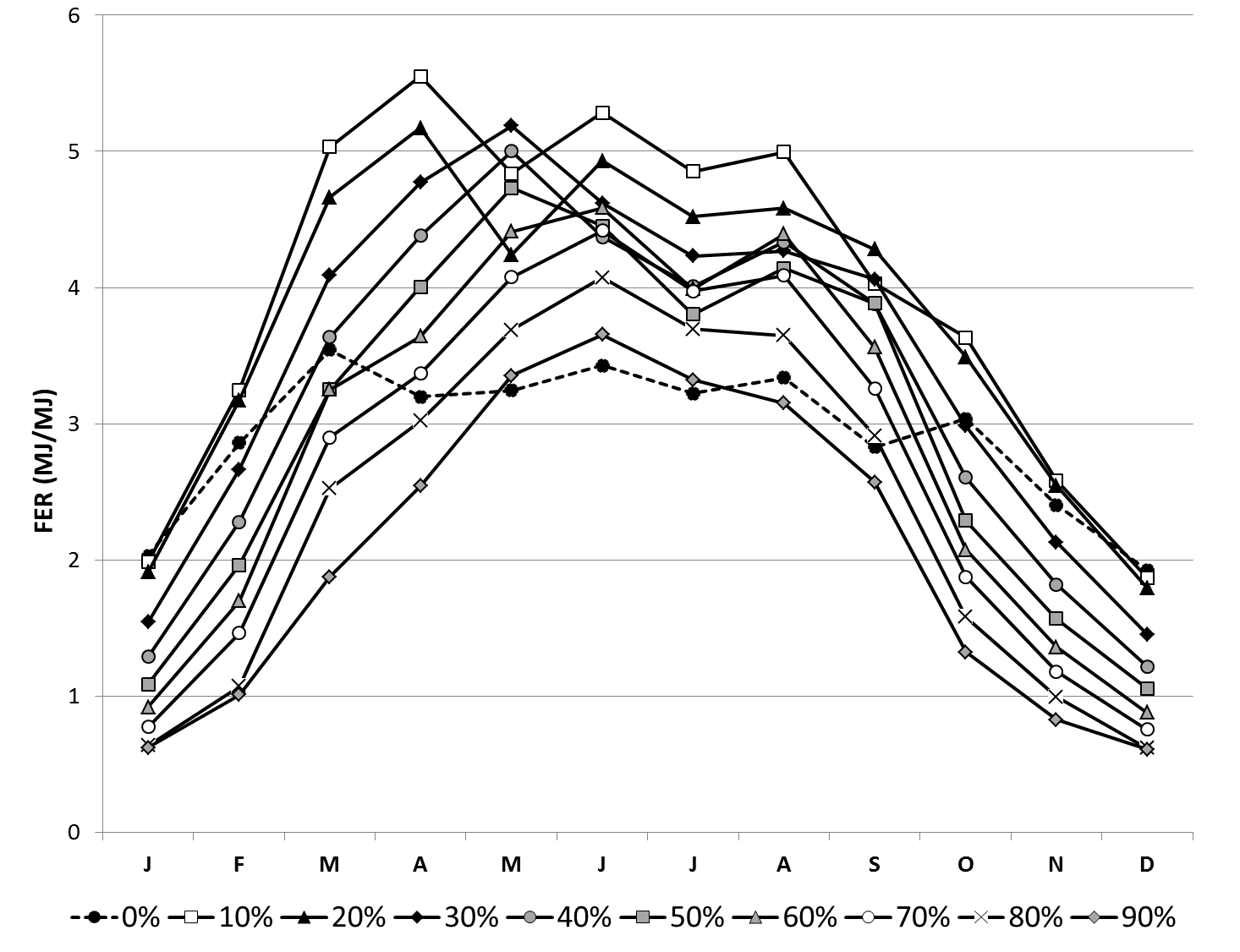
(b)

**Figure 13.1.** Monthly average NER (a) and FER (b) for different coverture of PV panels

1. S14: LCA for biodiesel from Rapeseed, Palm tree, Soybean and conventional diesel.

The references cases are obtained from Ecoinvent database for biodiesel and conventional fossil diesel (**Table 14.1**), complying with the same limits of the system and allocation of our study. The biodiesel reference scenarios are soybean diesel (US), palm tree diesel (Malaysia) and rapeseed diesel (European average).

**Table 14.1.** Compounds used in vegetable biodiesel and fossil diesel obtained from Ecoinvent database

| **Compound** | **Name in SimaPro** |
| --- | --- |
| Rape Biodiesel | 1 kg Vegetable oil methyl ester {Europe without Switzerland}\| esterification of rape oil \| Alloc Def, U |
| Soybean Biodiesel | 1 kg Vegetable oil methyl ester {US}\| esterification of soybean oil \| Alloc Def, U |
| Palm tree Biodiesel | 1 kg Vegetable oil methyl ester {M.Y}\| esterification of palm oil \| Alloc Def, U |
| Conventional Diesel | Diesel, low-sulfur {RoW}\| production \| Alloc Def, U |

- 1. Vegetable biodiesel

Life cycle phases for vegetable biodiesel are the oil mill and esterification, obtaining meal (and kernel oil in the case of palm tree) and glycerin, respectively (Figure 14.1).

**Figure 14.1**. Life cycle processes in vegetable biodiesel.

The energetic allocation values for oil mill and esterification phases where the impacts have to be split between oil and meal (oil mill) and biodiesel and glycerine (esterification) are shown in the tables below (**Table 14.2**):

**Table 14.2** Allocation factors in oil mill and esterification phases

| Oil mill coproducts SOYBEAN | Mass flow  (kg coproduct/kg seed) [[24](#_ENREF_24)] | **Mass allocation (%)** | Energy content (MJ/kg) [[25](#_ENREF_25)] | Energy content (MJ/kg seed) | **Energetic**  **allocation (%)** |
| --- | --- | --- | --- | --- | --- |
| Soybean oil | 0.19 | 19.9 | 37.2 | 7.16 | 35.3 |
| Soybean meal | 0.77 | 80.1 | 17 | 13.14 | 64.7 |
| **total** | **0.97** | **100** |  | **20.30** | **100** |
| Oil mill coproducts RAPESEED | Mass flow  (kg coproduct/kg seed) [[25](#_ENREF_25)] | **Mass allocation (%)** | Energy content (MJ/kg) [[26](#_ENREF_26)] | Energy content (MJ/kg seed) | **Energetic**  **allocation (%)** |
| Rape oil | 0.42 | 42.6 | 38.4 | 16.08 | 65.1 |
| Rape meal | 0.56 | 57.4 | 15.3 | 8.63 | 34.9 |
| **total** | **0.98** | **100** |  | **24.71** | **100** |
| Oil mill coproducts PALMTREE | Mass flow  (kg coproduct/kg seed) [[25](#_ENREF_25)] | **Mass allocation (%)** | Energy content (MJ/kg) [[27](#_ENREF_27)] | Energy content (MJ/kg seed) | **Energetic**  **allocation (%)** |
| Palm oil | 0.200 | 79.5 | 37 | 7.39 | 88.9 |
| Palm meal | 0.028 | 11.0 | 18.7 | 0.52 | 6.2 |
| Palm Kernel oil | 0.024 | 9.5 | 17 | 0.41 | 4.9 |
| **total** | **0.25** | **100** |  | **8.31** | **100** |
| Esterification coproducts SOYBEAN | Mass flow  (kg coproduct/kg oil) [[28](#_ENREF_28)] | **Mass allocation (%)** | Energy content (MJ/kg) [[20](#_ENREF_20)] | Energy content (MJ/kg oil) | **Energetic**  **allocation (%)** |
| Biodiesel | 0.90 | 82.4 | 37.2 | 33.54 | 92.0 |
| Glycerine | 0.19 | 17.6 | 15.2 | 2.92 | 8.0 |
| **total** | **1.09** | **100.00** |  | **36.46** | **100** |
| Esterification coproducts RAPESEED | Mass flow  (kg coproduct/kg oil) [[29](#_ENREF_29)] | **Mass allocation (%)** | Energy content (MJ/kg) [[29](#_ENREF_29)] | Energy content (MJ/kg oil) | **Energetic**  **allocation (%)** |
| Biodiesel | 663.0 | 90.5 | 37.0 | 24531.0 | 95.6 |
| Glycerine | 70.0 | 9.5 | 16.0 | 1120.0 | 4.4 |
| **total** | **733.0** | **100** |  | **25651.0** | **100** |
| Esterification coproducts PALMTREE | Mass flow  (kg coproduct/L oil) [[27](#_ENREF_27)] | **Mass allocation (%)** | Energy content (MJ/kg) [[27](#_ENREF_27)] | Energy content (MJ/kg oil) | **Energetic**  **allocation (%)** |
| Biodiesel | 0.87 | 95.08 | 38.07 | 33.12 | 97.5 |
| Glycerine | 0.05 | 4.92 | 19.0 | 0.86 | 2.5 |
| **total** | **0.92** | **100** |  | **33.9** | **100** |

As calculation example, **Table 14.3** show the energetic allocation factors to the single phases and the impacts values of climate change category obtained in the each case. Their values reflect the fact that in the allocation process each upstream phase benefits from all the downstream coproducts. The rest of impacts results for each case are shown in **Additional file 17**.

**Table 14.3** Single phases not allocated and energy allocated impacts of climate change expressed as kg CO_2_ eq·MJ^-1^ biodiesel for rapeseed, palm tree and soybean.

|  | **Rapeseed** | | | **Palm tree** | | | **Soybean** | | |
| --- | --- | --- | --- | --- | --- | --- | --- | --- | --- |
| Phases | Not allocated | Allocation factor | Allocated | Not allocated | Allocation factor | Allocated | Not allocated | Allocation factor | Allocated |
| Oil mill | 0.073 | 0.65 | 0.047 | 0.073 | 0.89 | 0.065 | 0.055 | 0.35 | 0.019 |
| Esterification | 0.0042 | 0.96 | 0.0040 | 0.0045 | 0.97 | 0.0044 | 0.006 | 0.92 | 0.0056 |
| Combustion | 0.0146 | 1.00 | 0.015 | 0.015 | 1.00 | 0.015 | 0.015 | 1.00 | 0.016 |
| **TOTAL** | **0.092** |  | **0.066** | **0.092** |  | **0.084** | **0.076** |  | **0.040** |

- 1. Conventional diesel

Energetic allocation values for fossil oil refinery are shown in the table below (**Table 14.4**):

**Table 14.4** Allocation factors in refinery.

| Refinery coproducts  FOSSIL DIESEL | Mass flow  (kg coproduct/kg refined mix) [[30](#_ENREF_30)] | **Mass allocation (%)** | Energy content (MJ/kg) | **Energetic**  **allocation (%)** |
| --- | --- | --- | --- | --- |
| Diesel | 0.23 | 23.35 | 42.6 | 23.42 |
| Liquefied petroleum gas | 0.03 | 2.81 | 49.0 | 3.25 |
| Gasoline | 0.39 | 38.96 | 44.0 | 40.36 |
| Residual fuel oil | 0.04 | 4.50 | 39.5 | 4.18 |
| Bitumen | 0.04 | 3.60 | 26.1 | 2.22 |
| Kerosene | 0.09 | 8.98 | 46.5 | 9.83 |
| Petroleum coke | 0.06 | 5.84 | 29.5 | 4.06 |
| Refinery gas | 0.07 | 6.89 | 46.9 | 7.61 |
| Petroleum refining coproduct, unspecified | 0.05 | 5.06 | 42.6 | 5.08 |
| **total** | **0.99** | **100** |  | **100** |

As calculation example, **Table 14.5** show the energetic allocation factors to the single phases and the impacts values of climate change category obtained in the fossil diesel. The rest of impacts category results are shown in **Additional file 17**.

**Table 14.5** Single phases not allocated and energy allocated impacts of climate change expressed as kg CO_2_ eq·MJ^-1^ diesel.

|  | **Diesel** | | |
| --- | --- | --- | --- |
| Phases | *Not allocated* | *Allocation factor* | *Allocated* |
| Refinery | 0.058 | 0.23 | 0.014 |
| Combustion | 0.080 | 1.00 | 0.080 |
| **TOTAL** | **0.138** |  | **0.094** |

- 1. Substitution method for vegetable biodiesel and conventional diesel

Energy allocation did not make possible to highlights the importance of the valorization of coproducts on sustainability assessment. The real uses of coproducts in the supply chain in the substitution method allow the valorization of coproducts (as amendments of environmental impacts). The saved emissions due to the substitution of conventional products by the coproducts are reported in **Table 14.6** for climate change category. Negative values sign the fact that they represent an impact reduction. The rest of impacts results for each case are shown in **Additional file 19**. As example, **Table 14.7** shows the results obtained for each allocation method in the final climate change results and highlights the importance in the choice of allocation method.

**Table 14.6** Vegetable biodiesel and fossil diesel impact for climate change (expressed as kg CO_2_ eq·MJ^-1^ biodiesel), applying substitution method.

|  | **Rapeseed** | **Palmtree** | **Soybean** | **Diesel** |
| --- | --- | --- | --- | --- |
| (Bio)diesel chain | 0.092 | 0.092 | 0.076 | 0.138 |
| Substitution of rape meal | -0.054 | - | - | - |
| Substitution of palm kernel meal | - | -0.059 | - | - |
| Substitution of palm kernel oil | - | -0.058 | - | - |
| Substitution of soybean meal | - | - | -0.022 | - |
| Substitution glycerine | -0.011 | -0.013 | -0.006 |  |
| Substitution of LPG | - | - | - | -0.001 |
| Substitution of gasoline | - | - | - | -0.022 |
| Substitution of residual fuel oil | - | - | - | -0.002 |
| Substitution of bitumen | - | - | - | -0.002 |
| Substitution of kerosene | - | - | - | -0.005 |
| Substitution of petroleum coke | - | - | - | -0.003 |
| Substitution of refinery gas | - | - | - | 0.000 |
| Substitution of petroleum refining coproduct (unspecified) | - | - | - | -0.003 |
| **TOTAL** | **0.0266** | **-0.0392** | **0.0471** | **0.1003** |

**Table 14.7** Comparison of climate change results between energetic based allocation and substitution.

| **kg CO_2_ eq·MJ^-1^ biodiesel** | **Rapeseed** | **Palmtree** | **Soybean** | **Diesel conventional** |
| --- | --- | --- | --- | --- |
| Energetic allocation | 0.066 | 0.084 | 0.040 | 0.094 |
| Substitution | 0.027 | -0.039 | 0.047 | 0.100 |

1. S15: Endpoint impact assessment associated with production of 1 MJ biodiesel.

1. S16: Global warming impact of 1 MJ biodiesel by case study (annual average).

1. S17: Midpoint categories results using energetic allocation method

|  |
| --- |
|  |
|  |

|  |
| --- |
|  |
|  |
|  |

|  |
| --- |
|  |
|  |
|  |

|  |
| --- |
|  |
|  |
|  |
|  |
|  |


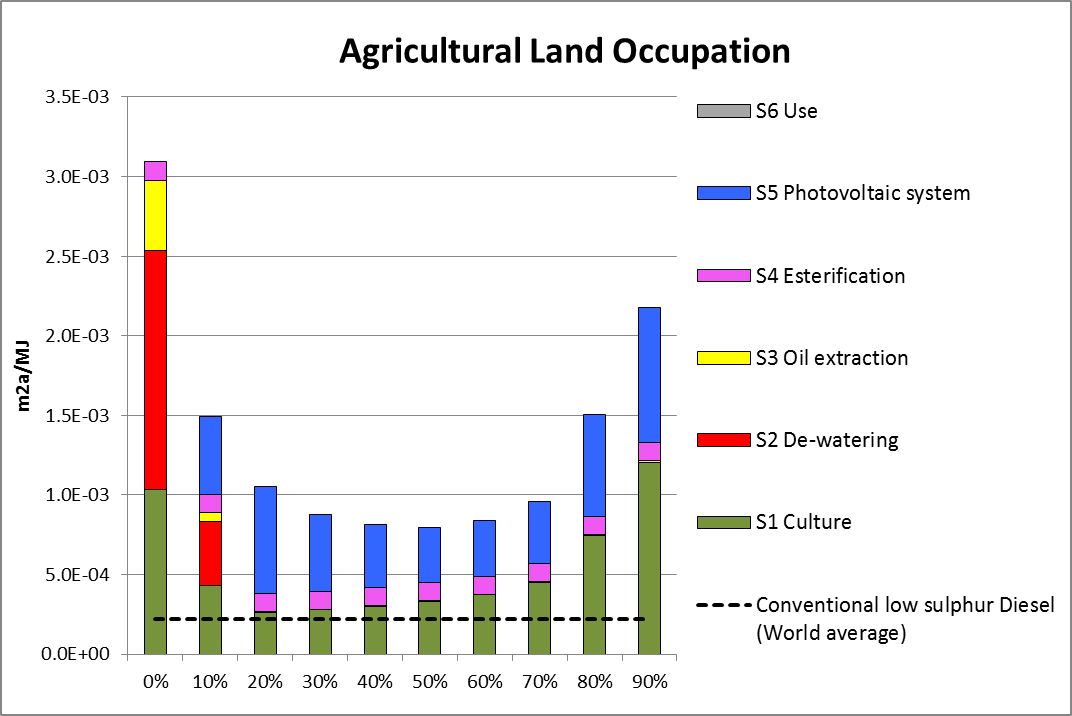


| 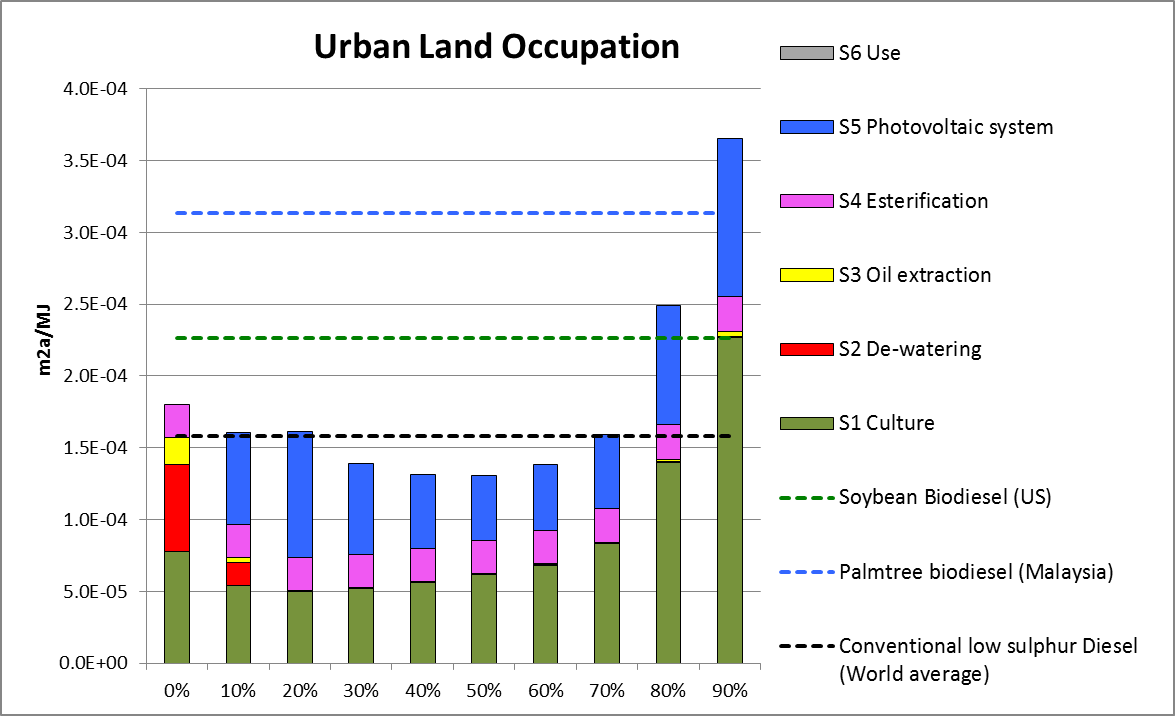   |
| --- |
|  |
|  |
|  |

1. S18: Monthly GHG emissions for different coverture of PV panels


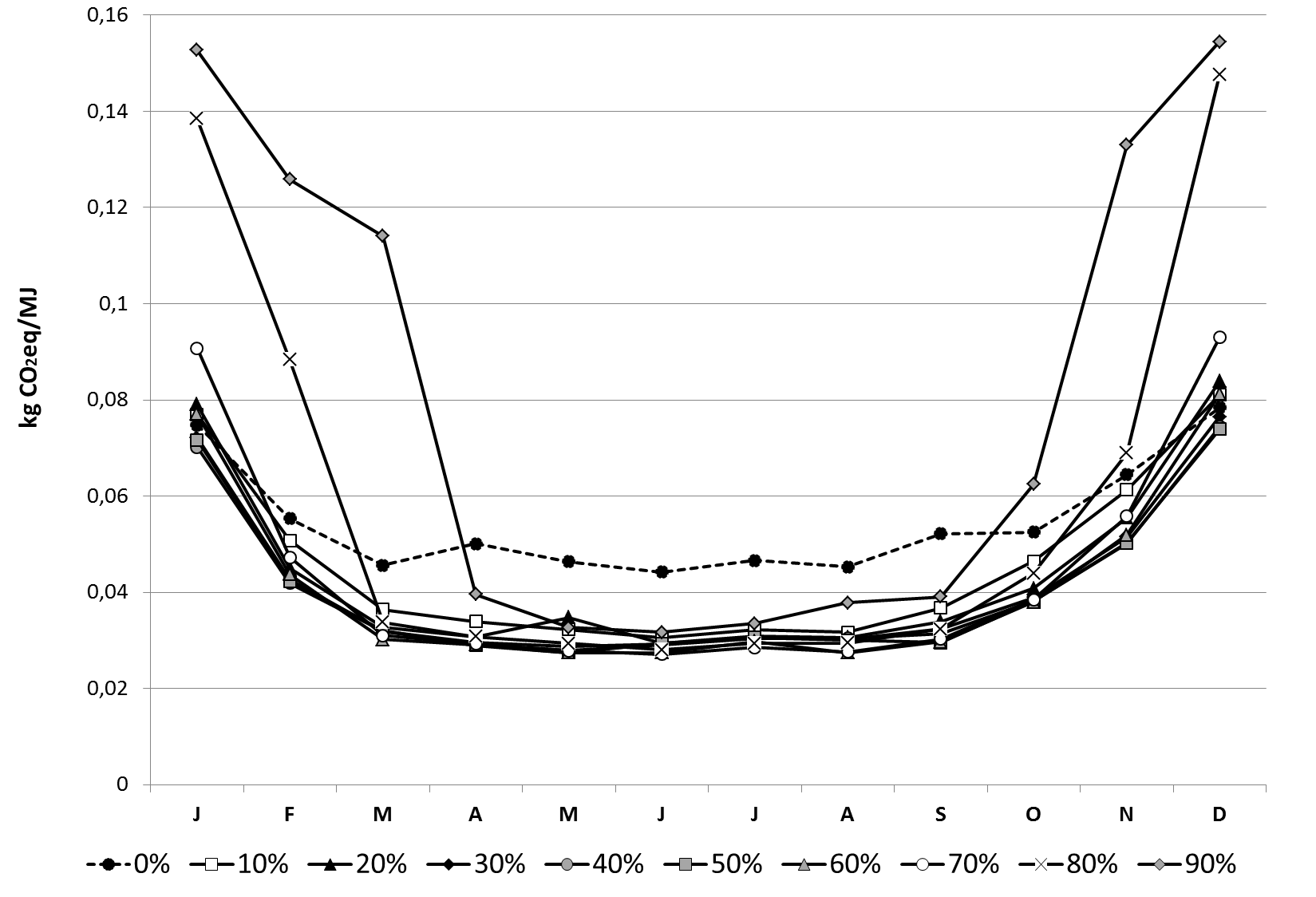


**Figure 18.1.** Monthly GHG emissions for different coverture of PV panels

1. S19: Endpoint and Midpoint categories results using substitution as allocation method


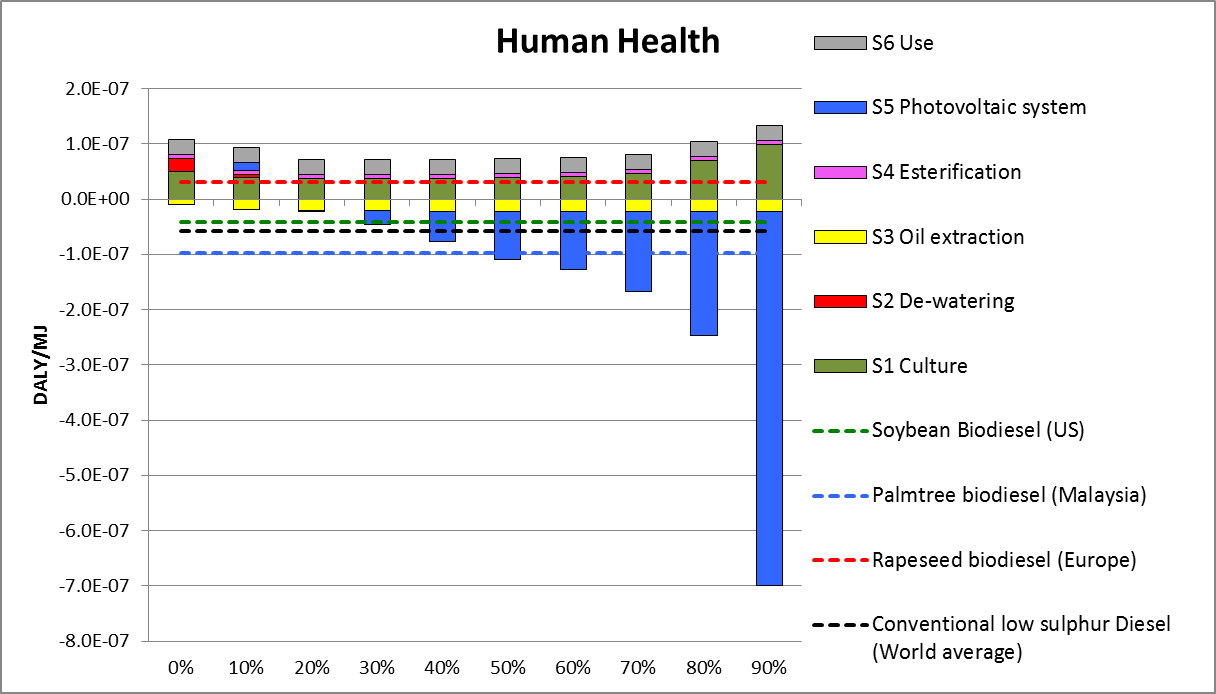


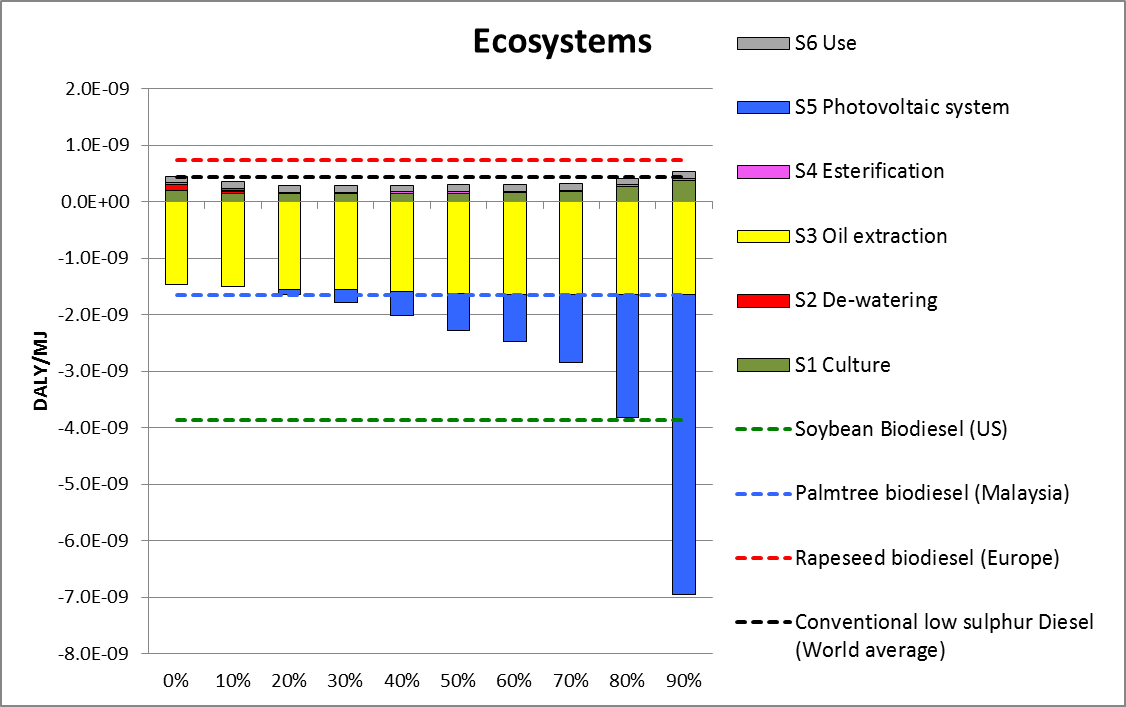


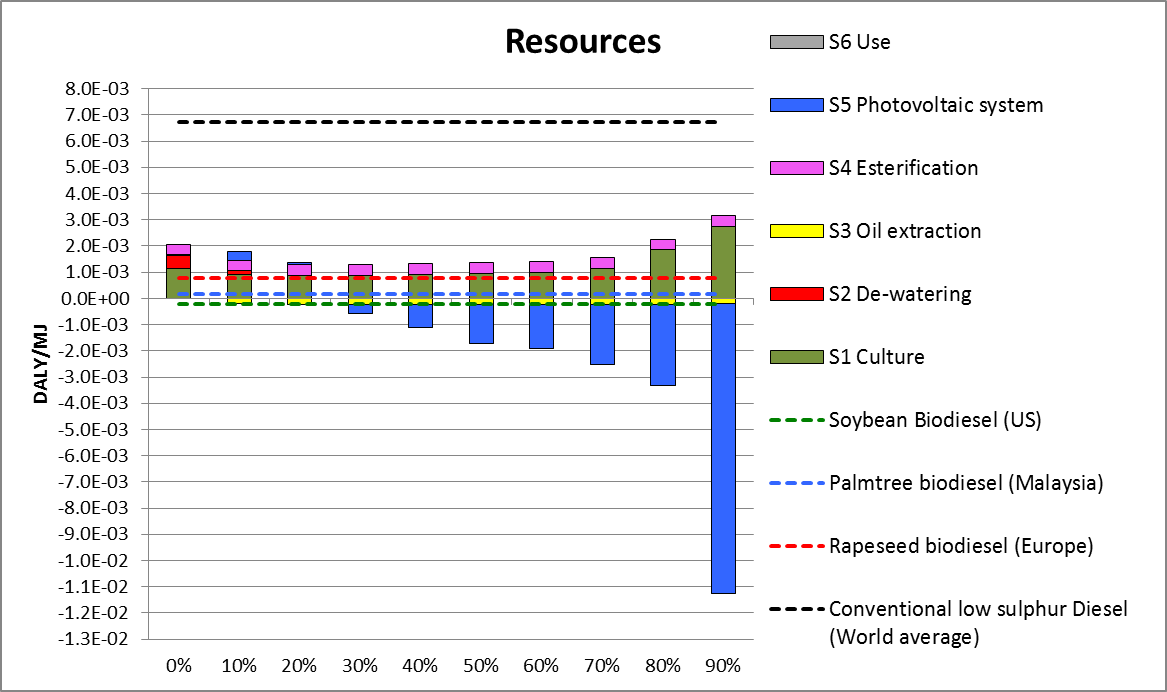


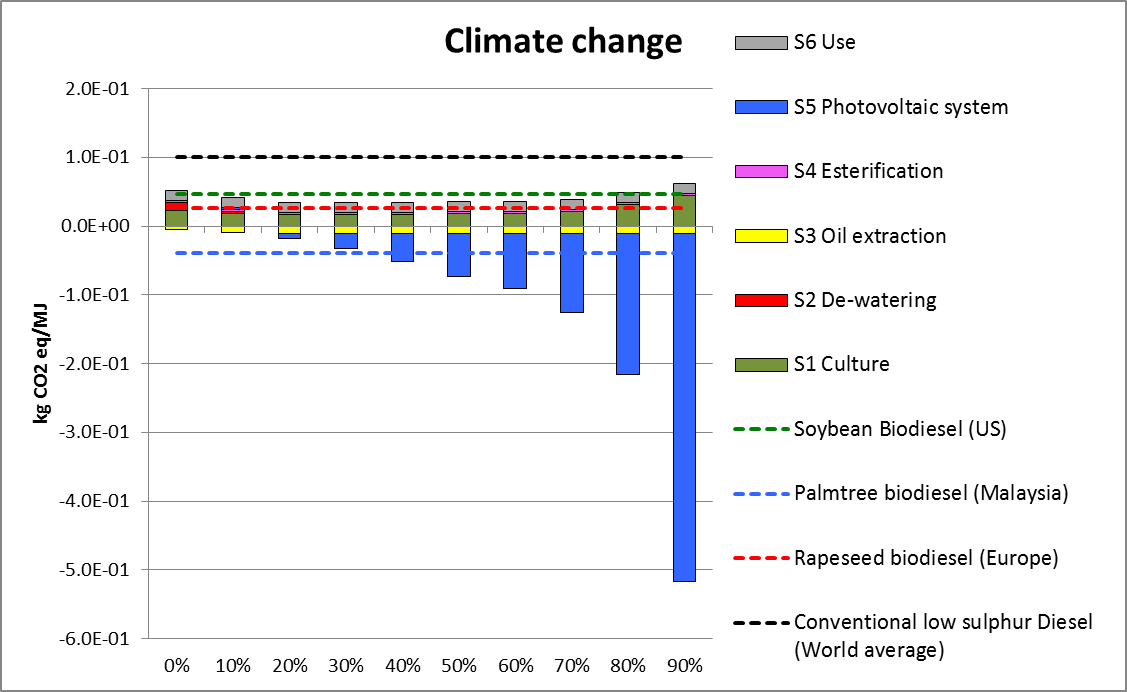


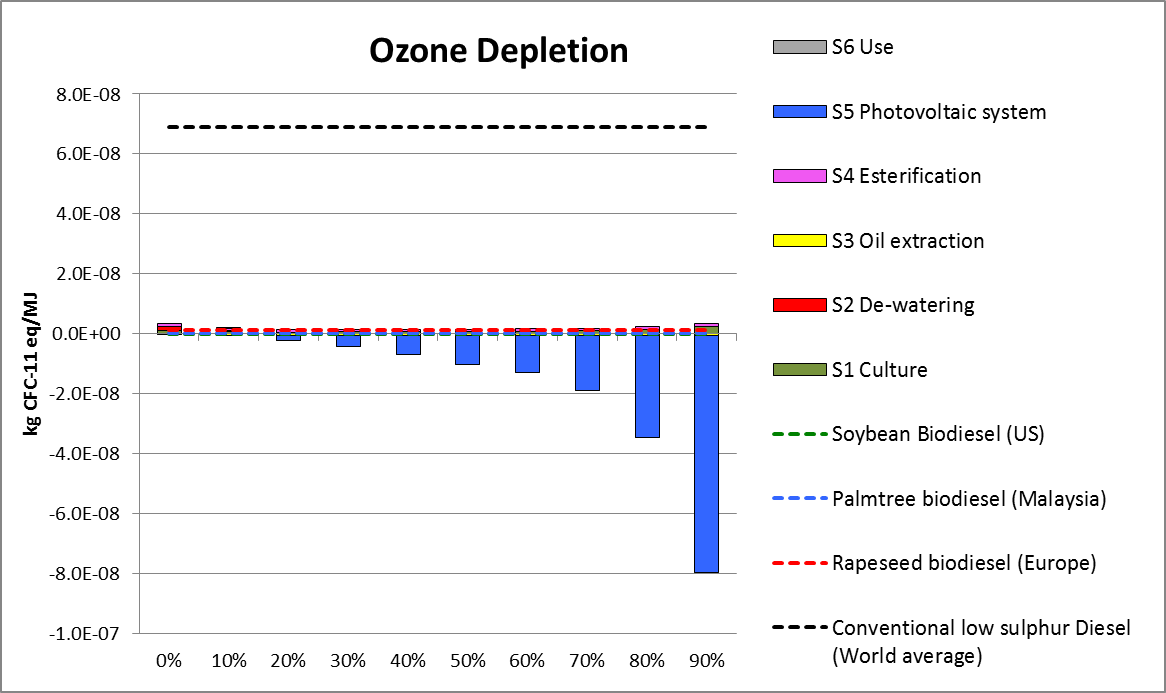


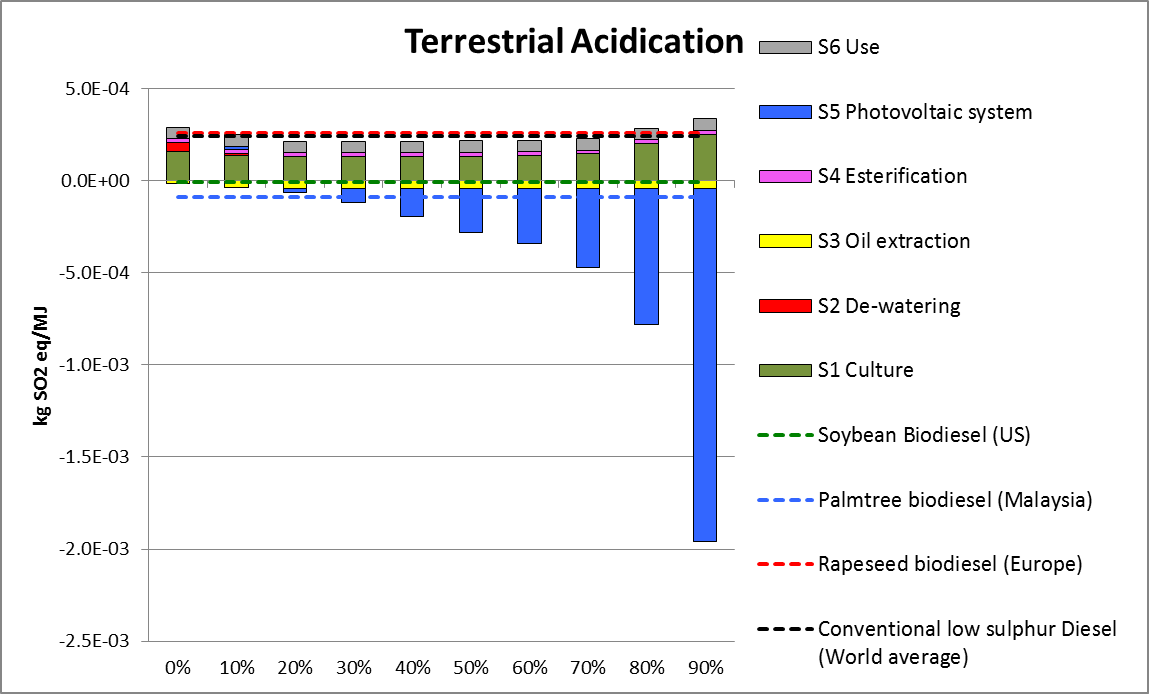


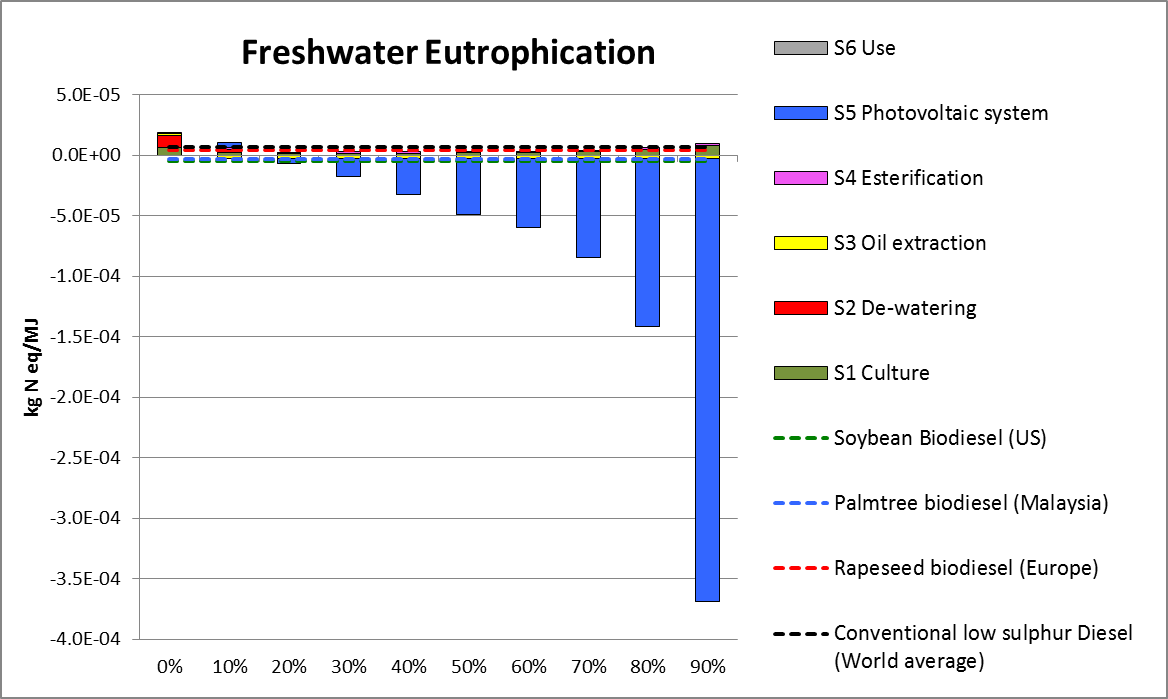


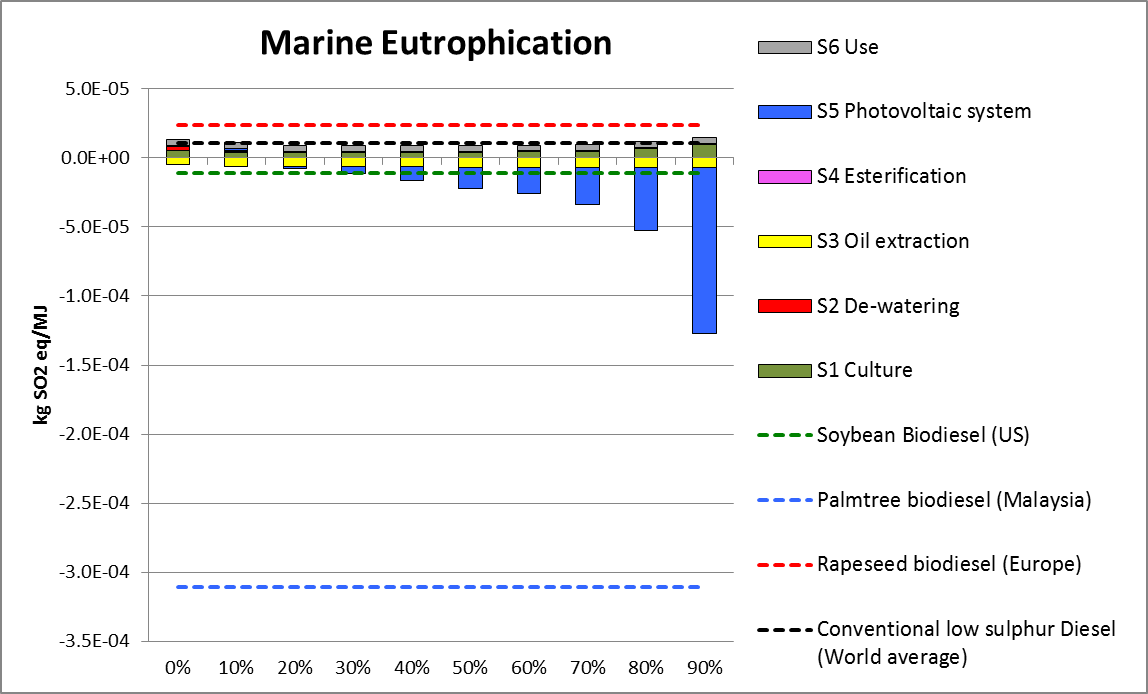


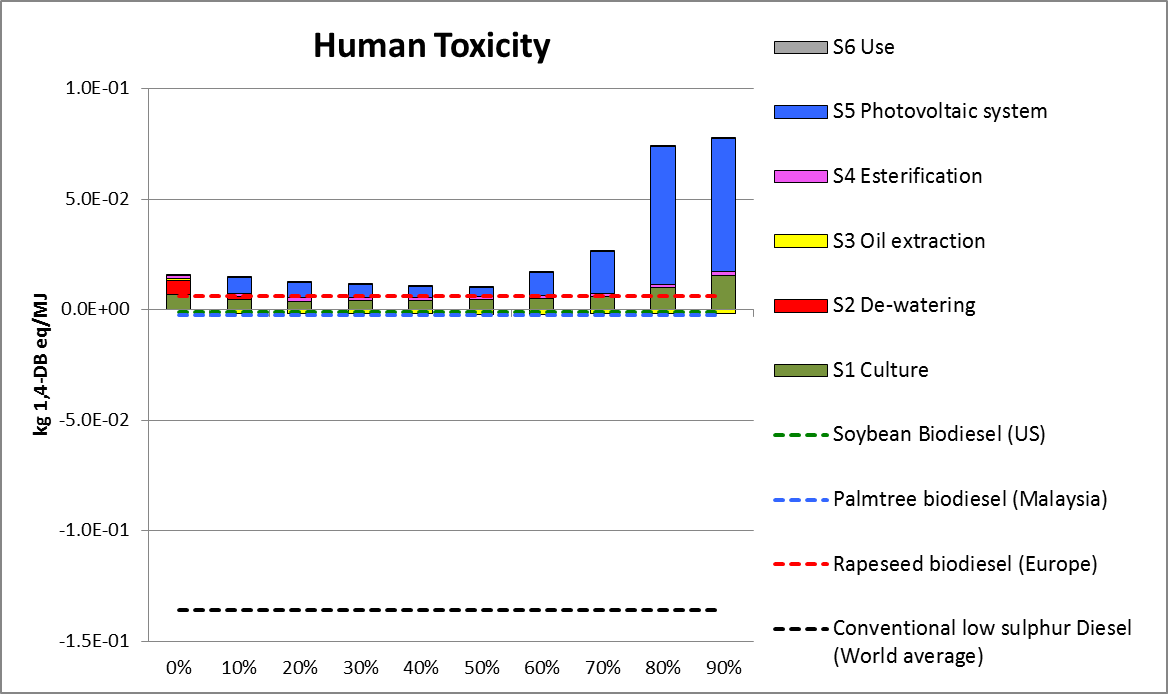


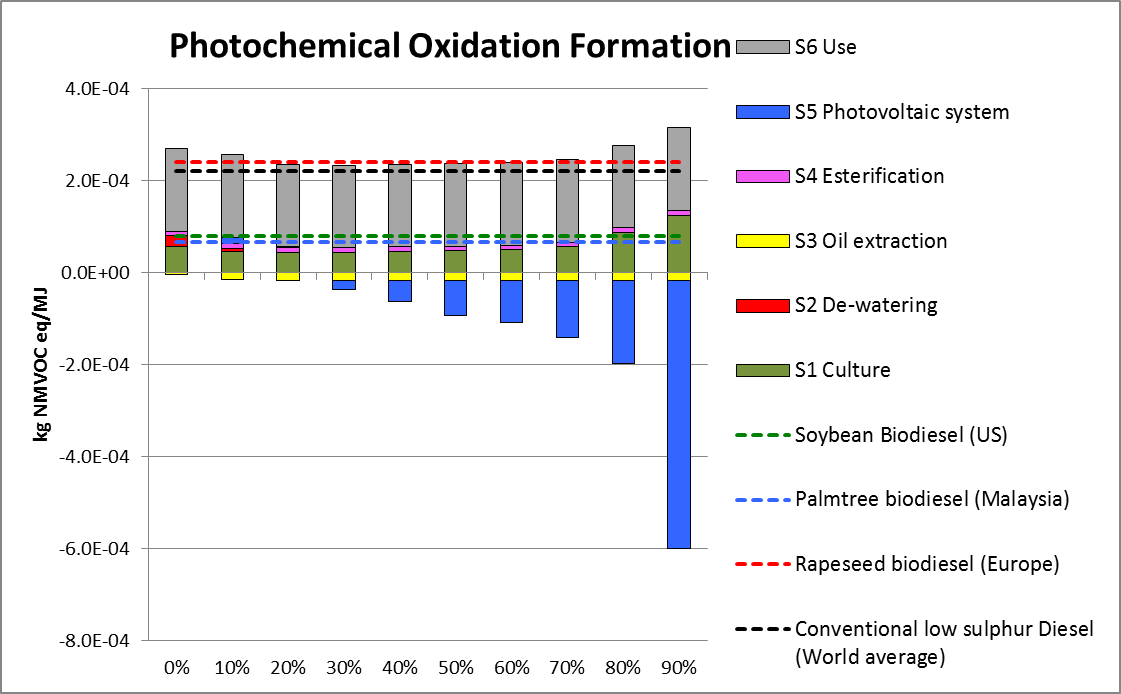


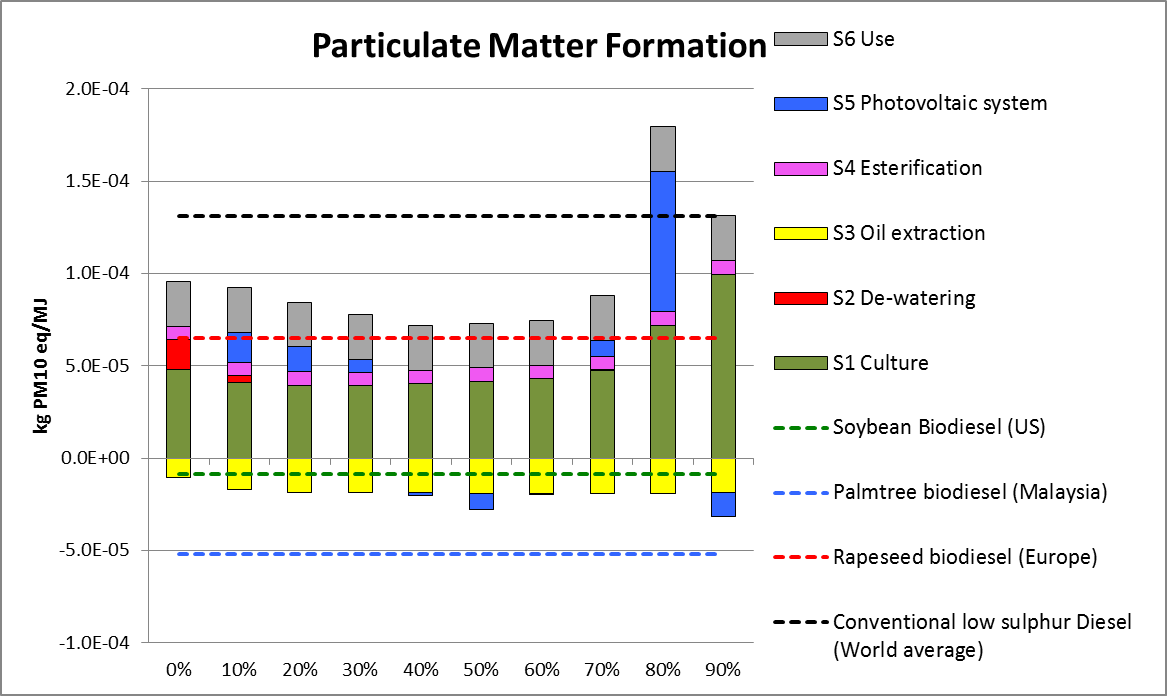


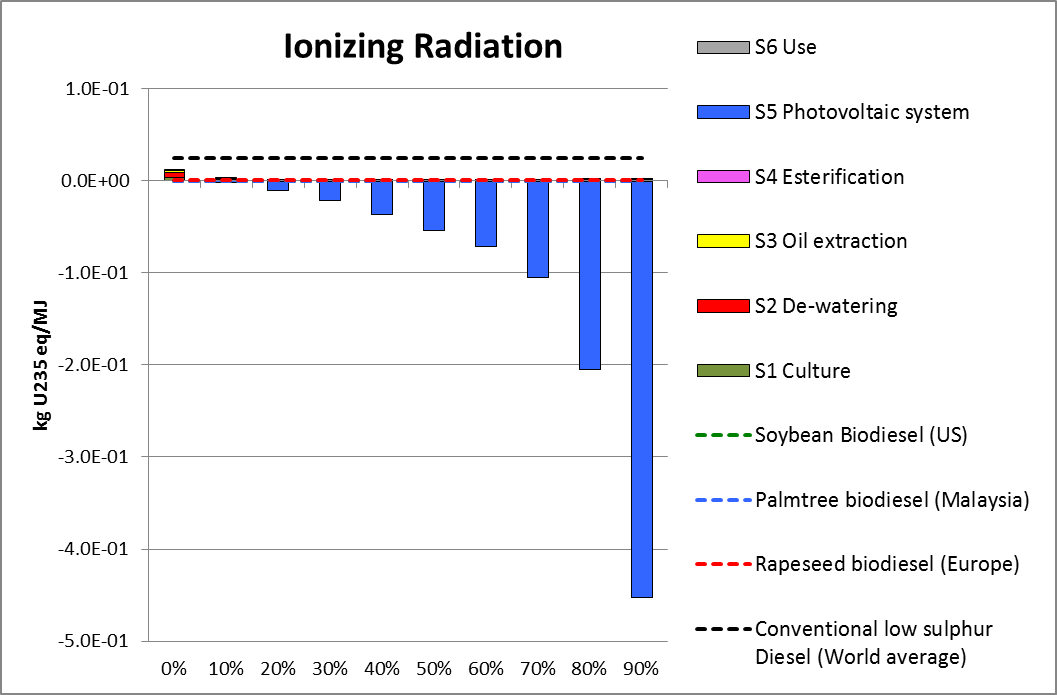


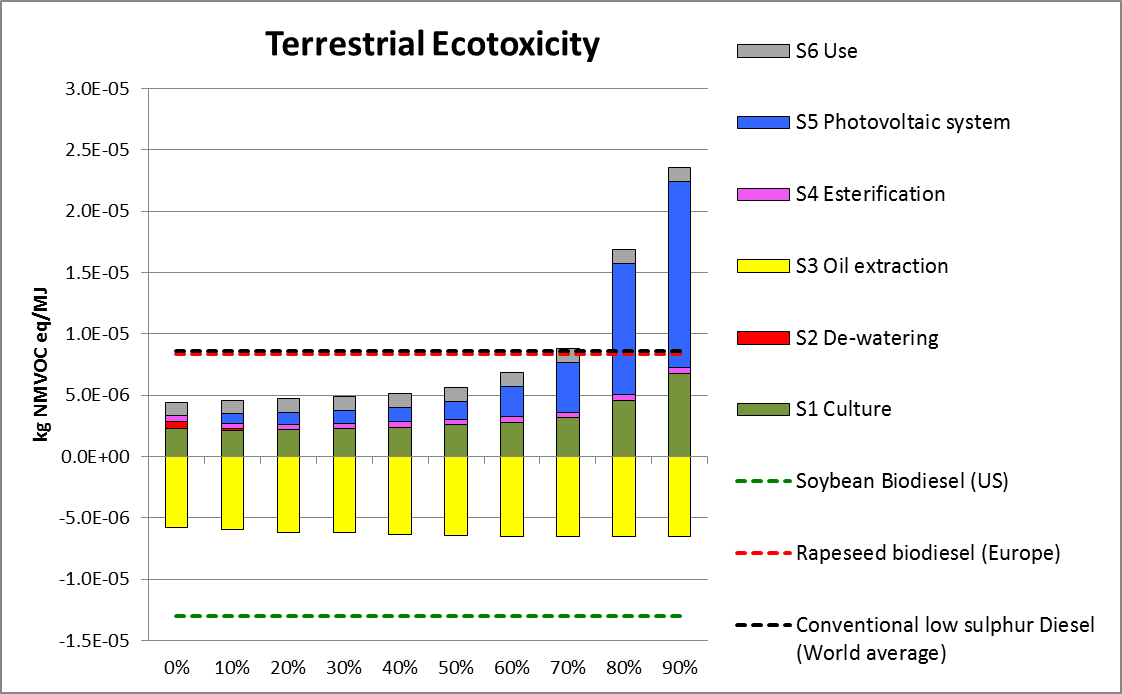


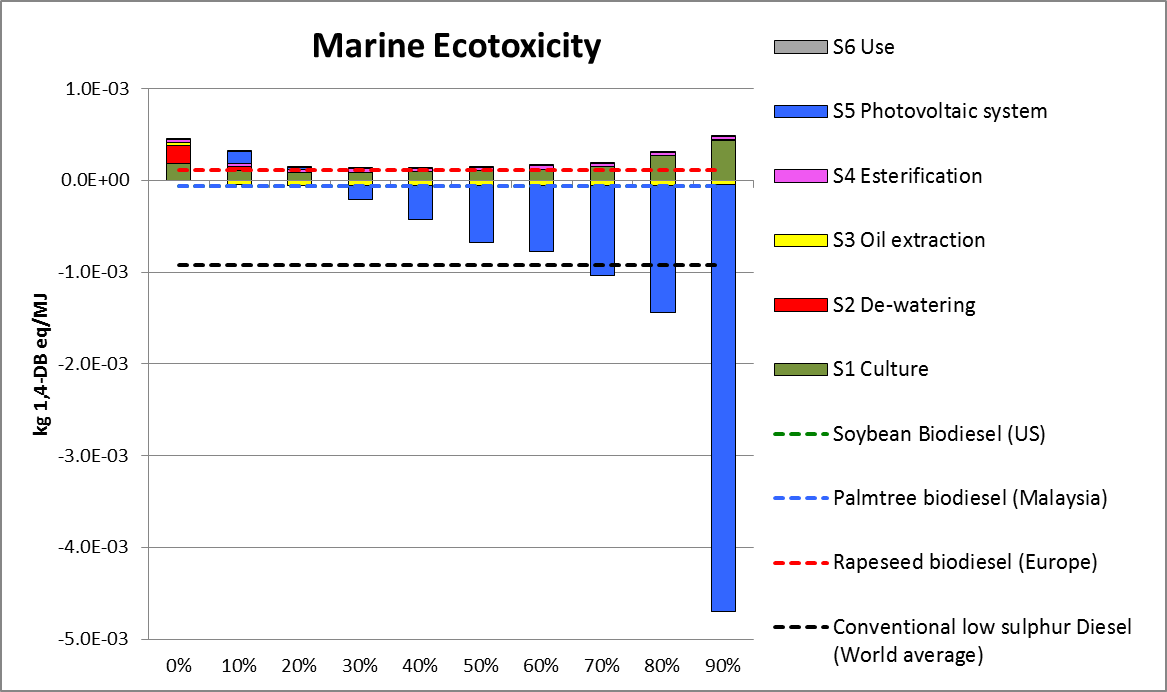


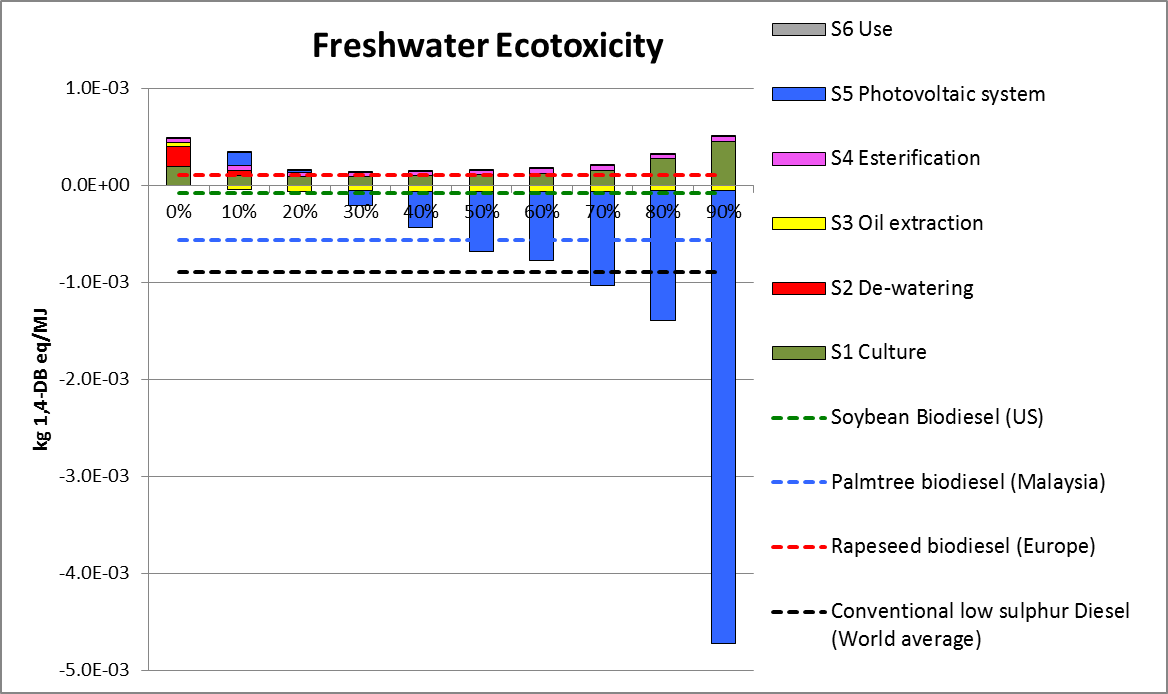


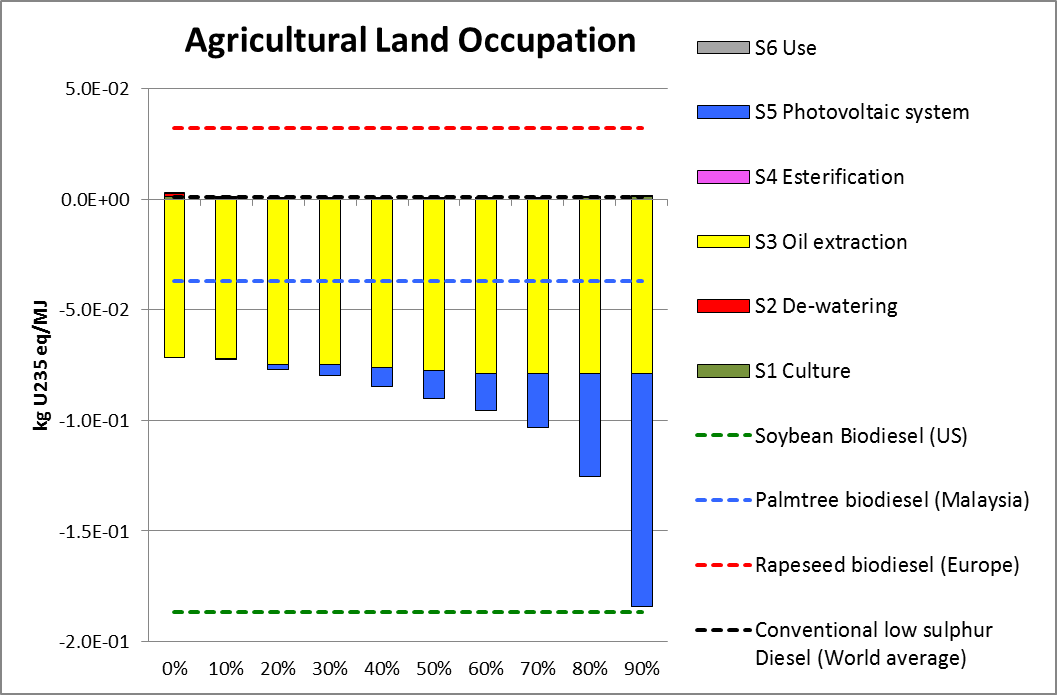


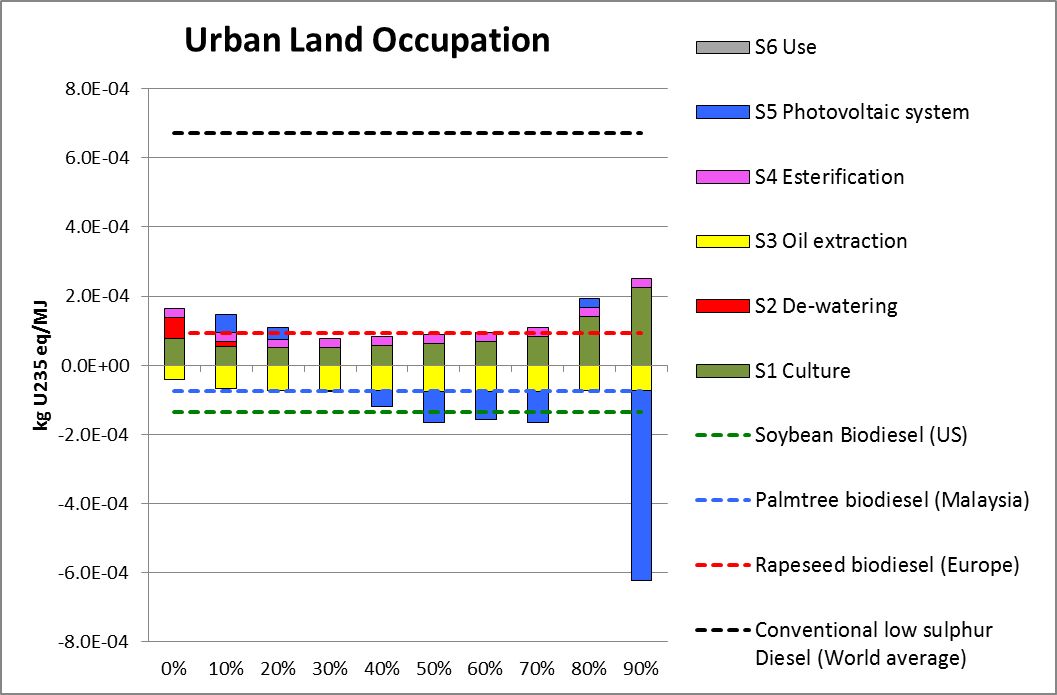


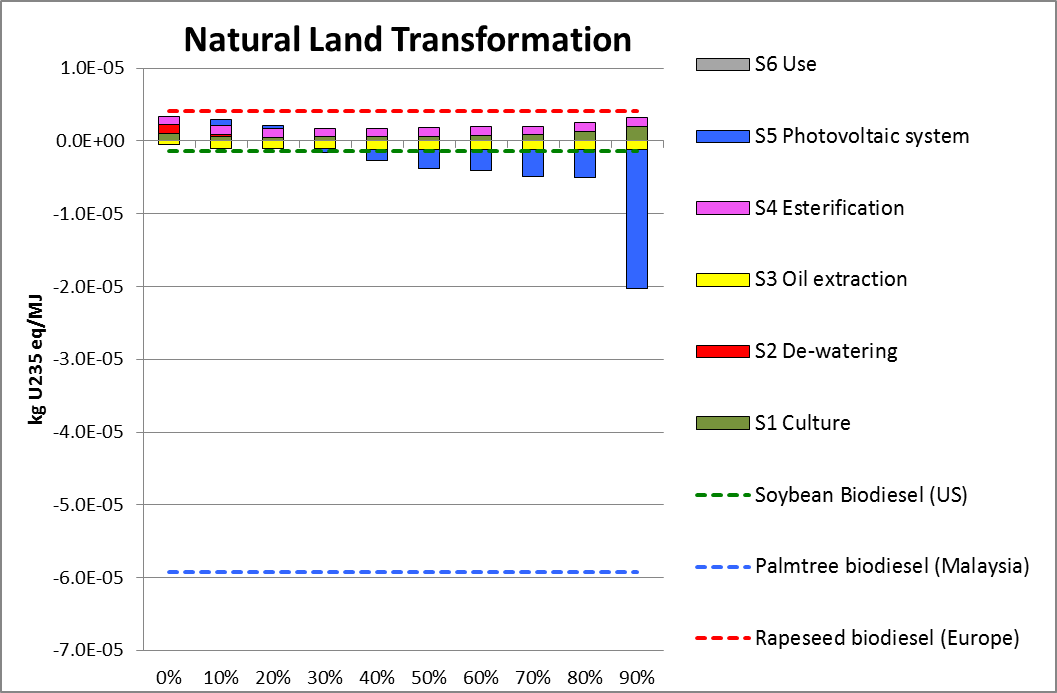


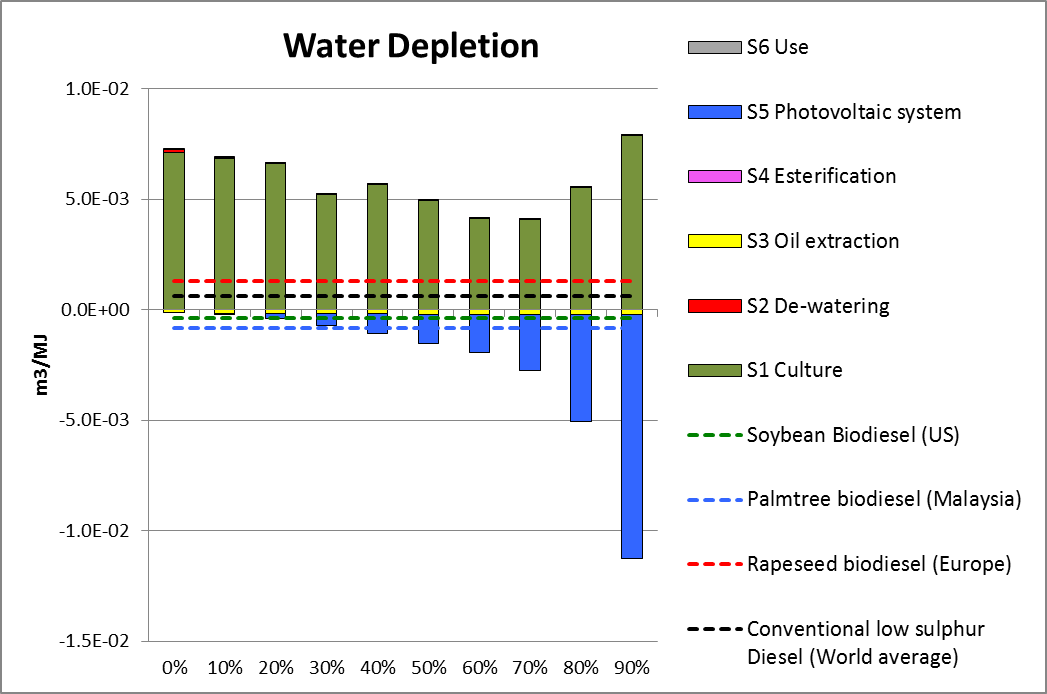


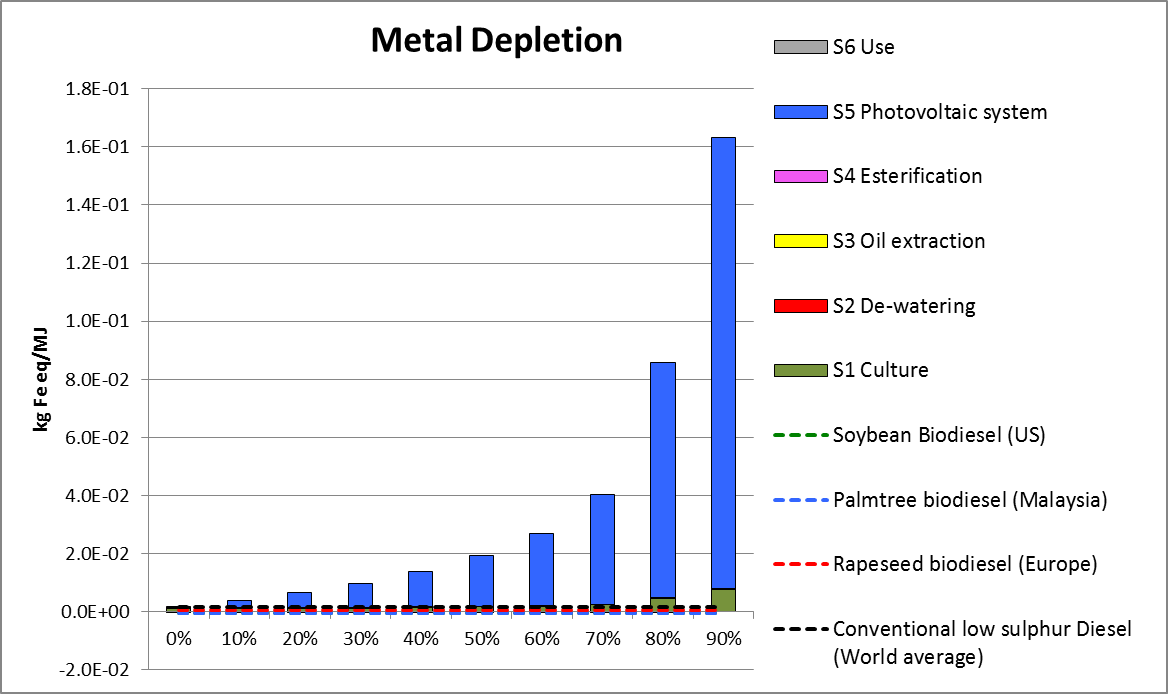


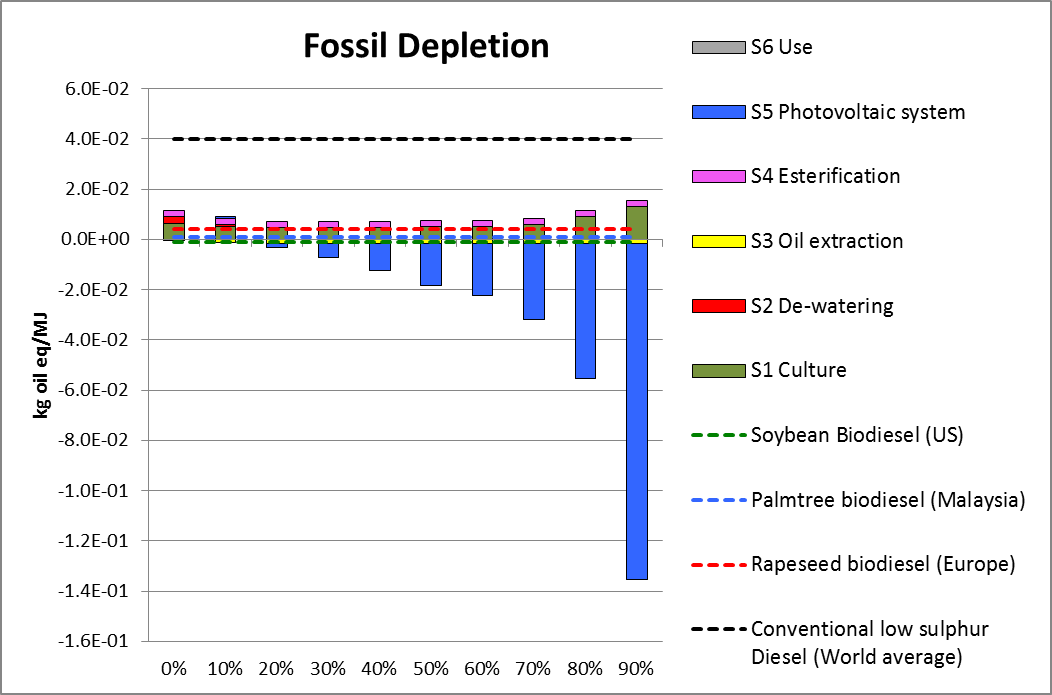


1. S20: Comparison LCA results between Energetic allocation and Substitution allocation method

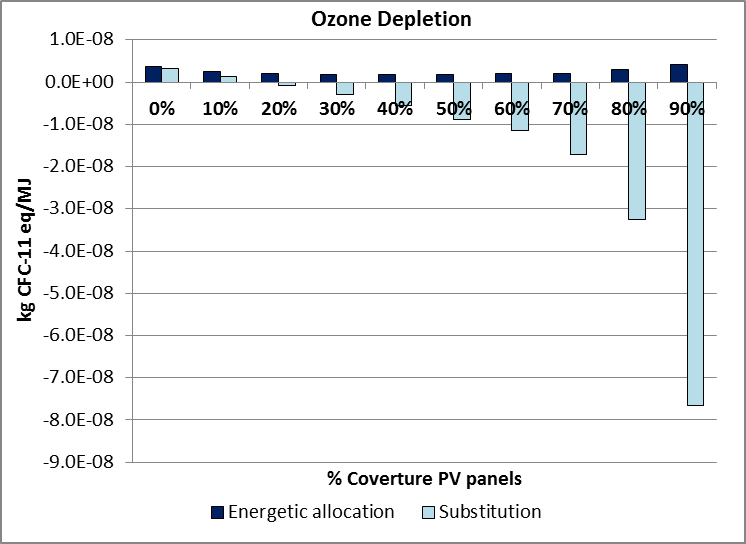


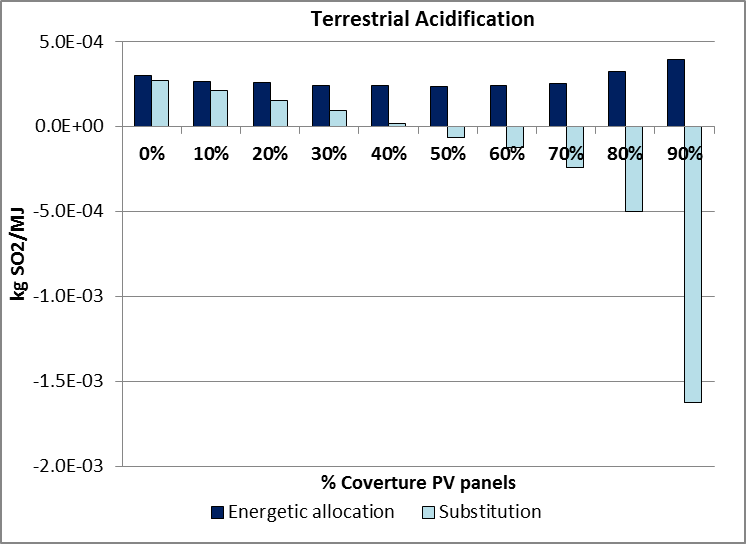


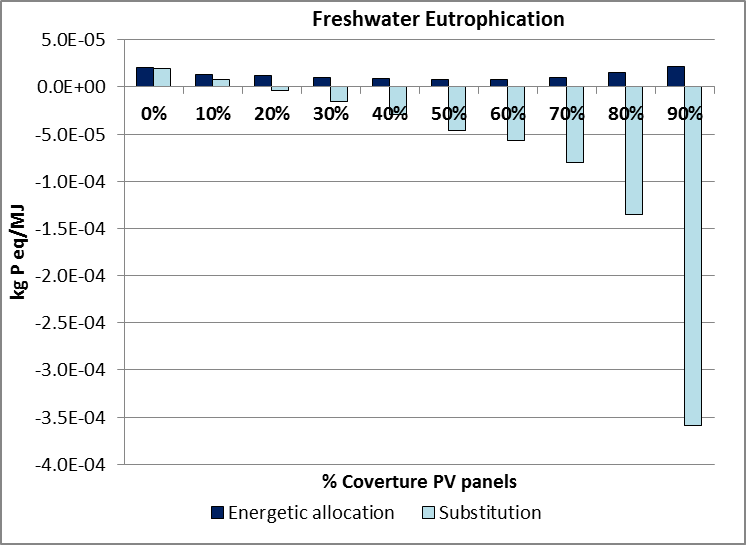


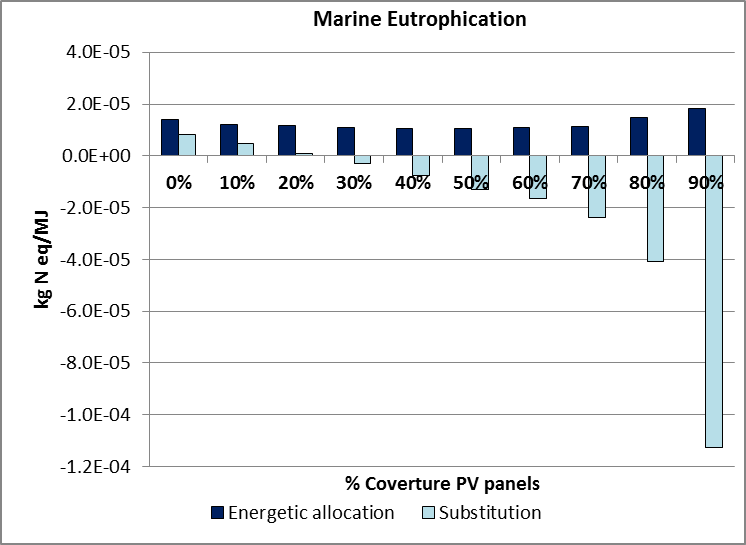


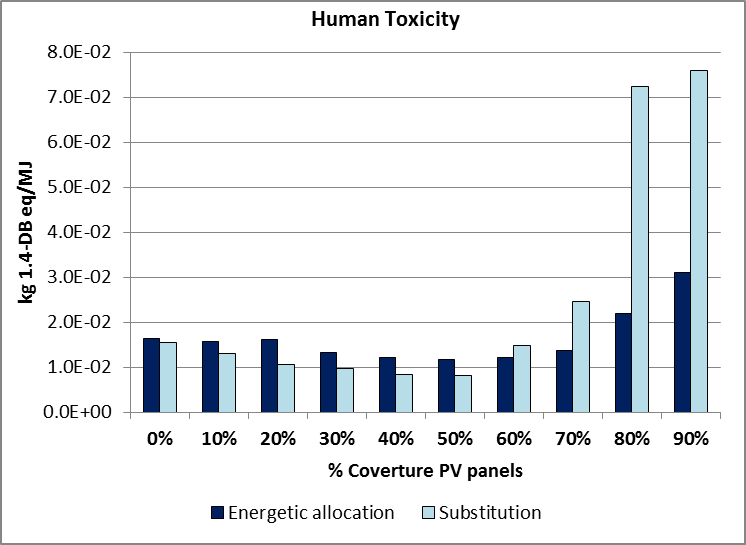


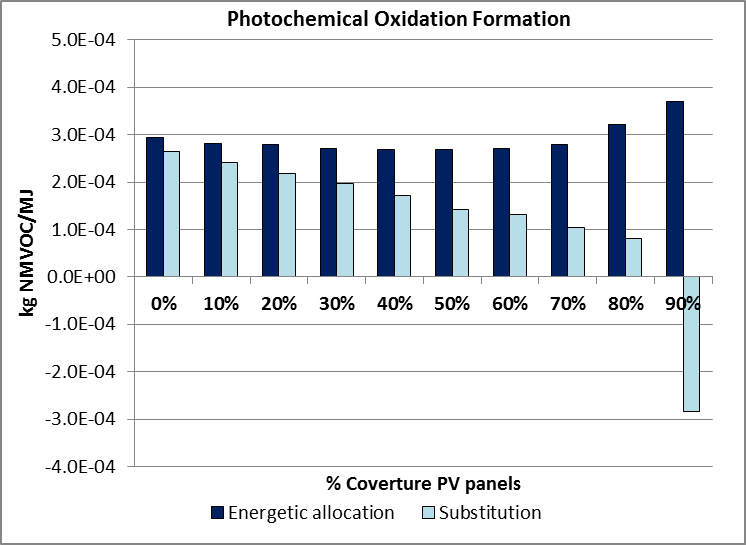


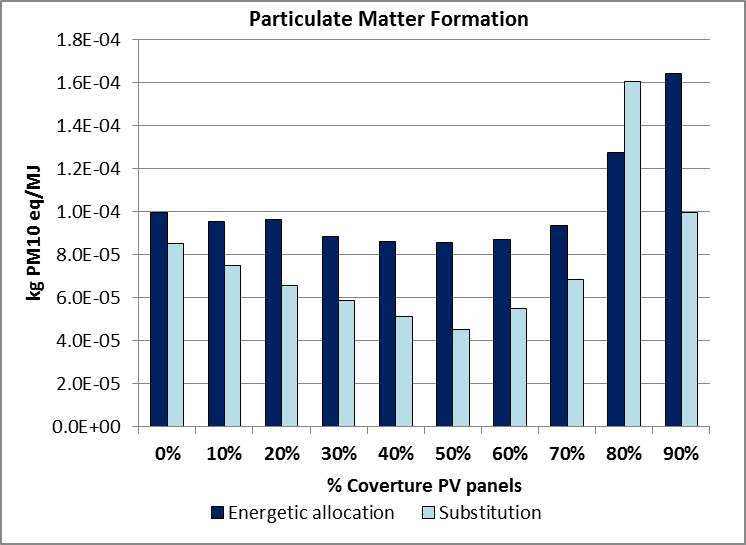


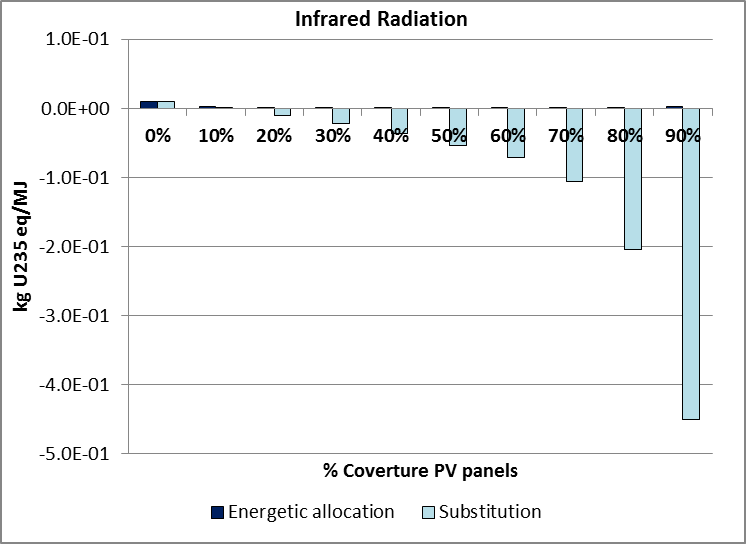


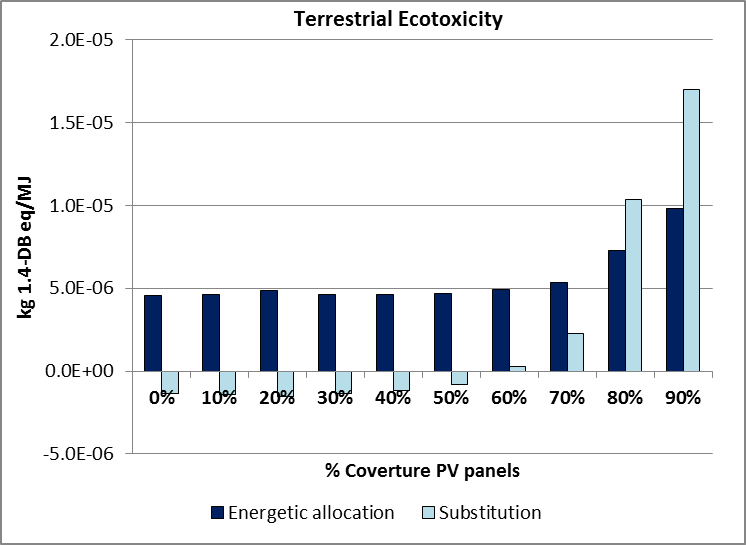


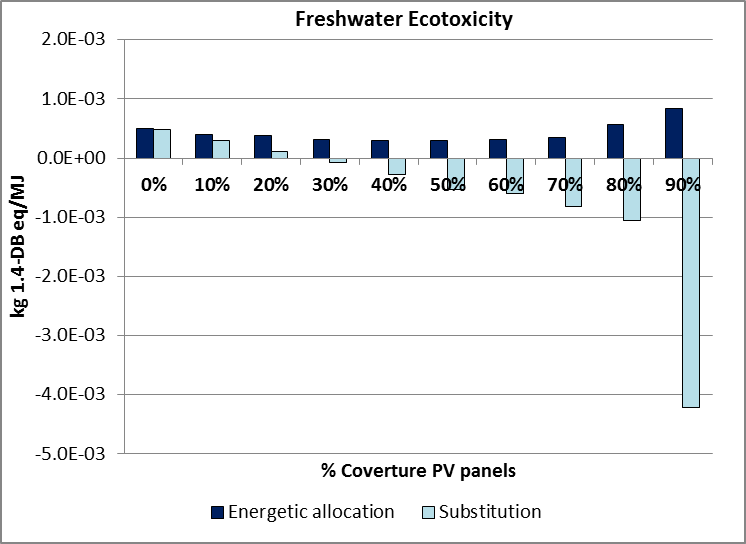


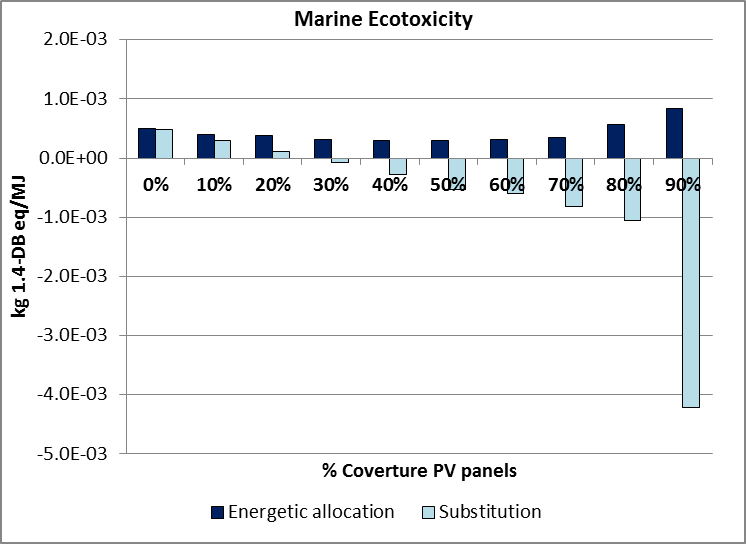


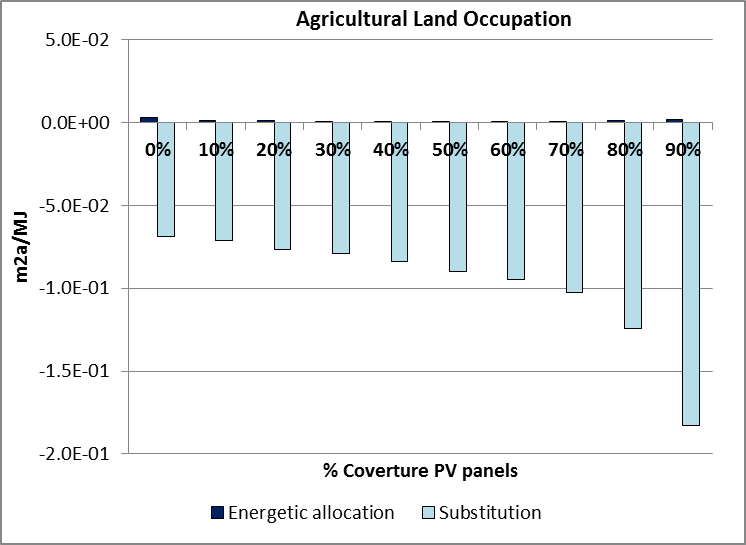


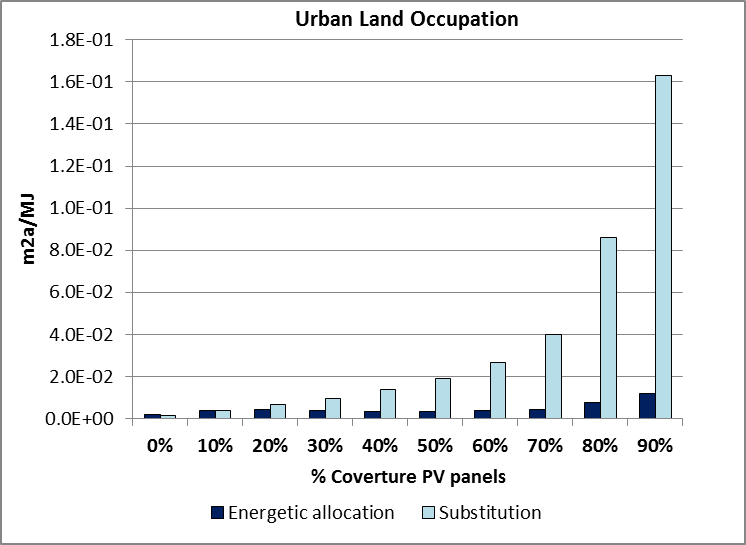


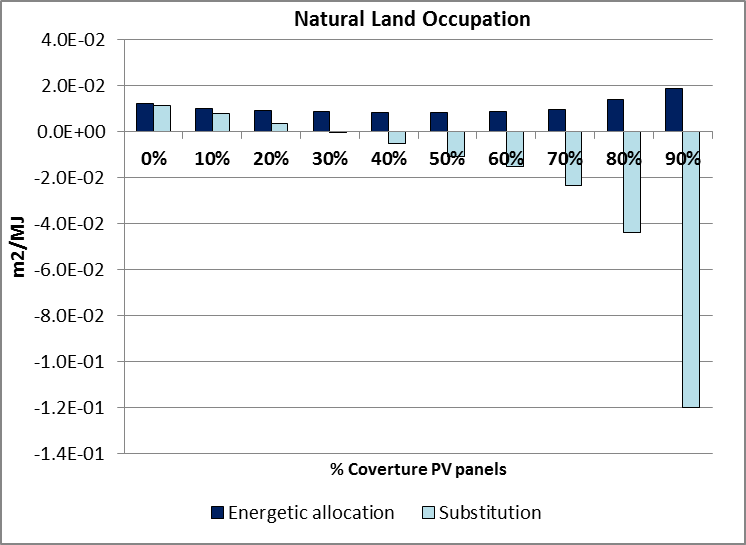


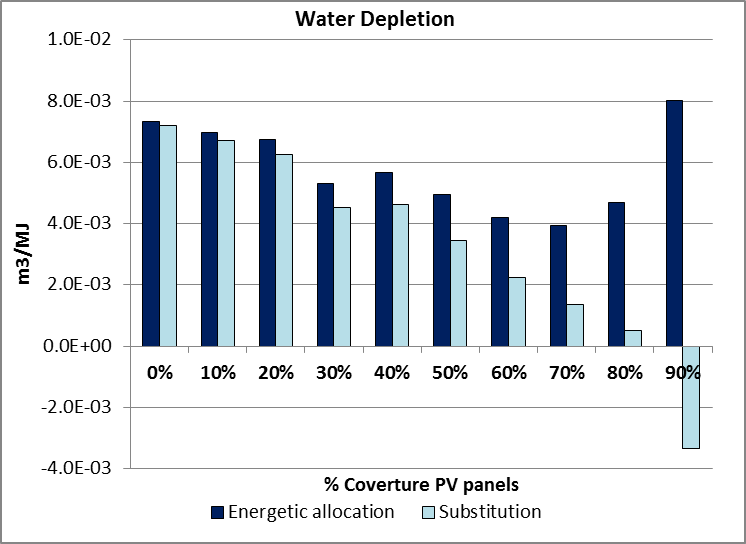


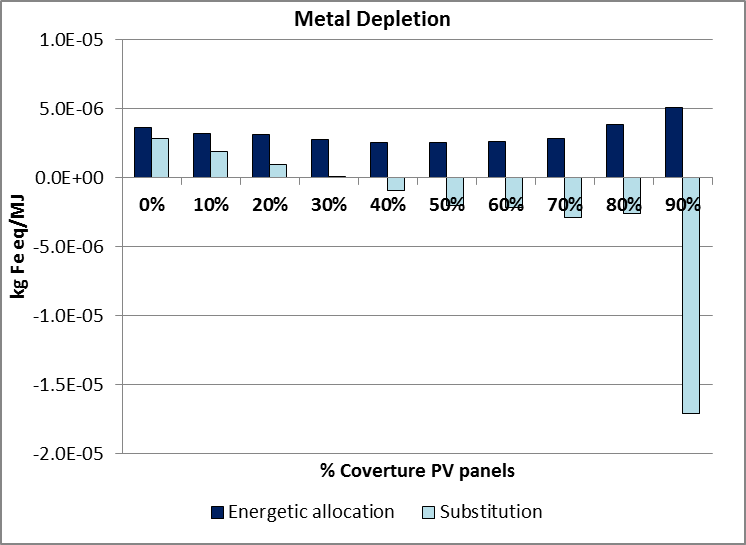


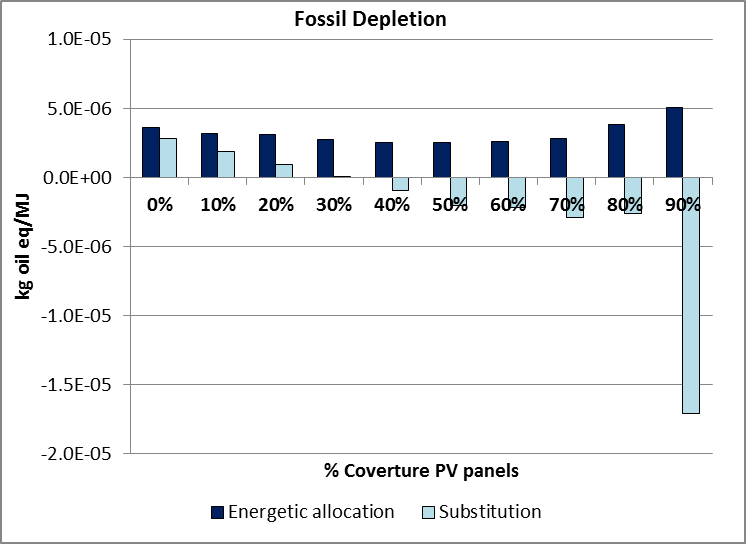


References

1. Pilkington. Pilkington Glass Handbook. England: Pilkington, 2010.

2. Li Y, Zhang Q, Zang Z, Wu X, Cong W. Evaluation of power consumption of paddle wheel in an open raceway pond. Bioprocess Biosyst Eng. 2014;37:1325-36.

3. Nord-Drivesystems. M7010. Smooth Surface Motors. Unique Washdown Design. Germany: NORD Gear Corporation. NORD Gear Limited, 2015 Report No.: 106701000 / 12.14.

4. Rogers J, Rosemberg J, Guzman B, Oh V, Mimbela L, Ghassemi A, et al. A critical analysis of paddlewheel-driven raceway ponds for algal biofuel production at commercial scales. Algal Res. 2014;4:76-88.

5. Chisti Y. Biodiesel from microalgae beats bioethanol. Trends Biotechnol. 2008;26(3):126-31.

6. Collet P, Lardon L, Hélias A, Bricout S, Lombaert-Valot I, Perrier B, et al. Biodiesel from microalgae - Life cycle assessment and recommendations for potential improvements. Renew Energ. 2014;71:525-33.

7. NREL. Process Design and Economics for the Production of Algal Biomass: Algal Biomass Production in Open Pond Systems and Processing Through Dewatering for Downstream Conversion. Golden, CO U.S. Department of Energy Office of Energy Efficiency & Renewable Energy, 2016 February. Report No.: NREL/TP-5100-64772.

8. Beal C, Gerber L, Sills D, Huntley M, Machesky S, Walsh M, et al. Algal biofuel production for fuels and feed in a 100-ha facility: A comprehensive techno-economic analysis and life cycle assessment. Algal Res. 2015;10:266-79.

9. Ota M, Takenaka M, Sato Y, Lee R, Inomata H. Effects of light intensity and temperature on photoautotrophic growth of a green microalga, Chlorococcum littorale. Biotechnology Reports. 2015;7:24-9.

10. Ho S, Chang J, Lai Y, Chen C. Achieving high lipid productivity of a thermotolerant microalga Desmodesmus sp. F2 by optimizing environmental factors and nutrient conditions. Bioresour Technol. 2014;156:108-16.

11. Huang C, Hung J, Peng S, Chen C. Cultivation of a thermo-tolerant microalga in an outdoor photobioreactor: Influences of CO2 and nitrogen sources on the accelerated growth. Bioresour Technol. 2012;112:228-33.

12. Kurano N, Miyachi S. Selection of Microalgal Growth Model for Describing Specific Growth Rate-Light Response Using Extended Information Criterion. Journal of Bioscience and Bioengineering. 2005;100(4):403-8.

13. Ho S, Chen C, Lai Y, Lu W, Chang J. Exploring the high lipid production potential of a thermotolerant microalga using statistical optimization and semi-continuous cultivation. Bioresour Technol. 2014;163:128-35.

14. Geider R, La Roche J. Redfield revisited: variability of C:N:P in marine microalgae and its biochemical basis. Eur J Phycol. 2002;37:1-17.

15. Adams C, Godfrey V, Wahlen B, Seefeldt L, Bugbee B. Understanding precision nitrogen stress to optimize the growth and lipid content tradeoff in oleaginous green microalgae. Bioresour Technol. 2013;131:188-94.

16. Chisti Y. Constraints to commercialization of algal fuels. J Biotechnol. 2013;167:201-14.

17. Chisti Y. Raceways-based production of algal crude oil. Green. 2013;3(3-4):195-216.

18. Pulz O. Photobioreactors: Production Systems for Photoautrophic Microorganisms. Appl Microbiol Biotechnol. 2001;57(3):287-93.

19. Chisti Y. Biodiesel from microalgae. Biotechnol Adv. 2007;25:294-306.

20. Haas M, McAloon A, Yee W, Foglia T. A process model to estimate biodiesel production costs. Bioresour Technol. 2006;97:671-8.

21. NREL. Process Design and Economics for the Conversion of Algal Biomass to Biofuels: Algal Biomass Fractionation to Lipid- and Carbohydrate-Derived Fuel Products. Golden, CO U.S. Department of Energy Office of Energy Efficiency & Renewable Energy, 2014 September. Report No.: NREL/TP-5100-62368.

22. Jungbluth N, Chudacoff M, Dauriat A, Dinkel F, Doka G, Faist-Enmenegger M, et al. Life cycle inventories of bioenergy. Final report ecoinvent data v2.0 Dübendorf: Swiss Centre for Life Cycle Inventories, 2007 Report No.: 17.

23. Gloria T, Lippiatt B, Cooper J. Life cycle impact assessment weight to support environmentally preferable purchasing in the United States. Environ Sci Technol. 2007;41(21):7551-7.

24. Panichelli L, Dauriat A, Gnansounou E. Life cycle assessment of soybean-based biodiesel in Argentina for export. Int J Life Cycle Ass. 2009;14:144-59.

25. Schmidt J. Comparison life cycle assessment of rapeseed oil and palm oil. Int J Life Cycle Ass. 2010;15:183-7.

26. Bernesson S. Life cycle assessment of rapeseed oil, rape methyl ester and ethanol as fuels - A comparison between large- and small- scale production. Uppsala: SLU. Department of Biometry and Engineering of Swedish University of Agricultural Sciences, 2004 December. Report No.: ISSN 1652 3237.

27. Kittithammavong V, Arpornpong N, Charoensang A, Khaodhiar S. Environmental Life Cycle Assessment of Palm oil- based Biofuel Production from Transesterification: Greenhouse Gas, Energy

and Water Balances. International Conference on Advances in Engineering and Technology (ICAET); March 29-40; Singapore. 2014. p. 615-21.

28. Castanheira E, Freire F. GHG LCA of soybean-based biodiesel. The implications of alternative LUC scenarios. Workshop on Quantifiyng and Managing Land Use Effects of Bioenergy; September 19-21; Campinas. 2011.

29. D`Avino L, Dainelli R, Lazzeri L, Spugnoli P. The role of co-products in biorefinery sustainability: energy allocation versus substitution method in rapeseed and carinata biodiesel chains. J Clean Prod. 2015;94:108-15.

30. Dones R, Bauer C, Bolliger R, Burger B, Heck T, Röder A, et al. Life cycle Inventories of Energy Systems: Results for Current Systems in Switzerland and others UCTE countries. Data v2.0. Ecoinvent Report Dübendorf: Swiss Centre for Life Cycle Inventories, 2007 December. Report No.: 5.
